# Supplementary material for: Transcriptome Changes in the Mink Uterus during Blastocyst Dormancy and Reactivation
Source: Int J Mol Sci. 2019 Apr 28;20(9):2099. doi: 10.3390/ijms20092099 (PMC6540205; doi:10.3390/ijms20092099)
Supplement: Supplementary file 1 [file ijms-20-02099-s001.pdf]

Differential Genes					
gene ID	log2Fold Change	pval	padj	Gene_title	Symbol
Cluster-62068.91323	7.9822	6.52E-42	9.39E-37	ankyrin repeat and sterile alpha motif domain containing 1A (ANKS1A)	ANKS1A
Cluster-62068.96708	7.4272	2.01E-33	7.24E-29	laminin, alpha 3 (LAMA3)	LAMA3
Cluster-62068.72330	6.9436	1.04E-27	1.87E-23	paroxysmal nonkinesigenic dyskinesia (PNKD)	PNKD
Cluster-62068.112831	6.4222	6.67E-29	1.60E-24	pyruvate dehydrogenase phosphatase regulatory subunit (PDPR)	PDPR
Cluster-62068.63548	6.3617	4.02E-21	4.83E-17	cleavage and polyadenylation specific factor 3(CPSF3)	CPSF3, YSH1
Cluster-62068.87900	6.3151	6.04E-23	9.65E-19	RIO kinase 3 (RIOK3)	RIOK3
Cluster-62068.75621	6.2389	3.67E-22	4.80E-18	THO complex 1 (THOC1)	THOC1
Cluster-62068.94740	6.1613	4.70E-21	5.20E-17	BAC RP11-489N6	RP11-489N6
Cluster-62068.93350	6.033	3.31E-28	6.80E-24	desmoglein 2 (DSG2)	DSG2
Cluster-62068.104440	6.0189	2.64E-17	1.66E-13	solute carrier family 7 (cationic amino acid transporter, y+ system), member 2 (SLC7A2)	SLC7A2, ATRC2
Cluster-62068.113897	5.9385	6.62E-18	4.77E-14	asp (abnormal spindle) homolog, microcephaly associated (Drosophila) (ASPM)	ASPM, ASP
Cluster-62068.115994	5.9165	1.29E-18	1.03E-14	immune regulator 1, ATPase, H+ transporting, lysosomal V0 subunit A3 (TCIRG1)	ATPeV0A, ATP6N
Cluster-62068.84799	5.9001	3.30E-19	2.97E-15	ZXD family zinc finger C (ZXDC)	ZXDC
Cluster-62068.95720	5.6566	6.67E-17	3.84E-13	protein disulfide isomerase family A, member 6 (PDIA6)	PDIA6, TXNDC7
Cluster-62068.65928	5.6026	9.71E-15	3.59E-11	sphingomyelin synthase 1 (SGMS1)4	SGMS
Cluster-62068.142479	5.5954	2.38E-15	1.07E-11	family with sequence similarity 124A (FAM124A)	FAM124A
Cluster-62068.101985	5.5944	2.84E-18	2.15E-14	Canis familiaris chromosome 16, clone XX-202C11	--
Cluster-62068.93490	5.5547	2.63E-16	1.35E-12	arginase 2 (ARG2)	rocF, arg
Cluster-62068.113160	5.451	9.67E-15	3.59E-11	transmembrane protein 248 (TMEM248)	TMEM248

Cluster-62068.133873	5.3432	7.27E-15	2.91E-11	DEAD/H (Asp-Glu-Ala-Asp/His) box helicase 11 (DDX11)	DDX11, CHL1, CTF1
Cluster-62068.102207	5.3359	8.09E-15	3.15E-11	nucleoporin 205kDa (NUP205)	NUP205, NUP192
Cluster-62068.130670	5.3067	5.99E-15	2.54E-11	raftlin family member 2 (RFTN2)	RFTN2
Cluster-62068.36514	5.261	3.88E-14	1.21E-10	Ursus maritimus methyltransferase like 25 (METTL25)	METTL25
Cluster-62068.69146	5.2067	1.14E-12	2.46E-09	heat shock 70kDa protein 12A (HSPA12A)	HSPA12A
Cluster-62068.113256	5.1693	1.84E-13	5.11E-10	aminomethyltransferase (AMT)	gcvT, AMT
Cluster-62068.98101	5.1171	8.00E-14	2.35E-10	phospholipase C, delta 1 (PLCD1)	PLCD
Cluster-62068.103189	5.0682	1.88E-13	5.12E-10	v-akt murine thymoma viral oncogene homolog 1 (AKT1)	AKT
Cluster-62068.99389	5.068	9.37E-14	2.70E-10	uncharacterized LOC101684958 (LOC101684958), ncRNA	LOC101684958
Cluster-62068.72605	5.0428	3.03E-13	7.76E-10	forkhead box N2 (FOXN2), transcript variant X4	CSDA, ZONAB
Cluster-62068.118515	4.9965	6.17E-12	1.18E-08	Canis familiaris chromosome 11, clone XX-368E15	--
Cluster-62068.82586	4.9752	9.67E-12	1.70E-08	small G protein signaling modulator 1 (SGSM1)	SGSM1
Cluster-62068.69968	4.9388	1.47E-12	3.03E-09	Odobenus rosmarus divergens protein phosphatase 2(PPP2R3B)	PPP2R3
Cluster-62068.128605	4.9213	9.04E-12	1.63E-08	transcriptional adaptor 2A (TADA2A)	TADA2A
Cluster-62068.25450	4.9142	2.02E-12	4.09E-09	RecQ mediated genome instability 1 (RMI1)	RMI1, BRAP75
Cluster-62068.117061	4.8934	1.58E-11	2.65E-08	Ursus maritimus dedicator of cytokinesis 9 (DOCK9)	DOCK9
Cluster-62068.141855	4.853	7.06E-12	1.30E-08	ELOVL fatty acid elongase 6 (ELOVL6)	ELOVL6
Cluster-62068.109638	4.8509	4.88E-12	9.50E-09	protein kinase N3 (PKN3)	PKN
Cluster-62068.138386	4.8282	2.03E-11	3.25E-08	phosphatidylinositol-4,5-bisphosphate 3-kinase (PIK3CA)	PIK3C
Cluster-62068.116555	4.826	1.58E-11	2.65E-08	Odobenus rosmarus divergens TBC1 domain family(TBC1D19)	TBC1D19
Cluster-62068.50694	4.7736	1.81E-11	2.96E-08	netrin G2 (NTNG2)	NTNG2
Cluster-62068.96445	4.7527	5.31E-16	2.64E-12	Canis familiaris chromosome 16	--
Cluster-62068.113790	4.7304	1.60E-11	2.65E-08	Ursus maritimus family with sequence similarity 49(FAM49A)	FAM49A
Cluster-62068.85901	4.7234	4.82E-11	6.81E-08	dystrophia myotonica-protein kinase (DMPK)	DMPK

Cluster-62068.80031	4.6976	2.69E-11	4.12E-08	UbiA prenyltransferase domain containing 1 (UBIAD1)	UBIAD1
Cluster-62068.105290	4.6567	3.91E-11	5.86E-08	peroxidasin (PXDN)	PXDN
Cluster-62068.134771	4.6379	4.75E-11	6.77E-08	family with sequence similarity 193, member A (FAM193A)4	FAM193A
Cluster-62068.145738	4.6222	2.19E-10	2.55E-07	zinc finger protein 532 (ZNF532)	ZNF532
Cluster-62068.69866	4.6209	1.20E-10	1.54E-07	NOP2/Sun RNA methyltransferase family, member 2 (NSUN2)	NSUN2
Cluster-62068.104641	4.6151	9.04E-10	8.86E-07	neuronal PAS domain protein 1 (NPAS1)	NPAS1_3
Cluster-62068.106879	4.6134	2.40E-13	6.29E-10	minichromosome maintenance complex component 3 associated protein (MCM3AP)	MCM3AP
Cluster-62068.120676	4.6098	1.01E-10	1.33E-07	mitogen-activated protein kinase binding protein 1 (MAPKBP1)	MAPKBP1
Cluster-62068.70905	4.6001	1.07E-10	1.39E-07	RNA pseudouridylate synthase domain containing 4 (RPUSD4)	RPUSD4
Cluster-62068.96447	4.5986	3.95E-14	1.21E-10	Canis familiaris chromosome 16, clone XX-202C11	--
Cluster-62068.94417	4.5846	2.55E-10	2.91E-07	laminin, alpha 5 (LAMA5)	LAMA3_5
Cluster-62068.148302	4.578	1.72E-10	2.12E-07	cell adhesion molecule L1-like (CHL1)	CHL1
Cluster-62068.52575	4.5758	1.38E-10	1.73E-07	coiled-coil domain containing 102A (CCDC102A)	CCDC102A
Cluster-62068.123308	4.5556	2.63E-10	2.96E-07	Odobenus rosmarus divergens very low density lipoprotein receptor (VLDLR)	VLDLR
Cluster-62068.116615	4.5524	1.87E-10	2.24E-07	3 BAC RP11-158G18 (Roswell Park Cancer Institute Human BAC Library) complete sequence	--
Cluster-62068.66814	4.5202	2.01E-10	2.39E-07	tetratricopeptide repeat domain 39A (TTC39A)	TTC39A
Cluster-62068.112364	4.5101	4.16E-10	4.41E-07	nuclear body protein SP140-like protein (LOC101693643)	--
Cluster-62068.104204	4.5031	2.85E-10	3.11E-07	bromodomain containing 4 (BRD4)	BRD4
Cluster-62068.57468	4.4973	3.61E-10	3.88E-07	establishment of sister chromatid cohesion N-acetyltransferase 1 (ESCO1)	ESCO, ECO1
Cluster-62068.82012	4.4859	7.16E-10	7.16E-07	minichromosome maintenance complex component 2 (MCM2)	MCM2
Cluster-62068.54559	4.4588	1.36E-09	1.28E-06	zinc finger, C2HC-type containing 1A (ZC2HC1A)	ZC2HC1A
Cluster-62068.57003	4.445	5.95E-10	6.12E-07	transmembrane protein 161B (TMEM161B)	TMEM161B
Cluster-62068.127919	4.4335	1.76E-09	1.61E-06	3-phosphoinositide dependent protein kinase 1 (PDPK1)	PDPK1

Cluster-62068.28899	4.4247	9.31E-10	9.06E-07	ret proto-oncogene (RET)	RET
Cluster-62068.149994	4.4154	6.85E-10	6.95E-07	von Willebrand factor A domain containing 5B2 (VWA5B2)	VWA5B2
Cluster-62068.116244	4.3756	2.02E-09	1.80E-06	uncharacterized LOC106004386 (LOC106004386)	EEF1D
Cluster-62068.77978	4.3462	2.45E-09	2.14E-06	von Willebrand factor A domain containing 5B2 (VWA5B2)	VWA5B2
Cluster-62068.121428	4.3462	1.70E-09	1.57E-06	Human DNA sequence from clone RP11-500G10 on chromosome 10	--
Cluster-62068.119842	4.3306	3.41E-13	8.46E-10	SWI/SNF related, matrix associated, actin dependent regulator of chromatin, subfamily c, member 2 (SMARCC2)	SMARCC
Cluster-62068.26933	4.3147	5.30E-09	4.26E-06	transcription termination factor, RNA polymerase II (TTF2)	TTF2
Cluster-62068.152129	4.3057	3.39E-09	2.85E-06	ral guanine nucleotide dissociation stimulator-like 3 (RGL3)	RGL3
Cluster-62068.171727	4.3049	3.17E-09	2.72E-06	Odobenus rosmarus divergens membrane metallo-endopeptidase-like 1 (MMEL1)	MMEL1
Cluster-62068.141788	4.2961	3.82E-09	3.20E-06	RAB36, member RAS oncogene family (RAB36)	RAB36
Cluster-62068.114647	4.2564	5.63E-09	4.40E-06	hydroxyacyl-CoA dehydrogenase/3-ketoacyl-CoA thiolase/enoyl-CoA hydratase (trifunctional protein) (HADHB)	HADHB
Cluster-62068.71823	4.2516	5.45E-09	4.33E-06	coiled-coil domain containing 151 (CCDC151)	CCDC151
Cluster-62068.92511	4.2489	2.41E-08	1.48E-05	Canis familiaris chromosome 16	--
Cluster-62068.114552	4.2479	5.59E-09	4.40E-06	cleavage and polyadenylation factor I subunit 1 (CLP1)	CLP1, HERB
Cluster-72532.0	4.2387	5.16E-09	4.17E-06	DTW domain containing 2 (DTWD2)	DTWD2
Cluster-62068.178785	4.2336	4.78E-09	3.91E-06	ferredoxin-fold anticodon binding domain containing 1 (FDXACB1)	FDXACB1
Cluster-62068.75403	4.2264	6.18E-09	4.79E-06	ADAM metallopeptidase with thrombospondin type 1 motif(ADAMTS9)	ADAMTS9
Cluster-62068.90323	4.222	2.39E-08	1.47E-05	mitochondrial calcium uptake 1 (MICU1)	MICU1
Cluster-62068.98794	4.1869	1.88E-08	1.22E-05	spectrin, alpha, non-erythrocytic 1 (SPTAN1)3	SPTAN1
Cluster-62068.119297	4.1514	2.85E-08	1.71E-05	protein regulator of cytokinesis 1 (PRC1)	PRC1
Cluster-62068.182487	4.1471	1.22E-08	8.35E-06	bone morphogenetic protein/retinoic acid inducible neural-specific 3	BRINP3

				(BRINP3)	
Cluster-62068.49400	4.1282	1.77E-08	1.16E-05	protein tyrosine phosphatase, receptor type (PTPRR)	PTPRR
Cluster-62068.93588	4.1216	1.96E-08	1.26E-05	leucine rich repeat containing 45 (LRRC45)	LRRC45
Cluster-62068.117721	4.1115	3.15E-08	1.85E-05	pappalysin 2 (PAPPA2)	PAPPA2
Cluster-62068.132888	4.1025	2.19E-08	1.36E-05	HAUS augmin-like complex (HAUS5)	HAUS5
Cluster-62068.131598	4.0844	2.11E-08	1.33E-05	TNF receptor-associated protein 1 (TRAP1)	TRAP1, HSP75
Cluster-62068.146019	4.0589	4.36E-08	2.46E-05	KRAB-A domain containing 2 (KRBA2)	KRBA2
Cluster-62068.89655	4.0453	6.93E-12	1.30E-08	Leptonychotes weddellii collagen alpha-1(IV) chain-like (LOC102729200)	COL4A
Cluster-62068.49587	4.0388	6.14E-08	3.33E-05	Odobenus rosmarus divergens tyrosylprotein sulfotransferase 1 (TPST1)	TPST
Cluster-62068.159161	4.0344	8.48E-09	6.10E-06	F-box and leucine-rich repeat protein 8 (FBXL8)	FBXL8
Cluster-62068.97581	4.0051	8.06E-08	4.22E-05	myosin VI (MYO6)	MYO6
Cluster-62068.52700	4.0035	4.95E-08	2.76E-05	fibroblast growth factor receptor substrate 3 (FRS3)	FRS3
Cluster-62068.136366	4.0015	1.10E-07	5.64E-05	prolactin receptor (PRLR)	PRLR
Cluster-62068.57912	3.984	7.44E-08	3.97E-05	ankyrin repeat domain 34B (ANKRD34B)	ANKRD34B
Cluster-62068.139592	3.972	6.58E-08	3.53E-05	Ursus maritimus beta-1,4-N-acetyl-galactosaminyl transferase 3 (B4GALNT3)	--
Cluster-62068.48372	3.957	2.08E-07	9.35E-05	12 BAC RP11-285E4	--
Cluster-62068.147726	3.9537	1.11E-07	5.64E-05	notch 4 (NOTCH4)	NOTCH
Cluster-62068.61752	3.9292	2.12E-07	9.49E-05	storkhead box 2 (STOX2)	STOX2
Cluster-62068.130317	3.9229	1.12E-07	5.71E-05	Rho-associated, coiled-coil containing protein kinase 2 (ROCK2)	ROCK2
Cluster-62068.176344	3.9191	1.18E-07	5.88E-05	Ursus maritimus F-box protein 48 (FBXO48)	FBXO48
Cluster-62068.22116	3.9087	1.19E-07	5.90E-05	tachykinin, precursor 1 (TAC1)	TAC1
Cluster-62068.67443	3.907	1.17E-07	5.84E-05	Ovis canadensis canadensis isolate 43U chromosome 25 sequence	--
Cluster-62068.120962	3.9059	1.93E-07	8.83E-05	aldo-keto reductase family 1, member E2 (AKR1E2)	AKR1E2, AKR1CL2

Cluster-62068.177222	3.8988	1.30E-07	6.30E-05	ATP synthase mitochondrial F1 complex assembly factor 2 (ATPAF2), transcript variant X4	ATPeAF2, ATPAF2, ATP12
Cluster-62068.98283	3.8798	1.46E-07	6.92E-05	Ailuropoda melanoleuca zinc finger homeobox 3 (ZFHX3)	ATBF1
Cluster-62068.51442	3.8795	7.54E-08	3.99E-05	Schistosoma curassoni genome assembly S_curassoni_Dakar ,scaffold SCUD_contig0019022	--
Cluster-79064.0	3.8641	2.08E-07	9.35E-05	ankyrin repeat domain 55 (ANKRD55)	ANKRD55
Cluster-62068.154865	3.8415	2.34E-07	0.00010236	leucine rich repeat containing 42 (LRRC42)	LRRC42
Cluster-62068.97881	3.8289	3.01E-07	0.00012608	collagen, type I, alpha 1 (COL1A1)	COL1AS
Cluster-62068.131748	3.8283	2.83E-07	0.00012032	neuronal cell adhesion molecule (NRCAM)2	NRCAM
Cluster-62068.149497	3.8273	2.69E-10	3.00E-07	LanC lantibiotic synthetase component C-like 1 (LANCL1)	LANCL1
Cluster-62068.93964	3.8183	2.89E-07	0.00012154	cryptochrome circadian clock 2 (CRY2)	CRY
Cluster-62068.168311	3.8051	3.56E-07	0.00014334	opioid binding protein/cell adhesion molecule-like (OPCML), transcript variant X4	OPCML
Cluster-62068.22254	3.79	9.64E-07	0.00033484	uteroferrin-associated basic protein 2-like (LOC101674813)	--
Cluster-62068.191461	3.7796	3.70E-07	0.00014858	MACACA MULATTA BAC clone CH250-201N11 from chromosome 12	--
Cluster-62068.92521	3.7784	1.42E-07	6.77E-05	solute carrier family 16, member 14 (SLC16A14)	SLC16A14
Cluster-62068.23008	3.7724	5.41E-07	0.00020905	protein tyrosine phosphatase, receptor type (PTPRC)	PTPRC, CD45
Cluster-62068.99369	3.7714	9.10E-07	0.00031958	prolactin receptor (PRLR)	PRLR
Cluster-62068.92107	3.7533	4.63E-07	0.00018113	angiotensin I converting enzyme 2 (ACE2)	ACEH, ACE2
Cluster-62068.138056	3.752	1.35E-06	0.00043993	coiled-coil domain containing 15 (CCDC15)	CCDC15
Cluster-62068.89353	3.7428	5.38E-09	4.30E-06	solute carrier family 4, sodium bicarbonate cotransporter, member 8 (SLC4A8)	SLC4A8
Cluster-62068.155377	3.7252	7.04E-07	0.00026059	Felis catus BAC clone FCAB-55H12 from chromosome unknown	--
Cluster-62068.106503	3.7222	6.88E-07	0.00025589	RAS p21 protein activator (GTPase activating protein) 1 (RASA1)	RASA1, RASGAP

Cluster-62068.107771	3.7146	3.94E-07	0.00015486	serpin peptidase inhibitor, clade D (heparin cofactor), member 1 (SERPIND1)	SERPIND1, HCF2
Cluster-62068.137958	3.7054	7.49E-07	0.00027253	regulating synaptic membrane exocytosis 3 (RIMS3)	RIMS3
Cluster-62068.58929	3.7012	1.47E-06	0.00046644	Ursus maritimus solute carrier family 26 (anion exchanger), member 6 (SLC26A6)	SLC26A6
Cluster-62068.83389	3.6788	1.11E-06	0.00038164	sterile alpha motif domain containing 11 (SAMD11)	SAMD11
Cluster-62068.108350	3.6779	1.36E-06	0.00044151	glucosaminyl (N-acetyl) transferase 2, I-branching enzyme (I blood group) (GCNT2)	GCNT2
Cluster-62068.32243	3.6678	9.39E-07	0.00032829	Canis Familiaris chromosome 5, clone XX-10I19	--
Cluster-62068.133995	3.6626	1.91E-06	0.00057749	cell wall biogenesis 43 C-terminal homolog (CWH43)	--
Cluster-62068.65775	3.6599	1.18E-06	0.00039714	pleckstrin homology domain-containing family A member 7 (LOC101678352)	LOC101678352
Cluster-62068.17365	3.6493	1.16E-06	0.00039261	Ovis canadensis canadensis isolate 43U chromosome 3 sequence	--
Cluster-62068.129990	3.6485	1.26E-06	0.00041684	Ursus maritimus kelch domain containing 1 (KLHDC1)	KLHDC1
Cluster-62068.119960	3.6396	7.84E-07	0.00028287	DnaJ (Hsp40) homolog, subfamily C(DNAJC24)	DPH4, DNAJC24
Cluster-62068.33662	3.6311	1.28E-06	0.00041962	MACACA MULATTA BAC clone CH250-364H1 from chromosome 1	--
Cluster-62068.140683	3.6207	1.36E-06	0.00044151	ribosomal protein S6 kinase, 90kDa, polypeptide 1 (RPS6KA1)	RPS6KA, RSK2
Cluster-62068.84625	3.6203	9.14E-11	1.22E-07	growth differentiation factor 3 (GDF3)	GDF3
Cluster-62068.64181	3.6128	1.60E-06	0.00049571	inositol polyphosphate-5-phosphatase(INPP5D)	SHIP1, INPP5D
Cluster-62068.93181	3.6029	2.93E-08	1.74E-05	proteasome (prosome, macropain) (PSMA6)	PSMA6
Cluster-62068.109825	3.5875	2.57E-06	0.0007365	signal transducer and activator of transcription 2 (STAT2)	STAT2
Cluster-62068.69806	3.5765	3.27E-06	0.00090501	Canis familiaris chromosome 16, clone XX-202C11	--
Cluster-62068.75245	3.5737	2.39E-06	0.0006916	required for meiotic nuclear division 1 homolog (RMND1)	--
Cluster-62068.93416	3.5703	1.24E-10	1.58E-07	claudin 8 (CLDN8)	CLDN
Cluster-62068.96362	3.5656	2.67E-06	0.00076031	protein phosphatase 1, regulatory subunit 21 (PPP1R21)	PPP1R21, CCDC128,

					KLRAQ1
Cluster-62068.89888	3.5533	3.52E-06	0.00095769	vertebrae development associated (VRTN)	VRTN
Cluster-62068.59642	3.5488	3.83E-06	0.0010221	transmembrane protein 106A (TMEM106A)	TMEM106A
Cluster-62068.48534	3.5282	4.12E-06	0.0010845	dachsous cadherin-related 1 (DCHS1)	DCHS1_2, PCDH16_23
Cluster-62068.84530	3.5274	3.89E-06	0.0010356	collagen, type XV, alpha 1 (COL15A1)	COL15A1
Cluster-62068.116490	3.5266	3.21E-06	0.00089699	Ailuropoda melanoleuca zinc finger, DHHC-type containing 21 (ZDHHC21)	ZDHHC
Cluster-62068.155803	3.5203	7.88E-07	0.00028367	Ovis canadensis canadensis isolate 43U chromosome 1 sequence	SSX2IP, ADIP
Cluster-62068.105649	3.516	1.24E-07	6.12E-05	ST3 beta-galactoside alpha-2,3-sialyltransferase 4 (ST3GAL4)	SIAT4C
Cluster-62068.70969	3.5155	3.03E-06	0.00085293	SIL1 nucleotide exchange factor (SIL1)	SIL1
Cluster-62068.175605	3.5124	5.51E-06	0.0013717	kelch-like family member 41 (KLHL41)	KBTBD5_10
Cluster-62068.155391	3.5098	4.86E-06	0.0012351	family with sequence similarity 101(FAM101B)	--
Cluster-62068.188199	3.5096	3.62E-06	0.00097823	chromosome 17, clone RP11-357H14	RP11-357H14
Cluster-62068.158690	3.5059	1.22E-06	0.00041088	Canis familiaris, clone XX-7D6	--
Cluster-62068.77996	3.5021	7.40E-06	0.0017374	Bos taurus BAC CH240-237H4	--
Cluster-62068.82557	3.4972	6.09E-06	0.0014713	BRF2, RNA polymerase III transcription initiation factor 50 kDa subunit (BRF2)	BRF2
Cluster-62068.172942	3.4946	3.96E-06	0.0010495	Human DNA sequence from clone RP11-330A16 on chromosome 6	--
Cluster-62068.128709	3.4926	4.28E-06	0.0011174	growth regulation by estrogen in breast cancer-like (GREB1L)	GREB1L
Cluster-62068.43445	3.4913	3.74E-06	0.0010033	uncharacterized LOC106005543 (LOC106005543)	--
Cluster-62068.76480	3.4902	5.16E-06	0.0013018	origin recognition complex, subunit 4 (ORC4)2	ORC4
Cluster-62068.105816	3.4736	3.12E-07	0.00012944	solute carrier family 4, sodium bicarbonate cotransporter, member 8 (SLC4A8)	SLC4A8
Cluster-62068.48989	3.4573	6.03E-06	0.0014647	agmatine ureohydrolase (agmatinase) (AGMAT)	speB
Cluster-62068.46629	3.4489	5.49E-06	0.0013715	RAR-related orphan receptor A (RORA)	NR1F1, RORA

Cluster-62068.146217	3.4406	9.84E-06	0.002164	G protein-coupled receptor 143 (GPR143)	OA1, GPR143
Cluster-62068.45910	3.4395	5.97E-06	0.0014594	zinc finger protein 713 (ZNF713)	KRAB
Cluster-62068.100278	3.4369	8.69E-06	0.0019805	glycine amidinotransferase (L-arginine:glycine amidinotransferase) (GATM)	GATM
Cluster-62068.185558	3.4319	8.36E-06	0.0019288	chromosome unknown open reading frame, human C5orf28 (LOC101684264), transcript variant X4	--
Cluster-62068.54331	3.4265	7.28E-06	0.0017174	UPF2 regulator of nonsense transcripts homolog (yeast) (UPF2), RefSeqGene on chromosome 10	UPF2
Cluster-62068.81731	3.4248	6.34E-06	0.0015301	Panthera tigris altaica alkB, alkylation repair homolog 6 (E. coli) (ALKBH6)	ALKBH6
Cluster-62068.175432	3.4205	7.13E-06	0.0016873	janus kinase and microtubule interacting protein 2 (JAKMIP2), transcript variant X6	JAKMIP2
Cluster-62068.86384	3.4154	1.23E-06	0.00041119	Odobenus rosmarus divergens actin, alpha, cardiac muscle 1 (ACTC1)	ACTC1
Cluster-62068.118145	3.4117	7.41E-07	0.00027153	solute carrier family 26 (anion exchanger), member 4 (SLC26A4)	SLC26A4, PDS
Cluster-62068.74963	3.411	1.57E-06	0.00049438	Ovis canadensis canadensis isolate 43U chromosome 3 sequence	--
Cluster-62068.86016	3.4109	2.47E-06	0.00070839	phosphoglucomutase 1 (PGM1)	pgm
Cluster-62068.115828	3.4028	2.23E-09	1.96E-06	Meles meles leucurus isolate TB402b alpha cardiac actin (ACTC) gene, partial cds	ACTA2
Cluster-62068.159964	3.3999	8.17E-06	0.0018906	phosphodiesterase 11A (PDE11A)	PDE11
Cluster-62068.24024	3.3999	9.24E-06	0.0020821	DAZ interacting zinc finger protein 1-like (DZIP1L), transcript variant X6	DZIP1
Cluster-62068.162610	3.393	9.28E-06	0.0020873	Ailuropoda melanoleuca plexin C1 (PLXNC1)	PLXNC
Cluster-62068.65235	3.3898	9.42E-06	0.0021097	solute carrier family 4, sodium bicarbonate cotransporter, member 8 (SLC4A8)	SLC4A8
Cluster-62068.128533	3.3842	1.09E-05	0.0023374	5'-nucleotidase, cytosolic II (NT5C2)	NT5C2
Cluster-62068.102107	3.3839	8.87E-06	0.0020138	Leptonychotes weddellii protein phosphatase 1, catalytic subunit, gamma isozyme (PPP1CC)	PPP1C

Cluster-62068.89696	3.3804	1.32E-05	0.0027303	Ailuropoda melanoleuca tumor protein p53 inducible protein 11 (TP53I11)	--
Cluster-62068.94291	3.3801	1.13E-06	0.0003861	AT rich interactive domain 5A (MRF1-like) (ARID5A)	ARID5A
Cluster-62068.96463	3.3747	1.21E-09	1.16E-06	Sus scrofa mRNA, clone:AMP010013D11, expressed in alveolar macrophage	SNAPC4
Cluster-62068.24459	3.3718	9.16E-06	0.0020663	N-acetylneuraminate pyruvate lyase (dihydrodipicolinate synthase) (NPL)	E4.1.3.3, nanA, NPL
Cluster-62068.13533	3.3713	1.05E-05	0.0022714	Odobenus rosmarus divergens chromosome unknown open reading frame, human C9orf172 (LOC101371046)	--
Cluster-62068.57872	3.3672	2.30E-06	0.00066805	low density lipoprotein receptor-related protein 2 (LRP2)	LRP2
Cluster-62068.100249	3.3672	9.63E-06	0.0021363	chromosome unknown open reading frame, human C16orf93 (LOC101684016)	--
Cluster-62068.137392	3.365	3.45E-07	0.00014059	synaptosomal-associated protein, 25kDa (SNAP25)	SNAP25
Cluster-62068.88084	3.3561	4.82E-06	0.001229	solute carrier family 4, sodium bicarbonate cotransporter, member 8 (SLC4A8), transcript variant X7	SLC4A8
Cluster-62068.96228	3.3484	2.69E-06	0.00076475	carbonic anhydrase II (CA2)	CA2
Cluster-62068.146738	3.347	1.08E-05	0.0023245	polo-like kinase 3 (PLK3)	PLK3, CNK
Cluster-62068.152244	3.3437	1.45E-05	0.0029447	Rho GTPase activating protein 12 (ARHGAP12), transcript variant X6	ARHGAP12
Cluster-62068.116762	3.3361	2.64E-06	0.00075448	hexokinase domain containing 1 (HKDC1)	HK
Cluster-62068.110850	3.3352	3.65E-06	0.0009833	v-akt murine thymoma viral oncogene homolog 2 (AKT2), transcript variant X4	AGA, aspG
Cluster-62068.145250	3.3329	1.42E-05	0.0029061	zinc finger protein, FOG family member 1 (ZFPM1)	ZFPM1, FOG1
Cluster-62068.92666	3.3172	1.41E-05	0.0028945	Odobenus rosmarus divergens PDZ domain containing 1 (PDZK1)	PDZK1
Cluster-62068.23290	3.3166	1.41E-05	0.0028945	uncharacterized LOC101691309 (LOC101691309), ncRNA	--
Cluster-62068.18506	3.3139	1.49E-05	0.0029949	phosphatidylinositol-4-phosphate 3-kinase, catalytic subunit type 2 gamma (PIK3C2G), misc_RNA	PIK3C2
Cluster-62068.126321	3.3086	1.46E-05	0.00295	Human DNA sequence from clone RP11-336F23 on chromosome 1	--

Cluster-62068.44617	3.2946	1.69E-05	0.0033154	proprotein convertase subtilisin/kexin type 1 (PCSK1)	PCSK1
Cluster-62068.66463	3.2885	1.87E-05	0.003594	osteoclast associated, immunoglobulin-like receptor (OSCAR)	OSCAR
Cluster-62068.86163	3.2856	4.67E-13	1.14E-09	chromosome unknown open reading frame, human C20orf24 (LOC106003404)	C20orf24
Cluster-62068.61151	3.2622	2.10E-05	0.0039396	RP11-159D12	--
Cluster-62068.81431	3.261	3.10E-05	0.0053737	chromosome unknown open reading frame, human C4orf26 (LOC101671167)	--
Cluster-62068.75299	3.2598	2.85E-07	0.00012069	L-amino-acid oxidase-like (LOC101679701)	IL4I1
Cluster-62068.59171	3.2587	1.94E-05	0.0037049	glycerol kinase 5 (putative) (GK5)	GK5
Cluster-62068.18714	3.2554	1.18E-05	0.0024852	Leptonychotes weddellii tyrosine aminotransferase (TAT)	TAT
Cluster-62068.165119	3.2516	2.39E-05	0.004336	Pan troglodytes BAC clone CH251-15E3 from chromosome 15	--
Cluster-62068.40035	3.2514	2.18E-05	0.004042	Ovis aries musimon uncharacterized LOC105603321 (LOC105603321), transcript variant X7, misc_RNA	--
Cluster-62068.161255	3.2414	2.24E-06	0.0006552	endogenous retrovirus group K member 113 Pol protein-like (LOC106004070)	--
Cluster-29662.0	3.2371	2.66E-05	0.0047212	Canis Familiaris chromosome 37, clone XX-341P11	--
Cluster-62068.69119	3.2361	2.33E-05	0.0042518	proline-rich basic protein 1 (PROB1)	PROB1
Cluster-62068.164708	3.2346	2.62E-05	0.0046806	acetylserotonin O-methyltransferase (ASMT)	ASMT
Cluster-62068.153764	3.2336	3.94E-06	0.0010456	chromosome 15, clone RP11-151H2	PPCDC, coaC
Cluster-62068.77258	3.225	2.43E-05	0.0043915	CTTNBP2 N-terminal like (CTTNBP2NL)	CTTNBP2NL
Cluster-62068.65226	3.2194	3.02E-05	0.005238	E74-like factor 5 (ets domain transcription factor) (ELF5)	ELF5
Cluster-62068.22117	3.2191	3.37E-05	0.0057804	tachykinin, precursor 1 (TAC1)	TAC1
Cluster-68303.0	3.2164	1.38E-05	0.002848	chromosome 16 clone RP11-124K4	--
Cluster-62068.157950	3.1988	3.73E-05	0.0062056	Odobenus rosmarus divergens poly(rC) binding protein 3 (PCBP3), transcript variant X4	PCBP2_3_4
Cluster-62068.109894	3.1943	2.48E-05	0.0044716	Canis familiaris chromosome 16, clone XX-202C11	--

Cluster-62068.73831	3.1865	3.52E-05	0.0059419	ariadne RBR E3 ubiquitin protein ligase 1 (ARIH1)	ARIH1
Cluster-62068.114476	3.1834	4.56E-05	0.00725	cAMP responsive element modulator (CREM)3	CREM
Cluster-62068.63498	3.1796	3.41E-05	0.0058215	phospholipase C, beta 4 (PLCB4), transcript variant X8	PLCB
Cluster-62068.96843	3.1792	4.64E-05	0.007334	collagen, type III, alpha 1 (COL3A1)	COL1AS
Cluster-62068.122679	3.179	8.06E-06	0.0018695	actin, alpha, cardiac muscle 1 (ACTC1)	ACTC1
Cluster-62068.60720	3.1763	3.75E-05	0.0062247	transmembrane protein 39A (TMEM39A)	TMEM39A
Cluster-34356.0	3.1728	3.74E-05	0.0062128	solute carrier family 22 (organic cation/zwitterion transporter), member 4 (SLC22A4), transcript variant X4	SLC22A4_5, OCTN
Cluster-62068.99427	3.15	2.57E-07	0.00011035	Canis Familiaris chromosome 14, clone XX-483F1	--
Cluster-62068.66223	3.143	4.89E-05	0.0076412	alpha-2-macroglobulin-like 1 (A2ML1), transcript variant X4	A2ML1
Cluster-62068.11926	3.1364	4.40E-05	0.0070896	KIAA1211 ortholog (KIAA1211)	KIAA1211
Cluster-62068.26969	3.1346	2.49E-07	0.00010774	Canis lupus familiaris protein tyrosine phosphatase, receptor type, B (PTPRB)	--
Cluster-62068.100687	3.1264	4.93E-07	0.00019244	zyxin (ZYX)	FZD5_8, fz2
Cluster-62068.113766	3.1248	4.28E-05	0.0069244	paralemmin 3 (PALM3)	PALM3
Cluster-62068.147770	3.1219	6.85E-05	0.0099565	sperm associated antigen 5 (SPAG5)	SPAG5
Cluster-62068.62684	3.1208	5.13E-05	0.0079259	chromosome 16 clone RP11-98C8	--
Cluster-62068.78060	3.1178	3.23E-05	0.0055753	cytohesin 1 (CYTH1), transcript variant X4	CYTH
Cluster-62068.84841	3.1141	2.41E-05	0.0043644	glycine amidinotransferase (L-arginine:glycine amidinotransferase) (GATM)	GATM
Cluster-62068.15859	3.1076	2.41E-05	0.0043645	Ailuropoda melanoleuca uncharacterized LOC105235271 (LOC105235271), ncRNA	--
Cluster-62068.165476	3.1064	2.03E-05	0.0038347	chromosome 17, clone RP11-613C6	--
Cluster-62068.131637	3.1061	6.23E-05	0.0092356	Ovis canadensis canadensis isolate 43U chromosome 2 sequence	--
Cluster-62068.67553	3.1034	5.46E-05	0.0083261	ring finger protein 135 (RNF135)	SQSTM1
Cluster-62068.44616	3.1005	4.54E-05	0.0072387	proprotein convertase subtilisin/kexin type 1 (PCSK1)	PCSK1
Cluster-62068.66513	3.0976	4.85E-05	0.0076141	solute carrier family 26 (anion exchanger), member 4 (SLC26A4)	SLC26A4, PDS

Cluster-62068.47305	3.0963	5.72E-05	0.0086466	sperm flagellar 2 (SPEF2)	SPEF2
Cluster-62068.53740	3.0892	7.17E-05	0.010339	uncharacterized LOC101674124 (LOC101674124), ncRNA	--
Cluster-62068.103295	3.0882	6.87E-05	0.0099711	tafazzin (TAZ)	TAZ
Cluster-62068.151484	3.0882	2.24E-05	0.0041219	enolase superfamily member 1 (ENOSF1)	fucD
Cluster-62068.47949	3.0865	5.98E-05	0.0089243	WD repeat domain 60 (WDR60)	WDR60
Cluster-62068.105181	3.0856	3.46E-05	0.0058883	Felis catus solute carrier family 25 (mitochondrial carrier; ornithine transporter) member 15 (SLC25A15)	SLC25A2_15, ORNT
Cluster-62068.94757	3.0854	8.10E-13	1.82E-09	Odobenus rosmarus divergens collagen, type I, (COL1A1)	COL1A1
Cluster-62068.96949	3.0812	3.36E-05	0.0057587	folate receptor 1 (adult) (FOLR1)	FOLR
Cluster-62068.96981	3.0654	2.03E-05	0.0038386	actin, alpha, cardiac muscle 1 (ACTC1)	ACTC1
Cluster-62068.28614	3.0652	7.37E-05	0.010536	acyl-CoA dehydrogenase family, member 10 (ACAD10)	ACAD10
Cluster-62068.113337	3.0636	2.35E-05	0.0042782	laminin, gamma 2 (LAMC2)	LAMC2
Cluster-62068.93965	3.0619	2.12E-08	1.33E-05	thiosulfate sulfurtransferase (rhodanese) (TST)	TST, MPST, sseA
Cluster-62068.131591	3.055	7.30E-05	0.010485	tweety family member 1 (TTYH1)	TTYH1
Cluster-62068.35084	3.0548	8.17E-05	0.011391	zinc finger, DHHC-type containing 3 (ZDHHC3)	ZDHHC3
Cluster-62068.139526	3.0488	8.56E-05	0.011823	chromosome 17, clone CTD-2132N18	--
Cluster-62068.81069	3.0442	7.69E-09	5.68E-06	alpha-2-macroglobulin-like 1 (A2ML1), transcript variant X4	A2ML1
Cluster-62068.128336	3.0378	8.19E-05	0.011394	Bos taurus isolate 9198 myocyte enhancer factor 2B (MEF2B) gene, promoter region	RFXANK
Cluster-62068.24987	3.0313	9.49E-05	0.012761	mal, T-cell differentiation protein-like (MALL)	MALL
Cluster-62068.127386	3.0309	9.01E-05	0.012325	zinc finger, AN1-type domain 4 (ZFAND4)	ANUBL1
Cluster-62068.56654	3.0305	1.08E-05	0.0023245	Canis lupus familiaris tetraspanin 9 (TSPAN9), transcript variant X7	TSPAN9
Cluster-78916.0	3.0298	8.66E-05	0.011934	Ovis canadensis canadensis isolate 43U chromosome 4 sequence	--
Cluster-62068.51047	3.0235	3.46E-05	0.0058883	Ceratotherium simum simum UTX gene, intron 22	--
Cluster-62068.131405	3.0151	0.000103	0.013621	Neovison vison clone Mvi1905 microsatellite sequence	--

Cluster-62068.95239	3.0133	4.96E-05	0.0077126	Odobenus rosmarus divergens collagen alpha-1(II) chain-like (LOC101382725)	--
Cluster-62068.95003	3.0087	9.03E-07	0.0003178	Ovis canadensis canadensis isolate 43U chromosome 2 sequence	--
Cluster-62068.58023	3.0078	0.000115	0.014845	zinc finger protein 319 (ZNF319)	ZNF319
Cluster-62068.146307	3.0057	0.000103	0.013636	Ovis canadensis canadensis isolate 43U chromosome 19 sequence	--
Cluster-62068.18582	3.0009	2.99E-07	0.00012566	GINS complex subunit 4 (Sld5 homolog) (GINS4)	GINS4, SLD5
Cluster-62068.82476	3.0002	2.28E-10	2.62E-07	C1q and tumor necrosis factor related protein 6 (C1QTNF6)	C1QTNF6
Cluster-62068.60605	3	0.000112	0.014543	chromosome unknown open reading frame, human C16orf71 (LOC101691483), transcript variant X7	--
Cluster-62068.22408	2.9981	6.73E-05	0.0098333	syndecan binding protein (syntenin) 2 (SDCBP2)	SDCBP2
Cluster-62068.65719	2.9973	0.000109	0.014207	Rho guanine nucleotide exchange factor (GEF) 17 (ARHGEF17)	ARHGEF17
Cluster-60388.0	2.9967	5.32E-05	0.0081572	cartilage intermediate layer protein 2 (CILP2)	CILP2
Cluster-62068.121712	2.995	0.000122	0.015546	Leptonychotes weddellii vacuolar protein sorting 33 homolog B (yeast) (VPS33B)	VPS33B
Cluster-62068.27992	2.994	0.000125	0.015794	Ovis canadensis canadensis isolate 43U chromosome 12 sequence	--
Cluster-62068.50702	2.9927	0.000107	0.014003	armadillo repeat containing 6 (ARMC6), transcript variant X4	ARMC6
Cluster-62068.102523	2.9927	8.67E-07	0.00030812	Ursus maritimus archain 1 (ARCN1)	ARCN1
Cluster-62068.36557	2.9873	2.00E-05	0.0038049	phosphodiesterase 1C, calmodulin-dependent 70kDa (PDE1C), transcript variant X4	PDE1
Cluster-62068.79057	2.9869	1.84E-05	0.0035518	SH3-domain GRB2-like 3 (SH3GL3), misc_RNA	SH3GL
Cluster-62068.61558	2.9848	1.77E-05	0.0034578	ATPase, class V, type 10B (ATP10B)	ATP10B
Cluster-62068.30490	2.9843	1.60E-05	0.0031695	Src-like-adaptor (SLA), transcript variant X4	SLA
Cluster-62068.90444	2.9787	4.54E-06	0.0011732	histone H1.2 (LOC101676607)	H1_5
Cluster-62068.46454	2.977	0.000138	0.017053	protein tyrosine phosphatase, receptor type, R (PTPRR)	PTPRR
Cluster-62068.96001	2.9741	0.000142	0.017287	Ailuropoda melanoleuca KIAA1324 ortholog (KIAA1324)	KIAA1324

Cluster-62068.160427	2.9721	7.39E-05	0.010558	TRAF family member-associated NFKB activator (TANK), transcript variant X4	TANK
Cluster-62068.180279	2.9706	0.00015	0.018035	myosin VIIA (MYO7A)	MYO7
Cluster-62068.92429	2.9692	1.17E-05	0.002469	uncharacterized LOC106005866 (LOC106005866), ncRNA	--
Cluster-62068.155733	2.9676	0.000129	0.016258	Canis Familiaris chromosome 17, clone XX-365L15	RP-L33, MRPL33, rpmG
Cluster-62068.155698	2.9629	0.000127	0.016002	chromosome 11, clone RP1-59M18	--
Cluster-62068.72150	2.9626	0.000134	0.016694	solute carrier family 6 (neurotransmitter transporter), member 6 (SLC6A6)	SLC6A6S
Cluster-49238.0	2.9595	0.000147	0.017736	uncharacterized LOC106004415 (LOC106004415), ncRNA	--
Cluster-62068.157105	2.9579	5.78E-05	0.0087039	calcium/calmodulin-dependent protein kinase II gamma (CAMK2G)2	CAMK2
Cluster-62068.92430	2.9552	7.23E-05	0.010399	Canis lupus familiaris uncharacterized LOC102152191 (LOC102152191), misc_RNA	--
Cluster-62068.167148	2.9468	0.000152	0.018216	Ursus maritimus uncharacterized LOC103676250 (LOC103676250), ncRNA	--
Cluster-62068.47350	2.9448	2.30E-05	0.0042272	cholinergic receptor, nicotinic, alpha 3 (neuronal) (CHRNA3)	CHRNA3
Cluster-62068.63411	2.9404	0.000162	0.019213	Odobenus rosmarus divergens dishevelled associated activator of morphogenesis 1 (DAAM1)	DAAM
Cluster-62068.64128	2.94	4.52E-05	0.0072221	vesicle-associated membrane protein 4 (VAMP4)	VAMP4
Cluster-62068.163008	2.9374	0.000134	0.016716	solute carrier family 26 (anion exchanger), member 4 (SLC26A4)	SLC26A4, PDS
Cluster-62068.33359	2.9366	0.00016	0.01909	dihydrouridine synthase 2 (DUS2), transcript variant X6	DUS2
Cluster-62068.38804	2.935	0.000155	0.018476	ATPase type 13A1 (ATP13A1)	ATP13A1
Cluster-22933.0	2.9304	0.000175	0.020272	cholinergic receptor, nicotinic, alpha 3 (neuronal) (CHRNA3)	CHRNA3
Cluster-60834.1	2.9272	0.000136	0.016824	gamma-aminobutyric acid (GABA) A receptor, delta (GABRD)	GABRD
Cluster-62068.107270	2.925	0.000178	0.020567	solute carrier family 6 (neurotransmitter transporter), member 8 (SLC6A8)	SLC6A6S

Cluster-62068.98524	2.9241	3.62E-06	0.00097823	sema domain, seven thrombospondin repeats (type 1 and type 1-like), transmembrane domain (TM) and short cytoplasmic domain, (semaphorin) 5A (SEMA5A), transcript variant X8	SEMA5
Cluster-62068.96002	2.9239	5.24E-08	2.89E-05	Ursus maritimus KIAA1324 ortholog (KIAA1324)	KIAA1324
Cluster-62068.192409	2.9229	0.000166	0.019591	Canis lupus familiaris fragile histidine triad (FHIT), transcript variant 1	FHIT
Cluster-62068.29783	2.9221	3.78E-05	0.0062539	E74-like factor 5 (ets domain transcription factor) (ELF5)	ELF5
Cluster-62068.57528	2.92	0.000121	0.015419	uncharacterized LOC106003856 (LOC106003856), ncRNA	--
Cluster-62068.29782	2.9179	0.00013	0.01629	E74-like factor 5 (ets domain transcription factor) (ELF5)	ELF5
Cluster-62068.39319	2.9079	0.000179	0.020648	tumor necrosis factor (ligand) superfamily, member 18 (TNFSF18)	TNFSF18, GITRL
Cluster-62068.95753	2.9032	1.74E-10	2.12E-07	Leptonychotes weddellii collagen alpha-1(I) chain-like (LOC102742903)	--
Cluster-62068.82311	2.8991	0.000154	0.018394	laminin, gamma 2 (LAMC2)	LAMC2
Cluster-62068.113616	2.8984	0.000187	0.021314	intraflagellar transport 57 (IFT57)	ESRRBL1, HIPPI
Cluster-62068.87854	2.8983	0.000201	0.022499	Leptonychotes weddellii transketolase (TKT)	tktA, tktB
Cluster-62068.35051	2.896	0.000117	0.015028	ATPase, class V, type 10B (ATP10B)	ATP10B
Cluster-62068.129382	2.888	0.000198	0.022304	RNA binding motif protein 38 (RBM38)	RBM38
Cluster-62068.109337	2.8867	0.000204	0.022702	Odobenus rosmarus divergens C2 calcium-dependent domain containing 4B (C2CD4B)	C2CD4B
Cluster-62068.61498	2.886	0.000228	0.02467	transient receptor potential cation channel, subfamily M, member 5 (TRPM5), transcript variant X7	TRPM5
Cluster-62068.84995	2.8804	9.57E-06	0.002126	alpha-2-macroglobulin-like 1 (A2ML1), transcript variant X4	A2ML1
Cluster-62068.94496	2.8794	1.58E-06	0.00049438	uncharacterized LOC106006158 (LOC106006158), ncRNA	--
Cluster-62068.150046	2.8729	9.65E-05	0.012905	annexin A3 (ANXA3)	ANXA3
Cluster-62068.130986	2.8719	2.19E-06	0.00064739	Ovis canadensis canadensis isolate 43U chromosome 16 sequence	--
Cluster-62068.87272	2.8687	0.000253	0.026575	leukocyte cell derived chemotaxin 1 (LECT1)	LECT1

Cluster-62068.171459	2.8674	0.000234	0.025112	Leber congenital amaurosis 5-like (LCA5L), transcript variant X6	LCA5L
Cluster-62068.18707	2.8657	0.000226	0.024515	angiopoietin-like 3 (ANGPTL3)	ANGPTL3
Cluster-62068.138577	2.8649	9.65E-05	0.012905	propionyl CoA carboxylase, alpha polypeptide (PCCA)	PCCA, pccA
Cluster-62068.81909	2.8646	0.000259	0.026985	low density lipoprotein receptor-related protein 2 (LRP2)	LRP2
Cluster-62068.68956	2.8638	0.00011	0.014325	solute carrier family 15 (oligopeptide transporter), member 1 (SLC15A1)	SLC15A1, PEPT1
Cluster-39470.0	2.8636	0.000248	0.026267	chromosome 17, clone RP11-666A8	--
Cluster-62068.22983	2.8616	1.55E-07	7.29E-05	Ailuropoda melanoleuca coagulation factor II (thrombin) (F2)	F2
Cluster-62068.56013	2.8608	0.000247	0.026201	FYVE, RhoGEF and PH domain containing 3 (FGD3)	FGD3
Cluster-62068.88944	2.8508	0.000272	0.028057	Neovison vison clone LINE	--
Cluster-62068.146840	2.8494	0.000249	0.026366	ABI family, member 3 (ABI3)	ABI3
Cluster-62068.82402	2.846	7.80E-05	0.010995	uncharacterized LOC106003559 (LOC106003559), ncRNA	--
Cluster-62068.116713	2.8443	1.77E-10	2.14E-07	carboxypeptidase Z (CPZ)	CPZ
Cluster-62068.140464	2.8427	0.000104	0.013689	solute carrier family 4, sodium bicarbonate cotransporter, member 8 (SLC4A8), RefSeqGene on chromosome 12	SLC4A8
Cluster-62068.139558	2.84	0.000172	0.020131	regulating synaptic membrane exocytosis 3 (RIMS3)	RIMS3
Cluster-62068.8063	2.8397	0.000272	0.028043	genomic DNA, chromosome 11 clone:RP11-775D16	--
Cluster-62068.120348	2.8394	0.000265	0.027487	peptidase D (PEPD), RefSeqGene on chromosome 19	PEPD
Cluster-62068.74882	2.8389	0.00027	0.027875	Odobenus rosmarus divergens protein tyrosine phosphatase type IVA, member 1 (PTP4A1)	PTP4A
Cluster-62068.104318	2.8377	0.00032	0.031402	synaptotagmin IV (SYT4)	SYT4
Cluster-62068.90611	2.8371	1.93E-07	8.83E-05	Mustela vison clone IX3J3s1 genomic sequence	--
Cluster-62068.149110	2.8351	0.00021	0.023187	7-dehydrocholesterol reductase (DHCR7)	DHCR7
Cluster-62068.143453	2.8315	5.95E-05	0.0088883	TBC1 domain family, member 2 (TBC1D2), transcript variant X4	TBC1D2
Cluster-62068.164409	2.8235	0.000288	0.029051	histone deacetylase 3 (HDAC3)	HDAC3

Cluster-62068.96118	2.8233	3.45E-10	3.73E-07	Ailuropoda melanoleuca KIAA1324 ortholog (KIAA1324)	KIAA1324
Cluster-62068.172070	2.8232	0.0002	0.022401	uncharacterized LOC106006908 (LOC106006908), ncRNA	--
Cluster-62068.122367	2.8199	0.000175	0.020275	dedicator of cytokinesis 5 (DOCK5)	DOCK5
Cluster-62068.98388	2.8188	2.28E-05	0.0041866	nudix (nucleoside diphosphate linked moiety X)-type motif 5 (NUDT5), transcript variant X6	NUDT5
Cluster-62068.102545	2.8185	4.44E-06	0.0011493	solute carrier family 16, member 14 (SLC16A14)	SLC16A14
Cluster-62068.69865	2.8146	0.000351	0.033861	steroid-5-alpha-reductase, alpha polypeptide 1 (3-oxo-5 alpha-steroid delta 4-dehydrogenase alpha 1) (SRD5A1)	SRD5A1
Cluster-62068.158265	2.805	0.00014	0.017192	uncharacterized LOC101683862 (LOC101683862)4, ncRNA	--
Cluster-62068.143807	2.7996	1.15E-05	0.0024306	RAS-like, family 11, member B (RASL11B)	RASL11B
Cluster-62068.25078	2.7991	9.10E-09	6.49E-06	pregnancy up-regulated nonubiquitous CaM kinase (PNCK)	PNCK
Cluster-62068.114221	2.7929	3.41E-05	0.0058215	cyclin-dependent kinase-like 2 (CDC2-related kinase) (CDKL2)	CDKL2
Cluster-62068.94340	2.7889	0.000404	0.037512	epidermal growth factor (EGF)	EGF
Cluster-62068.119467	2.7828	9.08E-05	0.012397	signal-induced proliferation-associated 1 like 2 (SIPA1L2)	SIPA1L2, SPAL2
Cluster-67194.0	2.7824	0.000245	0.02609	Ursus maritimus cadherin 18, type 2 (CDH18)	CDH18
Cluster-62068.102660	2.7804	0.000375	0.035554	flotillin 1 (FLOT1)	FLOT
Cluster-62068.76801	2.777	0.000206	0.022906	Ursus maritimus translocase of outer mitochondrial membrane 70 homolog A (S. cerevisiae) (TOMM70A)	TOMM70A
Cluster-62068.125506	2.7755	2.63E-05	0.004686	nuclear receptor subfamily 4, group A, member 1 (NR4A1), transcript variant X4	NR4A1, HMR
Cluster-62068.101242	2.7738	3.11E-09	2.68E-06	KIAA1324 ortholog (KIAA1324), transcript variant X4	KIAA1324
Cluster-62068.12536	2.7722	0.000223	0.024225	Fanconi anemia, complementation group M (FANCM)	FANCM
Cluster-62068.157467	2.7663	0.000394	0.036698	Odobenus rosmarus divergens basic proline-rich protein (LOC105757347)	--
Cluster-62068.92973	2.7628	0.000414	0.037995	milk fat globule-EGF factor 8 protein (MFGE8)	MFGE8

Cluster-62068.152657	2.7616	0.000406	0.0376	coagulation factor X (F10)	F10
Cluster-62068.97764	2.7603	0.000448	0.040393	Rap guanine nucleotide exchange factor (GEF) 3 (RAPGEF3)	RAPGEF3, EPAC1
Cluster-62068.135123	2.7542	0.000177	0.020452	target of EGR1, member 1 (nuclear) (TOE1)	TOE1
Cluster-62068.85308	2.7534	0.000424	0.038553	Ailuropoda melanoleuca YTH domain containing 1 (YTHDC1)	YTHDC1
Cluster-62068.98128	2.7516	1.46E-07	6.92E-05	1-acylglycerol-3-phosphate O-acyltransferase 9 (AGPAT9)	GPAT3_4, AGPAT9, AGPAT6
Cluster-62068.19300	2.7491	0.000174	0.020215	Human DNA sequence from clone RP11-31E23 on chromosome 1q31.3-32.1	--
Cluster-61968.0	2.7447	0.000472	0.04184	bora, aurora kinase A activator (BORA)	BORA
Cluster-62068.146379	2.742	0.00012	0.015324	solute carrier family 24 (SLC24A2)	SLC24A2
Cluster-62068.101040	2.733	0.000261	0.027133	chromosome 16 clone RP11-413H22	--
Cluster-62068.79119	2.7283	1.75E-07	8.17E-05	Ovis canadensis canadensis isolate 43U chromosome 20 sequence	--
Cluster-62068.102191	2.7277	4.02E-08	2.32E-05	X-linked Kx blood group (XK)	XK
Cluster-62068.98127	2.7262	1.25E-07	6.12E-05	1-acylglycerol-3-phosphate O-acyltransferase 9 (AGPAT9)	AGPAT9
Cluster-62068.101252	2.726	5.94E-05	0.0088883	TBP-like 1 (TBPL1)	TBPL1
Cluster-62068.177044	2.7234	0.000365	0.034845	Ovis canadensis canadensis isolate 43U chromosome 1 sequence	--
Cluster-62068.175046	2.7151	0.000325	0.031728	troponin I type 1 (skeletal, slow) (TNNI1)	TNNI1
Cluster-62068.164091	2.7149	0.00054	0.046254	suppressor of variegation 3-9 homolog 2 (Drosophila) (SUV39H2)	SUV39H2
Cluster-62068.109663	2.7128	1.38E-05	0.002848	cyclin-dependent kinase-like 2 (CDC2-related kinase) (CDKL2)	CDKL
Cluster-62068.83341	2.7106	5.84E-07	0.00022188	Ovis canadensis canadensis isolate 43U chromosome 3 sequence	--
Cluster-62068.101731	2.7085	1.88E-07	8.72E-05	SUN domain containing ossification factor (SUCO), transcript variant X4, misc_RNA	SUCO
Cluster-62068.169218	2.7071	0.000586	0.049108	uncharacterized LOC106006266 (LOC106006266)	--
Cluster-62068.135865	2.7067	0.000587	0.049141	lipocalin 1 (LCN1)	LCN1

Cluster-62068.98833	2.7062	5.60E-07	0.0002141	C1q and tumor necrosis factor related protein 6 (C1QTNF6)	C1QTNF6
Cluster-62068.146922	2.7056	0.00032	0.031402	formin homology 2 domain containing 3 (FHOD3)	FHOD3
Cluster-62068.79104	2.6989	0.000569	0.048098	limb and CNS expressed 1 like (LIX1L)	LIX1L
Cluster-55624.0	2.6967	0.000596	0.049757	Pan troglodytes BAC clone CH251-679I17 from chromosome 15	--
Cluster-40312.0	2.6965	0.000304	0.03028	Canis familiaris chromosome 8, clone XX-152I15	--
Cluster-62068.83975	2.6944	3.67E-06	0.00098671	Ursus maritimus coatomer protein complex, subunit beta 2 (beta prime) (COPB2)	COPB2, SEC27
Cluster-62068.178761	2.6918	0.000161	0.019124	KIAA1614 ortholog (KIAA1614)	KIAA1614
Cluster-62068.15067	2.6901	0.000477	0.04213	Ovis canadensis canadensis isolate 43U chromosome 3 sequence	--
Cluster-62068.114400	2.6896	4.08E-05	0.0066692	Rhesus Macaque BAC CH250-276H13	--
Cluster-54352.0	2.6895	0.000152	0.018217	Canis lupus familiaris zinc finger protein 454 (ZNF454)	KRAB
Cluster-62068.139715	2.6869	9.30E-07	0.0003258	Odobenus rosmarus divergens PDZ domain containing 8 (PDZD8)	PDZD8
Cluster-22215.0	2.6849	5.80E-05	0.008723	uncharacterized LOC101686146 (LOC101686146), ncRNA	--
Cluster-26385.0	2.6823	0.000425	0.038672	Cercocebus atys regulator of G-protein signaling 3 (RGS3)	RGS3
Cluster-62068.80525	2.6804	3.09E-07	0.00012844	Canis familiaris, clone XX-228B21	--
Cluster-62068.135412	2.678	0.000317	0.031162	Odobenus rosmarus divergens Rap guanine nucleotide exchange factor (GEF) 4 (RAPGEF4)	RAPGEF4, EPAC2
Cluster-62068.90443	2.6708	0.000488	0.042986	histone H1.2 (LOC101676607)	H1_5
Cluster-62068.14645	2.6666	0.000503	0.043814	Ailuropoda melanoleuca clone gpbaab	--
Cluster-62068.136072	2.6666	0.000597	0.049757	Bos taurus uncharacterized LOC101905583 (LOC101905583), ncRNA	--
Cluster-62068.120539	2.6652	0.000168	0.019787	solute carrier family 4, sodium bicarbonate cotransporter, member 8 (SLC4A8)	SLC4A8
Cluster-62068.69420	2.6652	4.56E-05	0.00725	solute carrier family 12 (potassium/chloride transporter), member 7 (SLC12A7), transcript variant X4	SLC12A7, KCC4
Cluster-62068.61556	2.6624	0.000382	0.036011	ATPase, class V, type 10B (ATP10B)	ATP10B

Cluster-62068.99363	2.6602	5.37E-06	0.0013462	ornithine aminotransferase (OAT)	rocD, OAT
Cluster-62068.152726	2.6571	0.000226	0.024515	zinc finger protein 469 (ZNF469)	ZNF469
Cluster-62068.96613	2.6523	5.33E-05	0.0081572	Rhesus Macaque BAC CH250-276H13 () complete sequence	SIAT4C
Cluster-62068.82928	2.6496	0.000284	0.028802	Felis catus FLA extended class II, class II, class III, proximal and central class I region genomic sequence	--
Cluster-79398.0	2.649	0.000239	0.025472	cannabinoid receptor 1 (brain) (CNR1)	CNR1
Cluster-62068.30601	2.6489	0.000202	0.022625	Ovis canadensis canadensis isolate 43U chromosome 17 sequence	--
Cluster-62068.86756	2.6414	0.000468	0.04166	transmembrane protein 218 (TMEM218)	SLC37A1_2
Cluster-35572.0	2.6412	0.000215	0.023549	Human DNA sequence from clone RP11-336F23 on chromosome 1	--
Cluster-62068.73385	2.6327	0.000302	0.030139	BAC clone RP11-468A23 from 4	--
Cluster-62068.114799	2.631	5.49E-05	0.0083625	atypical chemokine receptor 4 (ACKR4)	CCRL1, CCR11
Cluster-62068.130037	2.6308	2.86E-07	0.00012069	Ailuropoda melanoleuca fucosyltransferase 10 (alpha (1,3) fucosyltransferase) (FUT10)	FUT10
Cluster-62068.161830	2.6289	0.000357	0.034314	Ovis canadensis canadensis isolate 43U chromosome 12 sequence	--
Cluster-62068.168792	2.6264	0.000358	0.034314	neuron navigator 3 (NAV3), transcript variant X4	NAV3
Cluster-62068.97113	2.6247	0.000408	0.03776	FXYD domain containing ion transport regulator 4 (FXYD4), transcript variant X4	FXYD4, CHIF
Cluster-62068.118666	2.6239	0.000352	0.033942	polymerase (RNA) III (DNA directed) polypeptide B (POLR3B)	RPC2, POLR3B
Cluster-62068.148210	2.6158	0.00033	0.032177	opioid binding protein/cell adhesion molecule-like (OPCML), transcript variant X9	OPCML
Cluster-62068.31066	2.6136	0.000175	0.020275	T-cell surface glycoprotein CD1a-like (LOC101689902)	CD1
Cluster-62068.94489	2.602	3.29E-06	0.00090819	antizyme inhibitor 1 (AZIN1), transcript variant 2	AZIN1
Cluster-62068.106988	2.5979	0.000297	0.029762	transmembrane protein 27 (TMEM27)	TMEM27
Cluster-62068.87375	2.597	3.76E-05	0.0062247	PDZ domain containing 1 (PDZK1)	PDZK1
Cluster-62068.80564	2.5931	0.000177	0.020488	Ailuropoda melanoleuca delta-like 1 (Drosophila) (DLL1)	DLL

Cluster-62068.115474	2.5895	0.000101	0.013409	transcription factor CP2-like 1 (TFCP2L1)	TFCP2
Cluster-62068.95848	2.5875	2.39E-05	0.0043406	CD320 molecule (CD320)	CD320
Cluster-62068.151037	2.5843	0.000358	0.034314	Ursus maritimus cysteine conjugate-beta lyase 2 (CCBL2), transcript variant X4	CCBL
Cluster-62068.107255	2.58	0.000221	0.02412	branched chain amino-acid transaminase 1, cytosolic (BCAT1)	E2.6.1.42, ilvE
Cluster-62068.85556	2.5738	5.38E-05	0.008218	transmembrane protein 27 (TMEM27)	TMEM27
Cluster-62068.96013	2.569	3.77E-07	0.00014996	Ovis canadensis canadensis isolate 43U chromosome 1 sequence	--
Cluster-62068.161832	2.5687	0.000544	0.046537	Ailuropoda melanoleuca uncharacterized LOC105239280 (LOC105239280), ncRNA	--
Cluster-62068.110767	2.5665	0.000122	0.01552	coiled-coil domain containing 15 (CCDC15), transcript variant X6	CCDC15
Cluster-62068.77347	2.566	3.91E-05	0.0064187	TBC1 domain family, member 19 (TBC1D19)	TBC1D19
Cluster-62068.126323	2.5642	0.000256	0.026831	Human DNA sequence from clone RP11-336F23 on chromosome 1	--
Cluster-62068.82852	2.5614	0.000307	0.030461	laminin, alpha 1 (LAMA1)	LAMA1_2
Cluster-62068.20827	2.5552	0.00049	0.043086	Pan troglodytes BAC clone CH251-696K14 from chromosome 11	--
Cluster-62068.90194	2.5549	1.45E-12	3.02E-09	alpha-2-macroglobulin-like 1 (A2ML1)	A2ML1
Cluster-62068.88240	2.5522	1.95E-05	0.0037219	ST3 beta-galactoside alpha-2,3-sialyltransferase 4 (ST3GAL4), transcript variant X6	SIAT4C
Cluster-62068.98468	2.5403	0.000468	0.041665	Canis lupus familiaris serine/arginine-rich splicing factor 4 (SRSF4)	SFRS4_5_6
Cluster-62068.97509	2.5338	0.00019	0.021611	Pig DNA sequence from clone CH242-113D8 on chromosome 7	--
Cluster-62068.68790	2.5311	3.16E-06	0.00088541	regulator of calcineurin 1 (RCAN1)	RCAN1, MCIP1
Cluster-62068.75215	2.5288	0.000374	0.035514	Ovis canadensis canadensis isolate 43U chromosome 6 sequence	--
Cluster-62068.80357	2.5279	4.04E-11	5.88E-08	flavin containing monooxygenase 2 (non-functional) (FMO2)	FMO
Cluster-62068.150670	2.5264	0.000343	0.033169	synaptotagmin-like 3 (SYTL3)	SYTL
Cluster-62068.99755	2.5241	1.24E-05	0.0025878	transcription factor CP2-like 1 (TFCP2L1)	TFCP2L1
Cluster-62068.143551	2.5208	7.92E-05	0.011109	Mandrillus leucophaeus uncharacterized LOC105531271 (LOC105531271),	--

				misc_RNA	
Cluster-62068.91641	2.5098	4.28E-06	0.0011174	1-acylglycerol-3-phosphate O-acyltransferase 9 (AGPAT9)	GPAT3_4, AGPAT9, AGPAT6
Cluster-62068.109550	2.5025	3.38E-05	0.0057908	ST3 beta-galactoside alpha-2,3-sialyltransferase 4 (ST3GAL4), transcript variant X6	SIAT4C
Cluster-62068.67899	2.4986	1.94E-07	8.83E-05	major facilitator superfamily domain containing 2A (MFSD2A)	MFSD2A
Cluster-62068.124438	2.4973	2.22E-07	9.82E-05	p21 protein (Cdc42/Rac)-activated kinase 3 (PAK3), RefSeqGene on chromosome X	PAK3
Cluster-62068.123209	2.4942	0.000593	0.049546	vasoactive intestinal peptide receptor 2 (VIPR2)	VIPR2
Cluster-62068.91677	2.4855	8.88E-07	0.00031329	Ursus maritimus flavin containing monooxygenase 2 (non-functional) (FMO2), transcript variant X4	FMO2
Cluster-62068.79054	2.4849	0.000104	0.013748	SH3-domain GRB2-like 3 (SH3GL3), transcript variant X4	SH3GL
Cluster-62068.66058	2.4758	3.72E-07	0.00014871	Odobenus rosmarus divergens solute carrier family 25 (mitochondrial carnitine/acylcarnitine carrier), member 29 (SLC25A29)	SLC25A29
Cluster-62068.88702	2.4736	5.04E-05	0.0078104	solute carrier family 16, member 14 (SLC16A14)	SLC16A14
Cluster-62068.70296	2.472	0.000295	0.029556	Canis familiaris, clone XX-134M17	--
Cluster-62068.67298	2.4694	0.00014	0.01719	NDRG family member 3 (NDRG3)	NDRG3
Cluster-62068.78547	2.4676	6.05E-07	0.00022932	activated leukocyte cell adhesion molecule (ALCAM), transcript variant X4	ALCAM
Cluster-62068.124082	2.4657	5.53E-09	4.38E-06	synovial sarcoma, X breakpoint 2 interacting protein (SSX2IP)	SSX2IP, ADIP
Cluster-62068.119476	2.4651	1.83E-05	0.0035486	SH3-domain GRB2-like 3 (SH3GL3), misc_RNA	SH3GL3
Cluster-62068.98984	2.4633	2.20E-06	0.00064955	flavin containing monooxygenase 2 (non-functional) (FMO2)	FMO
Cluster-62068.97284	2.4607	0.000415	0.038043	megalencephalic leukoencephalopathy with subcortical cysts 1 (MLC1)	--
Cluster-62068.113928	2.4593	0.000172	0.020073	fibroblast growth factor receptor 4 (FGFR4)	FGFR4
Cluster-62068.128798	2.4584	1.38E-06	0.00044523	carboxypeptidase Z (CPZ)	CPZ

Cluster-62068.153242	2.4562	0.000418	0.038181	collagen triple helix repeat containing 1 (CTHRC1)	CTHRC1
Cluster-62068.84286	2.4561	5.52E-05	0.0083817	Canis Familiaris chromosome 27, clone XX-53N21	--
Cluster-62068.111614	2.4471	5.78E-05	0.0087039	noggin (NOG)	NOG
Cluster-62068.62540	2.4457	7.57E-07	0.00027461	Ailuropoda melanoleuca transmembrane protease, serine 2 (TMPRSS2)	TMPRSS2
Cluster-62068.106838	2.4427	3.75E-06	0.0010058	KIAA1324 ortholog (KIAA1324), transcript variant X4	KIAA1324
Cluster-74011.0	2.4376	0.000553	0.047	Odobenus rosmarus divergens v-myc avian myelocytomatosis viral oncogene neuroblastoma derived homolog (MYCN)	NMYC, MYCN
Cluster-62068.91676	2.4371	4.54E-05	0.0072387	Ursus maritimus flavin containing monooxygenase 2 (non-functional) (FMO2), transcript variant X4	FMO2
Cluster-18608.0	2.4369	0.000459	0.041087	interleukin 1, alpha (IL1A)	IL1A
Cluster-62068.43614	2.4299	0.00015	0.017976	Pig DNA sequence from clone CH242-240D13 on chromosome 1	--
Cluster-62068.118383	2.4218	4.66E-05	0.0073499	SH3-domain GRB2-like 3 (SH3GL3)	SH3GL
Cluster-62068.90993	2.4199	3.40E-05	0.0058172	Ovis canadensis canadensis isolate 43U chromosome 22 sequence	--
Cluster-62068.95030	2.4163	9.05E-05	0.012362	uncharacterized LOC106005866 (LOC106005866), ncRNA	--
Cluster-62068.66886	2.411	2.22E-05	0.004097	sema domain, seven thrombospondin repeats (type 1 and type 1-like), transmembrane domain (TM) and short cytoplasmic domain, (semaphorin) 5A (SEMA5A), transcript variant X8	SEMA5
Cluster-62068.84736	2.4106	2.92E-08	1.74E-05	plastin 1 (PLS1)	PLS1
Cluster-62068.81599	2.4105	1.13E-07	5.71E-05	cadherin 4, type 1, R-cadherin (retinal) (CDH4)	CDH4
Cluster-62068.86350	2.4104	0.000337	0.032742	Canis lupus familiaris hydrogen voltage-gated channel 1 (HVCN1)	HVCN1
Cluster-62068.131847	2.407	2.12E-05	0.0039638	serpin peptidase inhibitor, clade B (ovalbumin), member 2 (SERPINB2)	SERPINB
Cluster-62068.131888	2.4057	5.90E-08	3.24E-05	cadherin 4, type 1, R-cadherin (retinal) (CDH4)	CDH4
Cluster-62068.81648	2.4023	6.70E-09	5.08E-06	Odobenus rosmarus divergens ADAM metallopeptidase with thrombospondin type 1 motif, 9 (ADAMTS9)	ADAMTS9
Cluster-62068.180705	2.3976	0.000418	0.038218	Pongo abelii BAC clone CH276-169E9 from chromosome 5	--

Cluster-62068.96975	2.3903	8.13E-05	0.011342	Ursus maritimus KIAA1324 ortholog (KIAA1324)	KIAA1324
Cluster-62068.80428	2.3851	0.000187	0.021314	dishevelled-binding antagonist of beta-catenin 2 (DACT2)	DACT2
Cluster-62068.133033	2.3827	1.87E-06	0.00056555	D-amino-acid oxidase (DAO)	DAO, aao
Cluster-62068.100814	2.3799	0.000245	0.026097	genomic DNA, chromosome 8q23, clone: KB1254G8	--
Cluster-62068.22702	2.3788	3.06E-05	0.0053088	Canis familiaris, clone XX-459P12	--
Cluster-62068.90360	2.3743	9.16E-06	0.0020663	inositol monophosphatase domain containing 1 (IMPAD1)	--
Cluster-62068.107007	2.374	0.000267	0.027645	carboxypeptidase X (M14 family), member 2 (CPXM2)	CPXM2
Cluster-62068.120318	2.3708	9.21E-05	0.012504	Apteryx australis mantelli genome assembly AptMant0, scaffold scaffold5468	--
Cluster-62068.76103	2.3693	6.34E-05	0.0093786	mannosyl (alpha-1,3-)-glycoprotein beta-1,4-N-acetylglucosaminyltransferase, isozyme A (MGAT4A)	--
Cluster-62068.106987	2.361	9.74E-05	0.012991	transmembrane protein 27 (TMEM27), transcript variant X4	TMEM27
Cluster-62068.94216	2.3567	0.000108	0.014166	CD320 molecule (CD320)	CD320
Cluster-62068.142158	2.3567	0.000161	0.019171	nascent polypeptide-associated complex subunit alpha, muscle-specific form (LOC101682205)	--
Cluster-62068.124744	2.3566	0.000137	0.016907	cytidine deaminase (CDA)	cdd, CDA
Cluster-62068.20054	2.3427	8.58E-05	0.011843	chromosome 1 clone RP4-771M4	--
Cluster-62068.122412	2.3413	0.000236	0.025261	mannosyl (alpha-1,3-)-glycoprotein beta-1,4-N-acetylglucosaminyltransferase, isozyme A (MGAT4A)	--
Cluster-62068.124668	2.336	0.00017	0.019938	cytidine deaminase (CDA)	cdd, CDA
Cluster-62068.165904	2.3327	0.000338	0.032779	lipopolysaccharide-binding protein-like (LOC101690223)	LBP
Cluster-62068.124081	2.3279	1.07E-22	1.53E-18	synovial sarcoma, X breakpoint 2 interacting protein (SSX2IP)	SSX2IP, ADIP
Cluster-62068.93953	2.3257	2.59E-08	1.57E-05	collagen, type I, alpha 1 (COL1A1)	COL1AS
Cluster-62068.125024	2.3247	9.31E-09	6.54E-06	K(lysine) acetyltransferase 2A (KAT2A)	PCAF, KAT2, GCN5
Cluster-62068.167735	2.3196	0.000361	0.034593	BAC clone RP11-795C1 from 2	--

Cluster-62068.138482	2.3174	0.000405	0.03757	ADAM metallopeptidase with thrombospondin type 1 motif, 9 (ADAMTS9)	ADAMTS9
Cluster-62068.95613	2.313	8.39E-05	0.011618	transcription factor CP2-like 1 (TFCP2L1)	TFCP2
Cluster-62068.71214	2.3093	3.76E-07	0.00014996	FK506 binding protein 5 (FKBP5)	FKBP4_5
Cluster-62068.84885	2.3061	6.56E-06	0.0015762	Ailuropoda melanoleuca KIAA1324 ortholog (KIAA1324)	KIAA1324
Cluster-62068.93665	2.302	0.000109	0.014203	nudix (nucleoside diphosphate linked moiety X)-type motif 5 (NUDT5), transcript variant X4	NUDT5
Cluster-62068.132097	2.2992	3.62E-05	0.0060774	six transmembrane epithelial antigen of the prostate 1 (STEAP1)	STEAP1
Cluster-62068.105880	2.2957	1.15E-05	0.0024366	TBP-like 1 (TBPL1)	TBP, tbp
Cluster-62068.76470	2.2827	0.000374	0.035514	cAMP responsive element modulator (CREM)	CREM
Cluster-62068.96367	2.2796	6.74E-05	0.0098344	teneurin transmembrane protein 4 (TENM4)	TENM4
Cluster-62068.92725	2.2763	0.000336	0.032634	solute carrier family 6 (amino acid transporter), member 14 (SLC6A14)	SLC6A5S
Cluster-62068.80121	2.2724	0.000377	0.035665	like-glycosyltransferase (LARGE)	LARGE
Cluster-62068.88961	2.2719	1.68E-07	7.89E-05	Canis lupus familiaris plastin 1 (PLS1)	PLS1
Cluster-62068.99314	2.2703	0.000438	0.039603	Microcebus murinus cyclin G1 (CCNG1)	CCNG1
Cluster-62068.106871	2.2697	2.58E-08	1.56E-05	leucine rich repeat containing 3 (LRRC3)	LRRC3
Cluster-62068.146731	2.2688	0.000548	0.046685	Ovis canadensis canadensis isolate 43U chromosome 8 sequence	--
Cluster-62068.93129	2.2616	0.000264	0.027449	solute carrier family 16, member 14 (SLC16A14)	SLC16A14
Cluster-62068.89125	2.2564	1.27E-05	0.002648	methionine adenosyltransferase II, beta (MAT2B)	metK
Cluster-62068.70438	2.2515	2.13E-10	2.49E-07	heparan sulfate 6-O-sulfotransferase 2 (HS6ST2)	HS6ST2
Cluster-62068.92631	2.2504	6.46E-09	4.92E-06	Macaca nemestrina serine/arginine-rich splicing factor 7 (SRSF7), misc_RNA	GEMIN6, SIP2
Cluster-62068.81639	2.2496	4.68E-08	2.62E-05	ADAM metallopeptidase with thrombospondin type 1 motif, 9 (ADAMTS9)	ADAMTS9
Cluster-62068.137277	2.2491	4.31E-05	0.0069572	cell growth regulator with EF-hand domain 1 (CGREF1)	CGREF1
Cluster-62068.87001	2.2454	4.39E-05	0.0070799	Odobenus rosmarus divergens procollagen C-endopeptidase enhancer (PCOLCE)	--
Cluster-62068.129241	2.2437	0.000372	0.035445	uncharacterized LOC106006958 (LOC106006958), ncRNA	--

Cluster-62068.72796	2.2423	1.63E-05	0.0032095	SH3 and cysteine rich domain (STAC)	STAC
Cluster-62068.97641	2.2385	8.34E-05	0.011561	transmembrane protein 161A (TMEM161A)	TMEM161A
Cluster-62068.109051	2.2371	2.06E-09	1.83E-06	insulin-like growth factor 1 (somatomedin C) (IGF1)	IGF1
Cluster-62068.89656	2.2351	0.000169	0.0199	vascular endothelial growth factor A (VEGFA)	VEGFA
Cluster-62068.90765	2.2322	8.55E-06	0.0019633	proline rich membrane anchor 1 (PRIMA1)	SEPT3_9_12
Cluster-62068.91507	2.2229	0.000114	0.014692	Sus scrofa vascular endothelial growth factor A-like (LOC100737169)	VEGFA
Cluster-62068.69015	2.2223	7.88E-05	0.011091	flavin containing monooxygenase 2 (non-functional) (FMO2)	FMO
Cluster-62068.99667	2.2152	2.38E-07	0.00010342	collagen, type I, alpha 1 (COL1A1)	COL1AS
Cluster-62068.50776	2.2127	0.000105	0.013756	Leopardus geoffroyi isolate OGE-136949 SINE Can-SINE	--
Cluster-62068.95562	2.2078	7.12E-09	5.34E-06	collagen, type I, alpha 1 (COL1A1)	COL1AS
Cluster-62068.112734	2.2041	0.000271	0.027975	HNF1 homeobox A (HNF1A)	HNF1A
Cluster-62068.78156	2.2038	0.000571	0.04816	uncharacterized LOC106005866 (LOC106005866), ncRNA	--
Cluster-62068.43929	2.2034	6.56E-05	0.0096318	Ovis canadensis canadensis isolate 43U chromosome 3 sequence	--
Cluster-62068.111434	2.2033	3.85E-05	0.0063477	carboxypeptidase X (M14 family), member 1 (CPXM1)	CPXM1
Cluster-62068.107138	2.2003	0.000145	0.017595	V-set and immunoglobulin domain containing 10 (VSIG10)	TCF1, HNF1A
Cluster-62068.46432	2.1888	1.64E-05	0.0032242	msh homeobox 2 (MSX2)	MSX
Cluster-62068.95175	2.187	1.85E-06	0.00056079	1-acylglycerol-3-phosphate O-acyltransferase 9 (AGPAT9)	GPAT3_4, AGPAT9, AGPAT6
Cluster-62068.87339	2.1803	0.000331	0.032229	vascular endothelial growth factor A (VEGFA), RefSeqGene on chromosome 6	VEGFA
Cluster-62068.81524	2.1715	0.000598	0.049811	pyrroline-5-carboxylate reductase 1 (PYCR1)	PYCR1
Cluster-62068.118465	2.1663	6.95E-06	0.0016588	cystathionine gamma-lyase (CTH)	CTH
Cluster-62068.101676	2.1644	0.000423	0.038553	regulator of G-protein signaling 3 (RGS3), transcript variant X7	RGS3
Cluster-62068.92579	2.1617	0.000299	0.02993	transcription factor CP2-like 1 (TFCP2L1)	TFCP2

Cluster-62068.122417	2.1617	0.00026	0.027133	mannosyl (alpha-1,3-)-glycoprotein beta-1,4-N-acetylglucosaminyltransferase, isozyme A (MGAT4A)	MGAT4A_B
Cluster-62068.142126	2.1517	0.000445	0.040123	chromosome 17, clone RP11-314A20	--
Cluster-62068.142160	2.1516	0.000105	0.013766	Ailuropoda melanoleuca uncharacterized LOC100483315 (LOC100483315), ncRNA	--
Cluster-62068.36988	2.1444	0.000122	0.015467	steroid 17-alpha-hydroxylase/17,20 lyase (LOC101671806)	CYP17A
Cluster-62068.110058	2.1405	1.58E-05	0.0031423	Callithrix jacchus uncharacterized LOC103791965 (LOC103791965), ncRNA	--
Cluster-62068.113743	2.1382	1.92E-07	8.83E-05	glutamine-rich 1 (QRICH1)	QRICH1
Cluster-62068.102698	2.1349	2.73E-05	0.0048152	Bos taurus ornithine decarboxylase (ODC) gene, complete cds	ODC1, speC, speF
Cluster-62068.72261	2.1339	0.000308	0.030531	Odobenus rosmarus divergens complement component (3b/4b) receptor 1-like (CR1L)	CD46, MCP
Cluster-61909.0	2.1316	0.000125	0.015865	Ursus maritimus testis expressed 33 (TEX33)	TEX33
Cluster-62068.95616	2.1284	0.000551	0.046853	transcription factor CP2-like 1 (TFCP2L1)	TFCP2
Cluster-62068.99976	2.1271	2.68E-05	0.0047591	sel-1 suppressor of lin-12-like 3 (SEL1L3)	SEL1L3
Cluster-62068.103151	2.1253	0.000412	0.037912	myosin VA (heavy chain 12, myoxin) (MYO5A)	MYO5
Cluster-62068.84994	2.113	1.52E-07	7.20E-05	alpha-2-macroglobulin-like 1 (A2ML1)	A2ML1
Cluster-62068.120902	2.1058	2.84E-05	0.0049653	exportin 1 (XPO1)	XPO1
Cluster-62068.93996	2.1048	0.000358	0.034314	transmembrane protein 189 (LOC101670792)	UBE2V
Cluster-62068.61193	2.1029	1.59E-06	0.00049493	Mustela vison clone XI3L13 microsatellite Mvi4068 sequence	--
Cluster-62068.118467	2.1016	0.00045	0.04049	X-linked Kx blood group (XK)	XK
Cluster-62068.89072	2.1008	0.000271	0.027975	Canis lupus familiaris X-linked Kx blood group (McLeod syndrome) (XK)	XK
Cluster-62068.55523	2.1003	0.000132	0.016487	sarcosine dehydrogenase (SARDH)	SARDH
Cluster-62068.84005	2.0984	9.56E-06	0.002126	uncharacterized LOC106007259 (LOC106007259)	--
Cluster-62068.88740	2.0971	8.60E-05	0.011862	dual specificity phosphatase 14 (DUSP14)	K14165
Cluster-62068.92321	2.0957	0.000227	0.02454	polyhomeotic homolog 1 (Drosophila) (PHC1)	PHC1, EDR1

Cluster-62068.165219	2.0938	1.14E-05	0.0024149	vav 2 guanine nucleotide exchange factor (VAV2)	VAV2
Cluster-62068.106345	2.0871	4.11E-05	0.0067038	neuronal cell adhesion molecule (NRCAM)	NRCAM
Cluster-62068.104095	2.0826	0.000589	0.049264	PDX1 C-terminal inhibiting factor 1 (PCIF1)	PCIF1
Cluster-62068.98003	2.0815	1.00E-09	9.70E-07	insulin-like growth factor 1 (somatomedin C) (IGF1)	IGF1
Cluster-62068.150815	2.0785	1.38E-05	0.0028418	ankyrin repeat domain 34B (ANKRD34B)	ANKRD34B
Cluster-62068.75393	2.0748	0.000474	0.041928	Canis lupus familiaris salt-inducible kinase 1 (SIK1)	SIK1
Cluster-62068.92389	2.0743	0.000258	0.026966	serum/glucocorticoid regulated kinase 1 (SGK1)	SGK1
Cluster-62068.82784	2.0714	2.14E-06	0.00063886	catenin (cadherin-associated protein), alpha-like 1 (CTNNAL1)	CTNNAL1
Cluster-62068.86084	2.0607	6.24E-05	0.0092356	catenin (cadherin-associated protein), alpha-like 1 (CTNNAL1)	--
Cluster-62068.87940	2.0576	1.47E-06	0.00046644	fibromodulin (FMOD)	FMOD
Cluster-62068.102098	2.0572	9.74E-05	0.012991	laminin, alpha 1 (LAMA1)	LAMA1_2
Cluster-62068.62748	2.0562	7.69E-05	0.010851	phosphatase, orphan 2 (PHOSPHO2)	PHOSPHO2
Cluster-62068.50864	2.0561	0.000314	0.030941	KIAA1211 ortholog (KIAA1211)	KIAA1211
Cluster-62068.93024	2.053	0.000478	0.042158	deiodinase, iodothyronine (DIO3)	DIO3
Cluster-62068.118310	2.0529	2.63E-05	0.004686	SRSF protein kinase 2 (SRPK2)	SRPK2
Cluster-62068.89653	2.0483	0.000481	0.0424	Panthera tigris altaica vascular endothelial growth factor A (VEGFA)	VEGFA
Cluster-62068.103530	2.0464	3.20E-05	0.0055275	ornithine decarboxylase 1 (ODC1)	E4.1.1.17, ODC1, speC, speF
Cluster-62068.144780	2.0374	0.000301	0.030037	Ailuropoda melanoleuca WDYHV motif containing 1 (WDYHV1)	WDYHV1
Cluster-62068.131713	2.0358	0.000411	0.037912	PCI domain containing 2 (PCID2)	PCID2
Cluster-62068.86974	2.0344	1.73E-06	0.00052898	cAMP responsive element modulator (CREM)	CREM
Cluster-62068.66789	2.0331	0.000286	0.028913	phosphatase and actin regulator 4 (PHACTR4)	PHACTR4
Cluster-62068.101584	2.0282	1.58E-06	0.00049438	Ailuropoda melanoleuca histone H2B type 2-F (LOC105234948)	--
Cluster-62068.55131	2.0157	0.000496	0.043501	angiotensin I converting enzyme 2 (ACE2)	ACEH, ACE2
Cluster-62068.150902	2.0154	2.15E-05	0.0039976	actin-like 7B (ACTL7B)	ACTL7B

Cluster-62068.52176	2.0121	1.08E-05	0.0023245	cell adhesion molecule L1-like (CHL1)8	CHL1
Cluster-62068.78445	2.0098	0.000546	0.046578	Ursus maritimus tetraspanin 1 (TSPAN1)	TSPAN1
Cluster-62068.57152	2.0095	0.000515	0.044647	transmembrane protein 2 (TMEM2)	TMEM2
Cluster-62068.129189	2.0084	0.000205	0.02278	beaded filament structural protein 1, filensin (BFSP1)	BFSP1
Cluster-62068.97471	2.0049	0.000472	0.04184	uncharacterized LOC106005600 (LOC106005600)	--
Cluster-62068.100793	2.0039	4.04E-05	0.0066163	death effector domain containing 2 (DEDD2)	DEDD2
Cluster-62068.50231	2.0023	4.66E-05	0.0073499	BAC RP11-267D19	--
Cluster-62068.152128	2.0022	1.25E-05	0.0025976	ral guanine nucleotide dissociation stimulator-like 3 (RGL3)	RGL3
Cluster-62068.93630	2.0011	0.000166	0.019591	small nuclear RNA activating complex(SNAPC4)	SNAPC4
Cluster-62068.67908	2.0003	0.000113	0.014574	major facilitator superfamily domain containing 2A (MFSD2A)	MFSD2A
Cluster-62068.129311	1.9988	0.000135	0.01675	uncharacterized LOC101681318 (LOC101681318)	--
Cluster-62068.86988	1.9987	9.98E-06	0.0021878	solute carrier family 18 (vesicular monoamine transporter), member 2 (SLC18A2)	SLC18A1_2, VMAT
Cluster-62068.7808	1.9984	7.58E-05	0.010758	perilipin 5 (PLIN5)	PLIN5
Cluster-62068.172708	1.9911	0.000249	0.02633	Ailuropoda melanoleuca importin 9 (IPO9)	IPO9
Cluster-62068.67905	1.991	7.33E-05	0.010513	major facilitator superfamily domain containing 2A (MFSD2A)	MFSD2A
Cluster-62068.66760	1.9909	1.16E-05	0.0024563	sorbitol dehydrogenase (SORD)	SORD, gutB
Cluster-62068.130560	1.9895	2.32E-05	0.0042359	fatty acid desaturase 1 (FADS1)	FADS1
Cluster-62068.93317	1.9892	0.000137	0.016944	PH domain and leucine rich repeat protein phosphatase 1 (PHLPP1)	PHLPP
Cluster-62068.49033	1.9889	0.000379	0.035812	Ovis canadensis canadensis isolate 43U chromosome 1 sequence	--
Cluster-62068.102064	1.9735	0.000318	0.031225	ornithine decarboxylase 1 (ODC1)	ODC1, speC, speF
Cluster-62068.97183	1.9711	0.000528	0.045482	delta(4)-desaturase, sphingolipid 2 (DEGS2)	DEGS2
Cluster-62068.96130	1.9699	3.32E-06	0.00091267	collagen, type I, alpha 1 (COL1A1)	COL1A1
Cluster-62068.78477	1.9685	7.10E-07	0.00026227	Odobenus rosmarus divergens plastin 1 (PLS1)	PLS1
Cluster-62068.96213	1.9658	8.33E-06	0.001925	uncharacterized LOC106006467 (LOC106006467)	--

Cluster-62068.90488	1.965	0.000555	0.047071	EPH receptor B6 (EPHB6)	EPHB6
Cluster-62068.148149	1.962	7.88E-05	0.011091	BAC clone RP11-707F2 from 4	--
Cluster-62068.116556	1.9562	5.93E-05	0.0088776	TBC1 domain family (TBC1D19)	TBC1D19
Cluster-62068.93541	1.953	1.24E-12	2.62E-09	Ailuropoda melanoleuca laminin (LAMC3)	LAMC3
Cluster-62068.131399	1.952	7.94E-05	0.011117	colony stimulating factor 2 receptor, beta, low-affinity (granulocyte-macrophage) (CSF2RB)	CSF2RB, IL3RB
Cluster-62068.94276	1.9462	1.14E-06	0.00038622	laminin, gamma 3 (LAMC3)	LAMC3
Cluster-62068.98385	1.9449	0.000471	0.041825	nudix (nucleoside diphosphate linked moiety X)-type motif 5 (NUDT5)	NUDT5
Cluster-62068.154153	1.9419	0.000521	0.045088	Human DNA sequence from clone RP11-323H21 on chromosome 9q34.1-34.3	--
Cluster-62068.87156	1.9353	1.91E-07	8.83E-05	Bos taurus ornithine decarboxylase (ODC) gene	ODC
Cluster-62068.71991	1.9349	3.35E-05	0.0057533	TPA: long non-coding RNA OTTHUMT00000473295.1 (RP11-129M16.4 gene)	--
Cluster-62068.132690	1.9312	5.54E-05	0.0084114	WD repeat domain 11 (WDR11)	WDR11
Cluster-62068.90679	1.9296	7.84E-07	0.00028287	alpha-2-macroglobulin-like 1 (A2ML1)	A2ML1
Cluster-62068.78324	1.9255	0.000246	0.026112	Canis familiaris chromosome 20	--
Cluster-62068.84337	1.9253	0.000229	0.024693	laminin, alpha 1 (LAMA1)	LAMA1_2
Cluster-62068.94621	1.9248	0.000382	0.036011	Odobenus rosmarus divergens ST3 beta-galactoside alpha-2,3-sialyltransferase 4 (ST3GAL4)	ST3GAL4
Cluster-62068.35882	1.9224	0.000238	0.025429	UTP6, small subunit (SSU) processome component(UTP6)	UTP6
Cluster-62068.71296	1.921	0.000362	0.034684	Ovis canadensis canadensis isolate 43U chromosome 2 sequence	--
Cluster-62068.18034	1.9201	5.96E-05	0.0088967	chromosome 19, cosmids R31158, R31874, and R28125	--
Cluster-62068.98373	1.9144	1.07E-05	0.0023126	Mustela vison clone VII6J9 genomic sequence	--
Cluster-62068.86989	1.9142	0.000253	0.026589	solute carrier family 18 (vesicular monoamine transporter), member 2 (SLC18A2)	SLC18A2

Cluster-62068.113705	1.9129	1.48E-05	0.0029916	Sus scrofa mRNA, clone:ITT010099D03, expressed in small intestine	--
Cluster-62068.80991	1.9128	0.000503	0.043814	solute carrier family 23 (ascorbic acid transporter), member 1 (SLC23A1)	SLC23A1_2, SVCT1_2
Cluster-62068.103261	1.9126	0.000138	0.016987	nudix (nucleoside diphosphate linked moiety X)-type motif 5 (NUDT5)	NUDT5
Cluster-62068.88788	1.9123	0.000171	0.019968	Ailuropoda melanoleuca cyclin-dependent kinase 12 (CDK12)	CDK12
Cluster-62068.161987	1.911	4.41E-06	0.0011449	cell adhesion molecule L1-like (CHL1)8	CHL1
Cluster-62068.94419	1.909	0.000412	0.037912	Canis familiaris chromosome X, clone XX-146G22	--
Cluster-62068.49806	1.9	0.000376	0.035655	Odobenus rosmarus divergens PARK2 co-regulated (PACRG)	PACRG
Cluster-62068.104170	1.8968	0.000142	0.017274	endogenous retrovirus group PABLB member 1 Env polyprotein-like (LOC106003903)	--
Cluster-62068.122281	1.891	8.81E-05	0.012088	Pig DNA sequence from clone CH242-421P6 on chromosome X	--
Cluster-62068.116216	1.8892	6.01E-06	0.0014647	insulin-like growth factor 1 (IGF1)	IGF1
Cluster-62068.92142	1.8852	0.000528	0.045482	FOSMID clone ABC9-43898400I17 from chromosome 9	--
Cluster-62068.86435	1.8782	5.85E-06	0.0014395	carboxypeptidase N, polypeptide 1 (CPN1)	CPN1
Cluster-62068.96375	1.8764	6.45E-07	0.00024256	methylenetetrahydrofolate dehydrogenase (NADP+ dependent) 2, methenyltetrahydrofolate cyclohydrolase (MTHFD2)	MTHFD2
Cluster-62068.136646	1.8759	0.000473	0.041904	gamma-glutamyltransferase 1 (GGT1)	GGT1
Cluster-62068.99920	1.8738	0.000391	0.036655	histone H2B type 1 (LOC101671838)	--
Cluster-62068.108356	1.8714	1.32E-10	1.67E-07	Mustela vison clone XI3L13 microsatellite Mvi4068 sequence	--
Cluster-62068.95533	1.8642	2.19E-05	0.00405	secreted protein, acidic, cysteine-rich (osteonectin) (SPARC)	SPARC
Cluster-62068.119801	1.852	0.000385	0.03624	Leptonychotes weddellii lysine (K)-specific demethylase 1A (KDM1A)	KDM1A, AOF2, LSD1
Cluster-62068.67676	1.8502	1.97E-05	0.0037428	phosphoserine phosphatase (PSPH)	PSPH
Cluster-62068.106127	1.8459	8.69E-05	0.01196	C-type lectin domain family 11, member A (CLEC11A)	CLEC11A
Cluster-62068.85626	1.8459	0.000442	0.039935	chondroadherin (CHAD)	CHAD

Cluster-62068.134390	1.8431	4.47E-05	0.007168	flavin containing monooxygenase 2 (non-functional) (FMO2)	FMO
Cluster-62068.83129	1.8401	0.000343	0.033169	Odobenus rosmarus divergens laminin, gamma 3 (LAMC3)	LAMC3
Cluster-62068.98134	1.8387	4.89E-05	0.0076412	frizzled class receptor 1 (FZD1)	FZD1_7, fz
Cluster-62068.78319	1.8362	0.00017	0.019938	MARVEL domain containing 3 (MARVELD3)	MARVELD3
Cluster-62068.140928	1.835	3.33E-07	0.00013662	platelet-activating factor acetylhydrolase 2 (PAFAH2)	PAFAH
Cluster-62068.96824	1.8343	8.75E-06	0.0019901	secreted protein, acidic, cysteine-rich (osteonectin) (SPARC)	--
Cluster-62068.84735	1.8339	3.34E-06	0.00091744	plastin 1 (PLS1)	PLS1
Cluster-62068.124083	1.8251	0.000193	0.021809	synovial sarcoma, X breakpoint 2 interacting protein (SSX2IP)	SSX2IP, ADIP
Cluster-62068.107231	1.8245	2.00E-05	0.0038028	ADP-ribosylation factor-like 2 binding protein (ARL2BP)	ARL2BP, BART
Cluster-62068.140871	1.8183	4.19E-05	0.0068194	alpha-2-macroglobulin-like 1 (A2ML1)	A2ML1
Cluster-62068.128641	1.8167	5.51E-06	0.0013717	insulin-like growth factor 1 (IGF1)	IGF1
Cluster-62068.113665	1.8135	7.61E-05	0.010778	uncharacterized LOC101681516	--
Cluster-62068.136774	1.8084	0.000485	0.042734	Canis Familiaris chromosome 31	PSMG1, DSCR2, PAC1
Cluster-62068.70688	1.8048	1.64E-05	0.0032331	xenotropic and polytropic retrovirus receptor 1 (XPR1)	XPR1
Cluster-62068.75151	1.803	0.000347	0.03355	solute carrier family 16(SLC16A6)	SLC16A6
Cluster-62068.107886	1.8009	5.19E-05	0.0079888	potassium channel tetramerization domain containing 12 (KCTD12)	KCTD12
Cluster-62068.88989	1.7965	0.000466	0.041583	carcinoembryonic antigen-related cell adhesion molecule 1 (biliary glycoprotein) (CEACAM1)	CEACAM
Cluster-62068.105695	1.795	9.71E-05	0.012971	lipase, endothelial (LIPG)	E3.1.1.3
Cluster-62068.89233	1.7926	1.58E-06	0.00049438	Ailuropoda melanoleuca histone H3 (LOC100471070)	H3
Cluster-62068.111169	1.7913	1.28E-09	1.21E-06	collagen, type XII, alpha 1 (COL12A1)	COL12A1
Cluster-62068.128982	1.7888	0.000212	0.023285	solute carrier family 4, sodium bicarbonate cotransporter, member 8 (SLC4A8)	SLC4A8
Cluster-62068.72006	1.7835	6.04E-05	0.0090092	alpha-2-macroglobulin-like 1 (A2ML1)	A2ML1

Cluster-62068.73607	1.776	4.21E-08	2.39E-05	Canis lupus familiaris KIAA1244 ortholog (KIAA1244)	KIAA1244
Cluster-62068.118202	1.7748	1.43E-05	0.0029065	Mustela vison xenotropic and polytropic murine leukemia virus receptor mRNA	--
Cluster-62068.96489	1.7723	2.11E-08	1.33E-05	Ailuropoda melanoleuca uncharacterized LOC105238840	--
Cluster-62068.112609	1.767	7.35E-05	0.010513	Odobenus rosmarus divergens plastin 1 (PLS1)	PLS1
Cluster-62068.105450	1.7641	0.000165	0.019526	patatin-like phospholipase domain containing 6 (PNPLA6)	NTE, NRE
Cluster-62068.96360	1.7618	0.000555	0.047071	uncharacterized LOC106005600 (LOC106005600)	--
Cluster-62068.114846	1.7592	0.000414	0.037995	Ovis canadensis canadensis isolate 43U chromosome 23 sequence	--
Cluster-62068.78502	1.7559	2.34E-05	0.0042666	DEAH (Asp-Glu-Ala-Asp/His) box polypeptide 57 (DHX57), transcript variant X7	--
Cluster-62068.128880	1.7541	9.57E-05	0.012832	RAN binding protein 3 (RANBP3)	RANBP3
Cluster-62068.95585	1.7532	3.00E-05	0.0052248	Equus caballus collagen(COL1A2)	COL1AS
Cluster-62068.83293	1.7523	0.000502	0.043814	shootin 1 (SHTN1)	SHTN1
Cluster-62068.87759	1.7477	0.000234	0.025112	dystonin (DST)6	DST
Cluster-62068.70812	1.7447	0.000536	0.046002	attractin-like 1 (ATRNL1)	ATRNL1
Cluster-62068.86535	1.7434	0.00038	0.035905	farnesyl diphosphate synthase (FDPS)	FDPS
Cluster-62068.73200	1.742	7.07E-05	0.010213	Canis lupus familiaris xenotropic and polytropic retrovirus receptor 1 (XPR1)	--
Cluster-62068.80480	1.7344	1.16E-06	0.00039316	alpha-2-macroglobulin-like 1 (A2ML1)	A2ML1
Cluster-62068.117125	1.7296	0.000141	0.017212	ADAM metallopeptidase with thrombospondin type 1 motif, 12 (ADAMTS12)	ADAMTS12
Cluster-62068.93273	1.7206	5.76E-06	0.0014229	ST3 beta-galactoside alpha-2,3-sialyltransferase 2 (ST3GAL2)	SIAT4B
Cluster-62068.71917	1.7171	0.000166	0.019587	guanine nucleotide binding protein (G protein) (GNA14)	GNA14
Cluster-62068.91464	1.7139	0.000382	0.036011	paired box 8 (PAX8)	PAX8
Cluster-62068.137413	1.7087	0.000188	0.021315	carcinoembryonic antigen-related cell adhesion molecule 1 (biliary glycoprotein) (CEACAM1)	CEACAM

Cluster-62068.96460	1.706	3.43E-05	0.0058501	Pig DNA sequence from clone CH242-206C22 on chromosome X	SNAPC4
Cluster-62068.112755	1.706	0.000339	0.032911	cholinergic receptor, nicotinic, alpha 5 (neuronal) (CHRNA5)	CHRNA5
Cluster-62068.95538	1.7032	1.54E-06	0.00048538	adenosine kinase (ADK)	ADK
Cluster-62068.104101	1.6939	2.81E-05	0.0049224	FIC domain containing (FICD)	FICD
Cluster-62068.95198	1.6915	0.00022	0.024012	solute carrier family 19 (thiamine transporter) (SLC19A2)	SLC19A2_3, THTR
Cluster-62068.93607	1.6901	0.000181	0.020785	procollagen C-endopeptidase enhancer (PCOLCE)	PCOLCE
Cluster-62068.136652	1.6887	0.00038	0.035905	cyclin-dependent kinase 18 (CDK18)	CDK18
Cluster-62068.75270	1.6844	0.000326	0.031816	pleckstrin and Sec7 domain containing 4 (PSD4)	PSD4
Cluster-62068.63050	1.6844	1.12E-05	0.0023968	periostin, osteoblast specific factor (POSTN)	POSTN
Cluster-62068.107357	1.6769	2.45E-06	0.00070447	methyltransferase like 20 (METTL20)	METTL20
Cluster-62068.91424	1.6754	0.000534	0.045874	endogenous retrovirus group PABLB member 1 Env polyprotein-like (LOC106003903)	--
Cluster-62068.105990	1.6741	0.000424	0.038553	ATPase, H+ transporting (ATP6V1A)	ATPeV1A, ATP6A
Cluster-62068.42264	1.6694	0.000363	0.034684	Pig DNA sequence from clone CH242-171I16 on chromosome 6	--
Cluster-62068.70538	1.6684	0.00049	0.043085	myoglobin (MB)	MB
Cluster-62068.97298	1.6656	2.86E-06	0.00080721	Canis familiaris chromosome X, clone XX-35E2	--
Cluster-62068.91270	1.6596	1.08E-05	0.0023245	high mobility group nucleosome binding domain 1 (HMGN1)	HMGN1
Cluster-62068.65418	1.659	3.19E-07	0.00013196	uncharacterized LOC101678065 (LOC101678065), ncRNA	--
Cluster-62068.117258	1.6564	0.000343	0.033185	doublecortin-like kinase 1 (DCLK1)	DCLK1
Cluster-62068.82088	1.6558	4.40E-05	0.00709	myocardin (MYOCD)	MYOCD
Cluster-62068.93284	1.652	0.000419	0.038292	embigin (EMB)	EMB
Cluster-62068.77728	1.6518	0.000571	0.048151	dihydrouridine synthase 4-like (S. cerevisiae) (DUS4L)	DUS4
Cluster-62068.102312	1.6482	2.44E-06	0.0007019	leucine rich repeat containing 3 (LRRC3)	LRRC3

Cluster-62068.95127	1.6418	2.09E-08	1.33E-05	Canis familiaris chromosome 16	--
Cluster-62068.107430	1.6402	0.000401	0.037334	homeobox B2 (HOXB2)	HOX_2
Cluster-62068.89711	1.6351	9.44E-06	0.0021097	solute carrier family 18 (vesicular monoamine transporter), member 2 (SLC18A2)	SLC18A1_2, VMAT
Cluster-62068.67193	1.6348	0.000498	0.043632	WDYHV motif containing 1 (WDYHV1)	WDYHV1
Cluster-62068.67128	1.6323	1.84E-05	0.0035567	trafficking protein particle complex 2 (TRAPPC2)	TRAPPC2
Cluster-62068.111798	1.6321	6.63E-05	0.0097077	ets homologous factor (EHF)	EHF
Cluster-62068.97665	1.6298	6.69E-07	0.00025028	solute carrier family 9, subfamily A (NHE8, cation proton antiporter 8), member 8 (SLC9A8)	SLC9A8, NHE8
Cluster-62068.71992	1.6269	0.000412	0.037912	solute carrier family 18 (vesicular monoamine transporter), member 2 (SLC18A2)	SLC18A1_2, VMAT
Cluster-62068.117379	1.6249	0.000154	0.018394	Cbl proto-oncogene-like 1, E3 ubiquitin protein ligase (CBLL1)	CBLL1
Cluster-62068.120490	1.6219	0.000377	0.035665	Ailuropoda melanoleuca transcription factor 12 (TCF12)	TCF12
Cluster-62068.57524	1.6205	0.000222	0.024174	transforming acidic coiled-coil-containing protein 2 (LOC101683250)	TACC2
Cluster-62068.103661	1.6168	0.000459	0.041087	Ailuropoda melanoleuca tectonic family member 1 (TCTN1)	TCTN1_3
Cluster-62068.101786	1.6162	0.000395	0.036778	spalt-like transcription factor 1 (SALL1)	SALL1
Cluster-62068.126285	1.6128	0.000301	0.030037	growth differentiation factor 7 (GDF7)	MYO5
Cluster-62068.140962	1.5984	2.70E-05	0.0047677	Odobenus rosmarus divergens peroxisomal trans-2-enoyl-CoA reductase (PECR)	PECR
Cluster-62068.82483	1.5976	2.52E-07	0.00010865	Canis lupus familiaris v-myc avian myelocytomatosis viral oncogene lung carcinoma derived homolog (MYCL)	MYCL
Cluster-62068.131326	1.5969	7.89E-05	0.011091	Canis familiaris chromosome X	--
Cluster-62068.84267	1.5892	5.42E-07	0.00020905	Ailuropoda melanoleuca autophagy related 4C, cysteine peptidase (ATG4C)	ATG4
Cluster-62068.86748	1.5825	0.000356	0.03421	KIAA0513 ortholog (KIAA0513)	KIAA0513
Cluster-62068.134241	1.58	7.03E-05	0.010178	frizzled class receptor 1 (FZD1)	FZD1_7, fz

Cluster-62068.96643	1.5761	0.000509	0.044186	SECIS binding protein 2 (SECISBP2)	SECISBP2, SBP2
Cluster-62068.119097	1.5733	2.07E-10	2.45E-07	ring finger protein 170 (RNF170)	RNF170
Cluster-62068.95962	1.5701	0.000292	0.029439	ribosomal modification protein rimK-like family member B (RIMKLB), transcript variant X7	RIMKLB, NAAGS-I
Cluster-62068.140341	1.5695	0.000431	0.0391	Leptonychotes weddellii dynamin 3 (DNM3)	DNM
Cluster-62068.57054	1.569	0.000194	0.021952	Bos taurus TBC1 domain family(TBC1D24)	TBC1D24
Cluster-62068.83671	1.5639	2.30E-05	0.0042183	potassium channel tetramerization domain containing 12 (KCTD12)	KCTD12
Cluster-62068.48224	1.5637	7.21E-05	0.010376	solute carrier family 35(SLC35F2)	SLC35F2
Cluster-62068.94853	1.5587	0.000229	0.024729	clone CH237-505N16	--
Cluster-62068.90586	1.5578	0.000275	0.028235	neuronal cell adhesion molecule (NRCAM)6	NRCAM
Cluster-62068.147772	1.5549	0.000432	0.039157	dystrobrevin, alpha (DTNA)9	DTNA
Cluster-62068.90450	1.5532	0.000268	0.027733	Ursus maritimus phosphatidylinositol 3,4,5-trisphosphate 3-phosphatase TPTE2-like (LOC103677640)	--
Cluster-62068.87546	1.5524	7.62E-05	0.010786	glutamine--fructose-6-phosphate transaminase 1 (GFPT1)	GFPT1
Cluster-62068.67900	1.5493	0.000148	0.017856	Ursus maritimus major facilitator superfamily domain containing 2A (MFSD2A)	MFSD2A
Cluster-62068.74662	1.5493	0.000574	0.048352	leucine-rich pentatricopeptide repeat containing (LRPPRC)	LRPPRC
Cluster-62068.105145	1.5474	0.000541	0.046351	sushi-repeat containing protein (SRPX)	SRPX
Cluster-62068.88791	1.5457	2.16E-06	0.00064086	neuronal cell adhesion molecule (NRCAM)5	NRCAM
Cluster-62068.117878	1.5456	3.49E-05	0.0058958	RNA binding motif (RNP1, RRM) protein 3 (RBM3)	RBM3
Cluster-62068.129670	1.5395	0.000392	0.036655	lipid droplet associated hydrolase (LDAH)	LDAH
Cluster-62068.96484	1.5366	0.00045	0.0405	adenosine kinase (ADK)	ADK
Cluster-62068.87534	1.531	1.01E-05	0.0022084	protein NipSnap homolog 3A (LOC101678130)	--
Cluster-62068.94488	1.5205	0.000276	0.028293	Panthera tigris altaica procollagen C-endopeptidase enhancer (PCOLCE)	--
Cluster-62068.119316	1.5173	0.00013	0.016314	dipeptidase 1 (renal) (DPEP1)	DPEP

Cluster-62068.80042	1.5161	1.48E-06	0.00046806	nidogen 2 (osteonidogen) (NID2)	NID
Cluster-62068.96962	1.5086	0.000145	0.017643	collagen, type I, alpha 2 (COL1A2)	COL1AS
Cluster-62068.82777	1.5083	0.00024	0.025595	Odobenus rosmarus divergens SUN domain containing ossification factor (SUCO)	--
Cluster-62068.96867	1.5065	4.93E-05	0.0076743	uncharacterized LOC101674667 (LOC101674667)	--
Cluster-62068.85565	1.5031	2.08E-05	0.0039129	BAC clone RP11-779O18 from 5	DUSP, MKP
Cluster-62068.110854	1.4985	1.56E-05	0.003111	Felis catus solute carrier family 25 (mitochondrial carrier; ornithine transporter) member 15 (SLC25A15)	SLC25A15
Cluster-62068.82776	1.4982	9.35E-05	0.012623	Odobenus rosmarus divergens SUN domain containing ossification factor (SUCO)	SUCO
Cluster-62068.94385	1.4973	0.000565	0.047792	adenylosuccinate lyase (ADSL)	purB, ADSL
Cluster-62068.117653	1.4913	0.000118	0.015122	dihydrolipoamide dehydrogenase (DLD)	DLD, lpd, pdhD
Cluster-62068.124062	1.491	0.000392	0.036655	Ursus maritimus T-cell surface glycoprotein CD1b-like (LOC103672077)	CD1
Cluster-62068.96850	1.4895	5.06E-06	0.001277	ribosomal modification protein rimK-like family member B (RIMKLB)	RIMKLB, NAAGS-I
Cluster-62068.96964	1.4847	6.44E-05	0.0094846	collagen (COL1A2)	COL1AS
Cluster-62068.121967	1.4839	1.27E-07	6.20E-05	teneurin transmembrane protein 4 (TENM4)	TENM4
Cluster-62068.97743	1.4814	0.000391	0.036618	solute carrier family 31 (copper transporter)	SLC31A1
Cluster-62068.97147	1.4801	0.000575	0.048414	CD248 molecule, endosialin (CD248)	CD248, CD164L1
Cluster-62068.111282	1.4793	0.000545	0.046562	solute carrier family 7(SLC7A4)	SLC7A4
Cluster-62068.88381	1.4764	6.05E-06	0.0014658	solute carrier family 41 (magnesium transporter)	SLC41A
Cluster-62068.90115	1.467	1.43E-06	0.00045765	tubulin tyrosine ligase (TTL)	TTL
Cluster-62068.69294	1.4654	0.000285	0.0289	coagulation factor V (proaccelerin, labile factor) (F5)	F5
Cluster-62068.92571	1.4612	2.78E-05	0.0048732	sex hormone-binding globulin (SHBG)	SHBG
Cluster-62068.107646	1.4585	0.000549	0.046732	KIAA0513 ortholog (KIAA0513)	KIAA0513

Cluster-62068.119498	1.4476	3.94E-05	0.0064664	oxysterol binding protein-like 7	OSBPL7
Cluster-62068.83822	1.4427	3.59E-05	0.0060262	calcium/calmodulin-dependent protein kinase ID (CAMK1D)	CAMK1
Cluster-62068.80463	1.4425	0.000179	0.020605	glutathione peroxidase 8 (putative) (GPX8)	GPX8
Cluster-62068.106996	1.4401	0.000226	0.024515	activated leukocyte cell adhesion molecule (ALCAM)	ALCAM
Cluster-62068.99186	1.4395	4.89E-05	0.0076412	biglycan (BGN)	BGN
Cluster-62068.105674	1.439	0.000506	0.044007	neural cell adhesion molecule 1 (NCAM1)	NCAM
Cluster-62068.93147	1.4351	4.04E-06	0.0010656	ribosomal modification protein rimK-like family member B (RIMKLB)	RIMKLB, NAAGS-I
Cluster-62068.102645	1.4342	0.000353	0.034047	signal recognition particle 19kDa (SRP19)	SRP19
Cluster-62068.105166	1.434	3.28E-06	0.00090619	enoyl-CoA, hydratase/3-hydroxyacyl CoA dehydrogenase (EHHADH)	EHHADH
Cluster-62068.78550	1.4275	0.000102	0.013459	Leptonychotes weddellii activated leukocyte cell adhesion molecule (ALCAM)	--
Cluster-62068.112169	1.4193	0.000319	0.031348	JAZF zinc finger 1 (JAZF1)	JAZF1
Cluster-62068.91247	1.4082	0.000203	0.022702	myosin VC (MYO5C)	MYO5
Cluster-62068.93146	1.4072	4.20E-08	2.39E-05	ribosomal modification protein rimK-like family member B (RIMKLB)	RIMKLB
Cluster-62068.74670	1.4031	4.28E-05	0.0069244	chromosome unknown open reading frame, human C11orf54 (LOC101688598)	--
Cluster-62068.66826	1.4023	4.42E-06	0.0011454	alpha-2-macroglobulin-like 1 (A2ML1)	A2ML1
Cluster-62068.131695	1.4017	1.07E-05	0.002313	zinc finger protein 2 homolog (LOC101685904)	KRAB
Cluster-62068.90904	1.3976	7.28E-06	0.0017174	melanoma cell adhesion molecule (MCAM)	MCAM, CD146
Cluster-62068.90945	1.3795	4.81E-06	0.0012284	ribosomal modification protein rimK-like family member B (RIMKLB)	--
Cluster-62068.103516	1.3775	8.64E-11	1.16E-07	ribosomal modification protein rimK-like family member B (RIMKLB)	RIMKLB
Cluster-62068.97087	1.3746	0.000393	0.036668	cordon-bleu WH2 repeat protein-like 1 (COBLL1)	COBLL1
Cluster-62068.94112	1.3686	9.57E-05	0.012832	neural cell adhesion molecule 1 (NCAM1)	NCAM1
Cluster-62068.67929	1.3643	0.000474	0.041961	biglycan (BGN)	BGN

Cluster-62068.100238	1.3614	0.00029	0.029206	Ras suppressor protein 1 (RSU1)	RSU1
Cluster-62068.112347	1.3605	0.000414	0.037995	superkiller viralicidic activity 2-like (SKIV2L)	SKI2, SKIV2L
Cluster-62068.98125	1.3585	8.51E-08	4.42E-05	testin LIM domain protein (TES)	TES
Cluster-62068.87314	1.357	7.13E-11	9.69E-08	ribosomal modification protein rimK-like family member B (RIMKLB)	RIMKLB
Cluster-62068.60128	1.3567	7.67E-06	0.0017909	nidogen 2 (osteonidogen) (NID2)	NID
Cluster-62068.168191	1.3483	0.000181	0.020713	shugoshin-like 1 (SGOL1)	SGOL1
Cluster-62068.88474	1.3472	9.20E-05	0.012504	NACC family member 2, BEN and BTB (POZ) domain containing (NACC2)	BTBD14
Cluster-62068.95262	1.3469	4.47E-05	0.0071682	collagen, type III, alpha 1 (COL3A1)	COL1AS
Cluster-62068.59018	1.3443	5.79E-05	0.0087105	piggyBac transposable element derived 1 (PGBD1)	PGBD1
Cluster-62068.91748	1.3403	0.000393	0.036655	sorting nexin 18 (SNX18)	SNX9_18_33
Cluster-62068.75115	1.3383	0.000256	0.026821	chromosome unknown open reading frame, human C8orf58 (LOC101672066)	--
Cluster-62068.119757	1.3368	0.00043	0.039002	prolactin receptor (PRLR)	PRLR
Cluster-62068.130202	1.3364	0.000199	0.022316	prolactin receptor (PRLR)	PRLR
Cluster-62068.76244	1.3344	0.000235	0.025194	autophagy related 4C, cysteine peptidase (ATG4C)	ATG4
Cluster-62068.67389	1.3238	0.000398	0.037027	tetraspanin 13 (TSPAN13)	TSPAN13_31
Cluster-62068.103633	1.3224	3.43E-08	2.00E-05	palmitoyl-protein thioesterase 1 (PPT1)	PPT
Cluster-62068.97146	1.3192	0.000313	0.03093	CD248 molecule, endosialin (CD248)	CD248, CD164L1
Cluster-62068.94039	1.3178	0.000462	0.041282	actin, beta (ACTB)	ACTB_G1
Cluster-62068.100232	1.317	2.10E-08	1.33E-05	Ras suppressor protein 1 (RSU1)	RSU1
Cluster-62068.78548	1.3162	0.000393	0.036655	activated leukocyte cell adhesion molecule (ALCAM)	ALCAM
Cluster-62068.42263	1.3095	0.00012	0.015335	Ovis canadensis canadensis isolate 43U chromosome 1 sequence	AUH
Cluster-62068.95741	1.3077	5.21E-05	0.008009	coenzyme Q10B (COQ10B)	COQ10
Cluster-62068.157555	1.3024	0.000276	0.028264	Pig DNA sequence from clone CH242-171I16 on chromosome 6	--
Cluster-62068.94335	1.3013	3.88E-05	0.0063872	collagen, type III, alpha 1 (COL3A1)	COL1AS
Cluster-62068.26866	1.2999	0.000193	0.021867	phosphodiesterase 7B (PDE7B)	PDE7

Cluster-62068.125742	1.2999	1.28E-06	0.00042095	basic, immunoglobulin-like variable motif containing (BIVM)	BIVM
Cluster-62068.130910	1.2945	1.23E-05	0.0025692	mitogen-activated protein kinase kinase kinase 13 (MAP3K13)	MAP3K13
Cluster-62068.92353	1.2896	0.000112	0.01449	collagen, type III, alpha 1 (COL3A1)	COL3A1
Cluster-62068.90647	1.2886	0.000173	0.020145	glutaminase (GLS)	glsA, GLS
Cluster-62068.99528	1.2865	3.07E-06	0.0008614	neuronal regeneration related protein (NREP)	NREP
Cluster-62068.122242	1.2749	0.00041	0.037876	multiple EGF-like-domains 9 (MEGF9)	MEGF9
Cluster-62068.99455	1.2736	4.97E-05	0.0077183	syncytin-1-like (LOC101693826)	--
Cluster-62068.79490	1.2729	2.21E-06	0.00065046	twisted gastrulation BMP signaling modulator 1 (TWSG1)	TWSG1
Cluster-62068.134592	1.2711	5.86E-05	0.0088023	SET binding factor 2 (SBF2)	SBF1_2, MTMR5_13
Cluster-62068.115078	1.2668	4.69E-05	0.0073888	Condylura cristata spalt-like transcription factor 1 (SALL1)	SALL1
Cluster-62068.112583	1.2667	0.00053	0.04561	protein kinase C and casein kinase substrate in neurons 2 (PACSIN2)	PACSIN2
Cluster-62068.98411	1.2593	0.000462	0.041282	tetraspanin 1 (TSPAN1)	TSPAN1
Cluster-62068.62872	1.2577	0.000132	0.016487	X-prolyl aminopeptidase (aminopeptidase P)	XPNPEP2
Cluster-62068.127778	1.2547	5.94E-06	0.0014567	sodium channel, voltage gated, type III alpha subunit (SCN3A)	SCN3A
Cluster-62068.127997	1.2518	3.47E-06	0.00094998	cyclin and CBS domain divalent metal cation transport mediator 4 (CNNM4)	CNNM
Cluster-62068.55687	1.2479	7.57E-05	0.010753	Pan troglodytes frizzled class receptor 3 (FZD3)	FZD3
Cluster-62068.108508	1.2465	2.52E-05	0.0045276	chromosome unknown open reading frame, human C14orf80 (LOC101681451)	C14orf80
Cluster-62068.66502	1.2443	4.98E-05	0.007733	lymphocyte cytosolic protein 1 (L-plastin) (LCP1)	LCP1, PLS2
Cluster-62068.94800	1.2369	0.000546	0.046578	transmembrane 9 superfamily member 3 (TM9SF3)	TM9SF3
Cluster-62068.67673	1.2366	0.000111	0.014379	phosphoserine phosphatase (PSPH)	serB, PSPH
Cluster-62068.96716	1.2348	0.000179	0.020627	lumican (LUM)	LUM
Cluster-62068.96206	1.23	0.000282	0.028715	Mus musculus 9.5 days embryo parthenogenote cDNA, RIKEN full-length enriched library, clone:B130020O11 product:procollagen, type III, alpha 1,	COL1AS

				full insert sequence	
Cluster-62068.104418	1.2286	6.56E-05	0.0096318	squamous cell carcinoma antigen recognized by T cells 3 (SART3)	--
Cluster-62068.70770	1.2285	0.000193	0.021844	basic helix-loop-helix family, member a15 (BHLHA15)	BHLHB8, MIST1
Cluster-62068.147937	1.2279	0.000417	0.038181	dysferlin (DYSF)6	DYSF
Cluster-62068.94234	1.2263	4.24E-06	0.0011109	gem (nuclear organelle) associated protein 6 (GEMIN6)	GEMIN6
Cluster-62068.93617	1.2258	4.59E-05	0.0072919	solute carrier family 5 (sodium/multivitamin and iodide cotransporter), member 6 (SLC5A6)	SLC5A6, SMVT
Cluster-62068.116420	1.2198	9.14E-05	0.012464	exportin 5 (XPO5)	XPO5
Cluster-62068.92681	1.2198	0.000319	0.031348	transforming growth factor, beta 2 (TGFB2)	TGFB2
Cluster-62068.112622	1.2163	0.000204	0.022705	patatin-like phospholipase domain containing 8 (PNPLA8)	PNPLA8
Cluster-62068.71969	1.2129	0.000108	0.014166	DnaJ (Hsp40) homolog, subfamily B, member 9 (DNAJB9)	DNAJB9
Cluster-62068.135515	1.2115	4.61E-06	0.0011895	bone morphogenetic protein receptor, type IB (BMPR1B)	BMPR1B, ALK6
Cluster-62068.83916	1.2048	0.000294	0.029554	transmembrane protein 245 (TMEM245)	TMEM245
Cluster-62068.98172	1.1917	0.000469	0.041688	CDC-like kinase 1 (CLK1)	CLK
Cluster-62068.96889	1.1893	6.98E-06	0.0016626	protein tyrosine phosphatase, receptor type, F (PTPRF), transcript variant X4	PTPRF, LAR
Cluster-62068.90188	1.1882	1.61E-06	0.00049893	Leptonychotes weddellii integrin beta 1 binding protein (melusin) 2 (ITGB1BP2)	NONO, NRB54
Cluster-62068.89639	1.1847	2.90E-05	0.0050673	myosin light chain kinase (MYLK)	MYLK
Cluster-62068.110238	1.1785	0.000289	0.029188	coproporphyrinogen oxidase (CPOX)	CPOX, hemF
Cluster-62068.114833	1.1696	0.000503	0.043831	Canis lupus familiaris DEAD (Asp-Glu-Ala-Asp) box polypeptide 46 (DDX46)	DDX46, PRP5
Cluster-62068.133689	1.1692	5.80E-06	0.001429	Canis lupus familiaris nudix (nucleoside diphosphate linked moiety X)-type motif 12 (NUDT12)	NUDT12, nudC
Cluster-62068.65695	1.1661	2.90E-05	0.0050687	chromosome unknown open reading frame, human C9orf72 (LOC101687732)	C9orf72
Cluster-62068.106457	1.1645	0.000494	0.043352	collagen, type V, alpha 2 (COL5A2)	COL1AS

Cluster-62068.104102	1.1587	0.000173	0.020145	FIC domain containing (FICD)	FICD
Cluster-62068.133924	1.1546	7.11E-06	0.0016858	GrpE-like 2, mitochondrial (E. coli) (GRPEL2)	GRPE
Cluster-62068.111438	1.1429	0.000145	0.017573	solute carrier family 39, member 11 (SLC39A11)	SLC39A11, ZIP11
Cluster-62068.129357	1.1418	0.00013	0.01629	sema domain, immunoglobulin domain (Ig), transmembrane domain (TM) and short cytoplasmic domain, (semaphorin) 4G (SEMA4G)	SEMA4
Cluster-62068.87459	1.1394	0.000252	0.026575	heat-responsive protein 12 (HRSP12)	HRSP12
Cluster-62068.106523	1.1367	4.91E-05	0.0076596	sorting nexin 2 (SNX2)	SNX1_2
Cluster-62068.70996	1.1327	4.32E-06	0.0011253	URB2 ribosome biogenesis 2 homolog (S. cerevisiae) (URB2)	URB2
Cluster-62068.123631	1.1324	3.57E-05	0.0060081	zinc finger, DHHC-type containing 13 (ZDHHC13)	ZDHHC
Cluster-62068.119758	1.1278	6.22E-05	0.0092356	prolactin receptor (PRLR)	PRLR
Cluster-62068.78251	1.1269	0.000187	0.021314	nuclear transport factor 2-like export factor 2 (NXT2)	NXT2
Cluster-62068.63278	1.1265	0.000384	0.036108	haloacid dehalogenase-like hydrolase domain containing 2 (HDHD2)	HDHD2
Cluster-62068.96791	1.1244	8.82E-05	0.0121	tropomyosin 1 (alpha) (TPM1)5	TPM1
Cluster-62068.70838	1.1239	3.25E-06	0.00090384	lipase, endothelial (LIPG)	LIPG
Cluster-62068.96255	1.1055	5.61E-06	0.001389	Odobenus rosmarus divergens sphingosine-1-phosphate phosphatase 1 (SGPP1)	SGPP1
Cluster-62068.94920	1.105	0.00054	0.046254	Ursus maritimus galactokinase 2 (GALK2)	GALK2
Cluster-62068.83861	1.0996	0.000115	0.01483	solute carrier family 5 (sodium/myo-inositol cotransporter), member 3 (SLC5A3)	SLC5A3
Cluster-62068.108292	1.0931	6.59E-05	0.0096611	Felis catus glypican 6 (GPC6)	GPC6
Cluster-62068.39780	1.0929	0.000119	0.015252	Leptonychotes weddellii IKBKB interacting protein (IKBIP)	IKBIP
Cluster-62068.88170	1.0903	0.000113	0.014616	rhophilin, Rho GTPase binding protein 2 (RHPN2)	RHPN2
Cluster-62068.86408	1.0885	8.62E-06	0.0019731	Pig DNA sequence from clone CH242-78D20 on chromosome X	--
Cluster-62068.137425	1.0877	0.000253	0.026609	RIO kinase 3 (RIOK3)	RIOK3, SUDD
Cluster-62068.62098	1.0838	3.48E-05	0.0058916	kelch-like family member 18 (KLHL18)	KLHL18

Cluster-62068.72702	1.0832	9.76E-06	0.0021562	FUN14 domain containing 1 (FUNDC1)	FUNDC1
Cluster-62068.82383	1.0789	7.99E-05	0.011169	chromosome unknown open reading frame, human C9orf41 (LOC101692030)	--
Cluster-62068.74030	1.0673	1.50E-05	0.0030116	potassium channel, two pore domain subfamily K, member 6 (KCNK6)	KCNK6
Cluster-62068.121704	1.0529	0.000352	0.03396	solute carrier family 7 (SLC7A1)	SLC7A1
Cluster-62068.96397	1.0517	0.000478	0.042185	tumor suppressor candidate 3 (TUSC3)	OST3, OST6
Cluster-62068.58741	1.0352	0.000246	0.026112	cyclin G2 (CCNG2)	CCNG2
Cluster-62068.92721	1.0289	9.97E-05	0.013248	GTPase activating protein (SH3 domain) binding protein 2 (G3BP2)	G3BP2
Cluster-62068.125021	1.0256	0.000578	0.048665	Odobenus rosmarus divergens rab GTPase-activating protein 1-like, isoform 10 (LOC101363017)	--
Cluster-62068.71703	1.025	4.10E-09	3.39E-06	aconitase 1, soluble (ACO1)	ACO, acnA
Cluster-62068.91157	1.0207	2.94E-05	0.0051305	Ailuropoda melanoleuca spalt-like transcription factor 4 (SALL4)	SALL4
Cluster-62068.92209	1.0202	0.000224	0.024382	tyrosine 3-monooxygenase/tryptophan 5-monooxygenase activation protein, theta (YWHAQ)	YWHAB_Q_Z
Cluster-62068.83814	1.0141	0.000207	0.022951	Sus scrofa transitional endoplasmic reticulum ATPase-like (LOC100516776)	VCP, CDC48
Cluster-62068.94099	1.0015	0.000546	0.046588	trafficking protein particle complex 11 (TRAPPC11)	TRAPPC11
Cluster-62068.86649	1.0007	0.00021	0.023223	Canis familiaris, clone XX-237B24	--
Cluster-62068.77803	0.99863	0.000165	0.01955	forkhead box A2 (FOXA2)	FOXA2, HNF3B
Cluster-62068.132943	0.99564	0.000309	0.030624	uncharacterized LOC106005084 (LOC106005084)	--
Cluster-62068.82625	0.99501	0.000527	0.045436	calcineurin-like EF-hand protein 1 (CHP1)	CHP, CHP1
Cluster-62068.103568	0.99025	0.000314	0.030961	3-hydroxy-3-methylglutaryl-CoA reductase (HMGCR)	HMGCR
Cluster-62068.101785	0.98758	4.80E-05	0.007538	Ailuropoda melanoleuca spalt-like transcription factor 1 (SALL1)	SALL1
Cluster-62068.107172	0.97732	9.42E-05	0.01268	acyl-CoA synthetase long-chain family member 4 (ACSL4)	ACSL, fadD
Cluster-62068.84549	0.96456	0.000585	0.049108	translocated promoter region, nuclear basket protein (TPR)	TPR, MLP1, MLP2
Cluster-62068.103711	0.96236	0.00013	0.016289	Ursus maritimus low density lipoprotein receptor-related protein 2 (LRP2)	LRP2

Cluster-62068.131545	0.95801	0.000314	0.030961	helicase, lymphoid-specific (HELLS)	HELLS, DDM1
Cluster-62068.74607	0.94626	0.000366	0.034905	protein NipSnap homolog 3A (LOC101679135)	--
Cluster-62068.99661	0.94349	0.000521	0.045085	pyruvate dehydrogenase kinase, isozyme 1 (PDK1)	PDK1
Cluster-62068.76167	0.93668	0.0001	0.013322	tetratricopeptide repeat domain 39A (TTC39A)	TTC39A
Cluster-62068.109041	0.93027	8.54E-05	0.011808	G protein-coupled receptor kinase interacting ArfGAP 1 (GIT1)	GIT1
Cluster-62068.83847	0.92311	0.000188	0.021388	epithelial splicing regulatory protein 2 (ESRP2)	ESRP1_2
Cluster-62068.65154	0.91807	0.000118	0.015151	Odobenus rosmarus divergens JAZF zinc finger 1 (JAZF1)	JAZF1
Cluster-62068.55983	0.91522	0.00037	0.035257	polymerase (RNA) III (DNA directed) polypeptide E (80kD) (POLR3E)	RPC5, POLR3E
Cluster-62068.104356	0.9131	0.00038	0.035916	pleiotrophin (PTN)	PTN
Cluster-62068.111992	0.90988	0.000508	0.044108	protein tyrosine phosphatase, receptor type, D (PTPRD)3	PTPRD
Cluster-62068.106706	0.88662	1.10E-05	0.0023572	Felis catus RAB GTPase activating protein 1-like (RABGAP1L)	RABGAP1L
Cluster-62068.121796	0.86341	0.000553	0.047	phosphatidylinositol 3-kinase, catalytic subunit type 3 (PIK3C3)	PIK3C3, VPS34
Cluster-62068.89954	0.84741	1.82E-05	0.003528	myosin ID (MYO1D)	MYO1
Cluster-62068.96914	0.82964	0.000503	0.043814	nudE neurodevelopment protein 1 (NDE1)	NDE1, NUDE
Cluster-62068.121618	0.81405	0.000224	0.024382	Leptonychotes weddellii WW domain containing E3 ubiquitin protein ligase 1 (WWP1)	AIP5, WWP1
Cluster-62068.94691	0.80915	0.000407	0.037744	cytidine monophosphate (UMP-CMP) kinase 1, cytosolic (CMPK1)	CMPK1, UMPK
Cluster-62068.70730	0.80084	0.000191	0.021651	staufen double-stranded RNA binding protein 1 (STAU1)	STAU
Cluster-62068.131267	0.79407	0.000201	0.022534	Felis catus cyclin C (CCNC)	CCNC
Cluster-62068.135883	0.79173	0.000109	0.014203	microtubule-associated protein 9 (MAP9)	MAP9
Cluster-62068.109915	0.76564	7.53E-05	0.010717	iron-responsive element binding protein 2 (IREB2)	IREB2
Cluster-62068.114752	0.76092	0.000581	0.048781	acyl-CoA dehydrogenase, C-4 to C-12 straight chain (ACADM)	ACADM, acd
Cluster-62068.68527	0.75139	0.000575	0.048414	Ursus maritimus insulin-degrading enzyme (IDE)	IDE, ide
Cluster-62068.74211	0.73249	8.68E-06	0.0019805	Leptonychotes weddellii cell division cycle 14A (CDC14A)	CDC14
Cluster-62068.72018	0.72973	0.000588	0.049209	zinc finger and SCAN domain containing 29 (ZSCAN29)	KRAB

Cluster-62068.99656	0.6981	0.000412	0.037912	GRAM domain containing 1B (GRAMD1B)	GRAMD1B
Cluster-62068.105471	-0.5629	0.000569	0.048108	signal-regulatory protein alpha (SIRPA)	SIRPA
Cluster-62068.96260	-0.7567	5.13E-05	0.0079259	odd-skipped related transciption factor 2 (OSR2)	OSR, ODD
Cluster-62068.111828	-0.8103	5.36E-05	0.0082006	odd-skipped related transciption factor 2 (OSR2)	OSR, ODD
Cluster-62068.121058	-0.8196	4.65E-05	0.0073499	Leptonychotes weddellii wingless-type MMTV integration site family, member 5A (WNT5A)	WNT11
Cluster-62068.77697	-0.8322	0.000414	0.037995	transforming growth factor, beta receptor III (TGFBR3)	TGFBR3
Cluster-62068.96413	-0.8348	0.000461	0.041206	retrotransposon gag domain containing 4 (RGAG4)	RGAG4
Cluster-62068.120738	-0.916	0.000294	0.029554	thrombospondin 2 (THBS2)	THBS2S
Cluster-62068.82287	-0.925	0.00057	0.048151	RNA binding motif protein 23 (RBM23)	RBM23
Cluster-62068.45619	-0.9652	8.21E-05	0.011405	Ursus maritimus plexin domain containing 2 (PLXDC2), misc_RNA	PLXDC2
Cluster-62068.86089	-0.9679	0.000411	0.037912	CD74 molecule, major histocompatibility complex, class II invariant chain (CD74)	CD74, DHLAG
Cluster-62068.109962	-0.99	0.000411	0.037912	desmoglein 2 (DSG2)	DSG2
Cluster-62068.112601	-1.0027	2.43E-06	0.00070011	F-box protein 32 (FBXO32)	FBXO25_32
Cluster-62068.119569	-1.0171	2.48E-05	0.004474	uncharacterized LOC106004563 (LOC106004563)	--
Cluster-62068.29075	-1.0298	1.46E-05	0.0029598	DNA-damage-inducible transcript 4-like (DDIT4L)	DDIT4L
Cluster-62068.59484	-1.0687	0.00013	0.016336	Odobenus rosmarus divergens tumor necrosis factor (ligand) superfamily, member 12 (TNFSF12)	TNFSF12, TWEAK
Cluster-62068.109190	-1.0701	0.000363	0.034747	solute carrier family 7 (amino acid transporter light chain, bo,+ system), member 9 (SLC7A9)	SLC7A9, BAT1
Cluster-62068.67730	-1.0703	4.84E-05	0.0075952	natriuretic peptide receptor 1 (NPR1)	ANPRA, NPR1
Cluster-62068.35410	-1.0744	0.000132	0.016512	Nance-Horan syndrome(NHS)	NHS
Cluster-62068.56604	-1.0768	0.000308	0.030541	Leptonychotes weddellii zinc finger protein 717 (ZNF717)	KRAB
Cluster-62068.116265	-1.0882	0.000321	0.031453	F-box protein 32 (FBXO32)	FBXO25_32

Cluster-62068.131785	-1.1202	0.00029	0.029206	aminomethyltransferase (AMT)	gcvT, AMT
Cluster-62068.108960	-1.1372	0.000278	0.028463	KN motif and ankyrin repeat domains 2 (KANK2)	KANK2
Cluster-62068.46338	-1.1442	8.86E-05	0.012143	histamine receptor H1 (HRH1)	HRH1
Cluster-62068.110053	-1.1758	0.00046	0.041102	lysine (K)-specific methyltransferase 2E (KMT2E)	KMT2E
Cluster-62068.134171	-1.1772	3.84E-06	0.0010236	leucine-rich repeat containing G protein-coupled receptor 5 (LGR5)	LGR5, GPR49
Cluster-62068.98293	-1.1949	0.000118	0.015112	filamin A, alpha (FLNA)	FLNA
Cluster-62068.88738	-1.1979	0.000141	0.017264	trypsin domain containing 1 (TYSND1)	TYSND1
Cluster-62068.163425	-1.1981	5.51E-05	0.0083748	B-cell CLL/lymphoma 2 (BCL2)	BCL2
Cluster-62068.94683	-1.2114	3.58E-06	0.00097064	integrin, beta 4 (ITGB4)	ITGB4
Cluster-62068.92707	-1.2156	0.000247	0.026215	Fanconi anemia, complementation group G (FANCG)	FANCG
Cluster-62068.63929	-1.2279	1.19E-06	0.00039949	transmembrane protein 200C (TMEM200C)	TMEM200C
Cluster-62068.59733	-1.2301	0.000389	0.036452	lipase A, lysosomal acid, cholesterol esterase (LIPA)	LIPA
Cluster-62068.103536	-1.239	0.000464	0.041418	ATP-binding cassette, sub-family C (CFTR/MRP), member 5 (ABCC5)	ABCC5
Cluster-62068.86742	-1.2607	0.000233	0.025106	HOP homeobox (HOPX)	HOPX
Cluster-62068.130035	-1.2665	0.000211	0.023223	fucosyltransferase 10 (alpha (1,3) fucosyltransferase) (FUT10)	FUT10
Cluster-62068.56789	-1.2711	0.000438	0.03961	Canis lupus familiaris mitochondrial ribosome recycling factor (MRRF)	COL4A
Cluster-62068.156657	-1.2926	0.000124	0.015688	solute carrier family 7 (amino acid transporter light chain, bo,+ system), member 9 (SLC7A9)	SLC7A9
Cluster-62068.114313	-1.3021	7.90E-09	5.80E-06	adrenergic, alpha-2C-, receptor (ADRA2C) gene	ADRA2C
Cluster-62068.169907	-1.3046	1.59E-06	0.00049462	phospholipase A2, group IID (PLA2G2D)	PLA2G, SPLA2
Cluster-62068.92330	-1.3082	0.000409	0.037857	integrin, beta 8 (ITGB8), transcript variant X6	ITGB8
Cluster-62068.121404	-1.3195	5.46E-05	0.0083261	progesterone receptor (PGR)	NR3C3, PGR
Cluster-62068.111388	-1.3295	0.000116	0.014953	collagen, type XXVII, alpha 1 (COL27A1)	COL1AS
Cluster-62068.52307	-1.382	1.53E-05	0.0030707	transmembrane protein 200C (TMEM200C)	TMEM200C
Cluster-62068.130666	-1.3837	0.000212	0.023307	dishevelled associated activator of morphogenesis 1 (DAAM1)	DAAM

Cluster-62068.141260	-1.3959	0.000457	0.040926	Ovis canadensis canadensis isolate 43U chromosome 14 sequence	--
Cluster-62068.126402	-1.4167	0.000187	0.021314	Pongo abelii BAC clone CH276-451O22 from chromosome 14	--
Cluster-62068.61596	-1.4324	2.15E-05	0.0039976	insulin-like growth factor binding protein 3 (IGFBP3)	--
Cluster-62068.159147	-1.434	0.000276	0.028264	benzodiazepine receptor (peripheral) associated protein 1 (BZRAP1)1	--
Cluster-62068.100858	-1.4365	2.03E-06	0.00061114	complement component 4A (Rodgers blood group) (C4A)	C4
Cluster-62068.113945	-1.4397	0.000172	0.02014	chromosome unknown open reading frame, human C16orf58 (LOC101670422)	--
Cluster-62068.170910	-1.4433	7.82E-06	0.0018198	calcium/calmodulin-dependent protein kinase IG (CAMK1G)	CAMK1
Cluster-62068.56307	-1.4441	4.56E-05	0.00725	glycerol-3-phosphate dehydrogenase 1 (soluble) (GPD1)	GPD1
Cluster-62068.93829	-1.4472	0.000458	0.041032	HOP homeobox (HOPX)	HOPX
Cluster-62068.141259	-1.4479	0.000579	0.048665	Ovis canadensis canadensis isolate 43U chromosome 14 sequence	--
Cluster-62068.39755	-1.4512	0.000552	0.046917	cap methyltransferase 1 (CMTR1)1	CMTR1, FTSJD2, MTR1
Cluster-62068.139945	-1.4548	8.15E-08	4.25E-05	EGF containing fibulin-like extracellular matrix protein 1 (EFEMP1)	EFEMP1, FBLN3
Cluster-62068.113109	-1.4568	1.48E-05	0.0029874	spectrin repeat containing, nuclear envelope 1 (SYNE1)	SYNE1
Cluster-62068.30092	-1.4673	6.60E-05	0.0096704	glutamate-rich 3 (ERICH3)	ERICH3
Cluster-62068.81105	-1.4808	0.000142	0.017274	signal transducer and activator of transcription 5B (STAT5B)	STAT5A
Cluster-62068.106868	-1.4839	8.13E-05	0.011342	CD44 molecule (Indian blood group) (CD44)5	CD44
Cluster-62068.143608	-1.488	0.000309	0.030624	laminin, alpha 3 (LAMA3)	LAMA3_5
Cluster-62068.10562	-1.5003	0.000281	0.028605	WD repeat domain 90 (WDR90)	WDR90
Cluster-62068.98045	-1.5037	0.000253	0.026575	achalasia, adrenocortical insufficiency, alacrimia (AAAS)	AAAS
Cluster-62068.32574	-1.5047	5.57E-05	0.0084387	ankyrin repeat domain 6 (ANKRD6)	ANKRD6
Cluster-62068.47861	-1.5155	2.33E-06	0.00067763	OTU deubiquitinase 1 (OTUD1)	OTUD1
Cluster-62068.61815	-1.535	0.000491	0.043099	Felis catus clone RP86-117J4	--
Cluster-62068.106867	-1.5521	3.66E-10	3.90E-07	Canis lupus familiaris CD44 molecule (Indian blood group) (CD44)	CD44

Cluster-62068.134116	-1.5584	0.000446	0.040166	protein phosphatase 6, regulatory subunit 1 (PPP6R1)	PPP6R1, SAPS1
Cluster-59433.0	-1.5624	0.000511	0.044355	genomic DNA, chromosome 18 clone:RP11-793A3	--
Cluster-62068.46253	-1.5695	0.000499	0.043678	leucine-rich repeat containing G protein-coupled receptor 5 (LGR5)	LGR5, GPR49
Cluster-62068.103706	-1.5743	7.32E-06	0.0017245	MAS-related GPR, member F (MRGPRF), transcript variant X7	MRGPRF
Cluster-62068.130867	-1.5749	3.41E-07	0.00013944	Felis catus BAC clone FCAB-33H12 from chromosome unknown	--
Cluster-62068.136476	-1.5835	0.000252	0.026575	mucin 16, cell surface associated (MUC16)3	MUC16
Cluster-62068.47860	-1.5996	1.98E-07	8.96E-05	Odobenus rosmarus divergens OTU deubiquitinase 1 (OTUD1)	OTUD1
Cluster-62068.77058	-1.6007	3.73E-05	0.0062056	synaptotagmin X (SYT10)	--
Cluster-62068.108961	-1.6009	0.000276	0.028293	KN motif and ankyrin repeat domains 2 (KANK2)	KANK2
Cluster-62068.138269	-1.6039	0.000166	0.019591	SAP30 binding protein (SAP30BP)	SAP30BP
Cluster-62068.105692	-1.606	0.000539	0.046254	plexin D1 (PLXND1)	PLXND
Cluster-62068.93226	-1.6174	9.70E-06	0.0021443	MAP-kinase activating death domain (MADD)	MADD
Cluster-62068.78941	-1.6303	2.01E-05	0.0038158	tubulin tyrosine ligase-like family member 3 (TTLL3)	TTLL3_8
Cluster-62068.77876	-1.6401	3.00E-05	0.0052248	peptidase domain containing associated with muscle regeneration 1 (PAMR1)	PAMR1
Cluster-62068.123202	-1.6409	0.00056	0.047413	Ailuropoda melanoleuca uncharacterized LOC105236424 (LOC105236424)	--
Cluster-62068.124657	-1.652	2.36E-05	0.0042933	Ursus maritimus plexin domain containing 1 (PLXDC1)	PLXDC1
Cluster-62068.85496	-1.6636	0.000494	0.043352	RAS protein activator like 1 (GAP1 like) (RASAL1)	RASAL1
Cluster-62068.24520	-1.6686	9.21E-05	0.012504	solute carrier family 6 , member 20 (SLC6A20)	SLC6A20
Cluster-62068.134801	-1.6709	0.000514	0.044553	WD repeat domain 66 (WDR66), RefSeqGene on chromosome 12	WDR66
Cluster-62068.64862	-1.6724	0.000216	0.023669	BARX homeobox 2 (BARX2)	BARX
Cluster-62068.106076	-1.6846	7.46E-05	0.010631	integrin, beta 4 (ITGB4), transcript variant X4	ITGB4
Cluster-62068.108579	-1.6851	1.84E-06	0.00055889	Pongo abelii progesterone receptor (PGR)	PGR
Cluster-62068.116157	-1.6929	0.000227	0.024526	leucine-rich repeat containing G protein-coupled receptor 5 (LGR5)	LGR5, GPR49
Cluster-62068.63550	-1.7203	5.44E-06	0.0013633	Ailuropoda melanoleuca zinc finger protein 692 (ZNF692)	ZNF692
Cluster-62068.31434	-1.739	0.000174	0.020215	glutamate-rich 3 (ERICH3)	ERICH3

Cluster-62068.183579	-1.7517	1.82E-06	0.00055331	FAT atypical cadherin 3 (FAT3)	FAT3
Cluster-62068.125025	-1.7562	0.000233	0.025079	K(lysine) acetyltransferase 2A (KAT2A)	PCAF, KAT2, GCN5
Cluster-62068.126277	-1.7753	6.27E-05	0.009281	toll-like receptor 4 (TLR4)	--
Cluster-62068.104135	-1.7779	0.000294	0.029553	TraB domain containing 2B (TRABD2B)	TRABD2B
Cluster-62068.74732	-1.801	1.43E-06	0.00045765	angel homolog 1 (Drosophila) (ANGEL1)	ANGEL
Cluster-62068.13319	-1.8148	0.000274	0.028166	pleckstrin homology domain containing, family S member 1 (PLEKHS1)	PLEKHS1
Cluster-62068.71602	-1.8165	0.000284	0.028802	chromosome unknown open reading frame, human C10orf10 (LOC101689972)	C10orf10
Cluster-62068.61809	-1.8306	9.37E-06	0.0021036	Ailuropoda melanoleuca ATP-binding cassette, sub-family C (CFTR/MRP), member 3 (ABCC3)	ABCC3
Cluster-62068.57750	-1.8386	0.00023	0.02474	Ovis canadensis canadensis isolate 43U chromosome 15 sequence	--
Cluster-62068.50958	-1.8457	2.50E-05	0.0045057	ceruloplasmin (ferroxidase) (CP)	CP
Cluster-62068.149607	-1.8708	0.000592	0.049468	synaptojanin 2 (SYNJ2)	SYNJ2
Cluster-62068.61200	-1.8791	9.51E-05	0.01277	haplotype 1 alpha2C adrenergic receptor (ADRA2C) gene	ADRA2C
Cluster-62068.85145	-1.8806	6.41E-05	0.0094592	CD44 molecule (Indian blood group) (CD44)	CD44
Cluster-62068.58800	-1.9072	0.000525	0.04529	proline and serine rich 2 (PROSER2)	PROSER2
Cluster-62068.167517	-1.9092	0.000113	0.014574	odd-skipped related 1 (Drosophila) (OSR1) gene	OSR1
Cluster-62068.16781	-1.9105	9.86E-06	0.0021647	glutamate-rich 3 (ERICH3)	--
Cluster-62068.47859	-1.9265	3.64E-05	0.0060805	Human DNA sequence from clone RP11-426A21 on chromosome 10	--
Cluster-62068.51321	-1.9271	2.70E-06	0.00076604	Odobenus rosmarus divergens intraflagellar transport 172 (IFT172)	IFT172
Cluster-62068.63646	-1.9326	0.000113	0.014616	immunoglobulin superfamily, DCC subclass, member 4 (IGDCC4)	IGDCC4
Cluster-62068.146190	-1.9373	2.75E-05	0.0048427	cytochrome P450 4A11 (LOC101674954)	CYP4A11
Cluster-62068.143430	-1.9406	2.02E-06	0.00060713	ADAM metallopeptidase domain 33 (ADAM33)1	ADAM33
Cluster-62068.121033	-1.9428	2.19E-05	0.0040562	FXYD domain containing ion transport regulator 1 (FXYD1)	FXYD1, PLM

Cluster-62068.125195	-1.9449	4.01E-11	5.88E-08	neuronal guanine nucleotide exchange factor (NGEF)	NGEF, EPHEXIN
Cluster-62068.125193	-1.9503	0.000141	0.017264	plexin A2 (PLXNA2)	PLXNA
Cluster-62068.131048	-1.9649	1.31E-05	0.0027109	family with sequence similarity 160, member B1 (FAM160B1)	--
Cluster-62068.17263	-1.9683	0.000183	0.020885	Ovis canadensis canadensis isolate 43U chromosome 19 sequence	--
Cluster-62068.40461	-1.9896	3.12E-05	0.0053979	Odobenus rosmarus divergens neuronal guanine nucleotide exchange factor (NGEF)	NGEF, EPHEXIN
Cluster-62068.45543	-2.0173	5.59E-05	0.0084559	uncharacterized LOC106004170 (LOC106004170)	--
Cluster-62068.73913	-2.0375	4.51E-10	4.72E-07	CD44 molecule (Indian blood group) (CD44)	CD44
Cluster-62068.26807	-2.0528	7.01E-05	0.010153	chromosome unknown open reading frame, human C1orf168 (LOC101670515)	--
Cluster-62068.88810	-2.0538	1.42E-05	0.0028945	CLK4-associating serine/arginine rich protein (CLASRP)	SFRS16
Cluster-62068.125769	-2.0546	4.97E-06	0.0012594	ceruloplasmin (ferroxidase) (CP)	CP
Cluster-62068.155328	-2.0548	0.00018	0.020648	ADAMTS-like 4 (ADAMTSL4)	ADAMTSL4
Cluster-62068.101612	-2.0606	0.000158	0.018826	Sus scrofa CGRP receptor component (CRCP)	CRCP
Cluster-62068.58226	-2.0718	1.16E-07	5.81E-05	Rhinolophus ferrumequinum retrovirus isolate RfRV	--
Cluster-62068.65080	-2.0729	2.23E-09	1.96E-06	MMS19 cytosolic iron-sulfur assembly component (MMS19)	MET18, MMS19
Cluster-62068.125196	-2.0896	3.50E-07	0.00014226	plexin A2 (PLXNA2)	PLXNA2
Cluster-62068.53401	-2.0955	0.000251	0.026502	IKAROS family zinc finger 4 (Eos) (IKZF4)1	IKZF, ZNFN1A
Cluster-62068.77354	-2.1071	9.18E-05	0.012491	complement component 4A (Rodgers blood group) (C4A)	C4
Cluster-62068.34910	-2.1277	1.29E-05	0.0026899	peptidyl arginine deiminase(PADI2)	--
Cluster-62068.86915	-2.1456	0.000417	0.038181	G protein-coupled receptor 161 (GPR161)	GPR161
Cluster-62068.48665	-2.1598	3.52E-06	0.00095769	pleiomorphic adenoma gene-like 1 (PLAGL1)	PLAGL1
Cluster-62068.20770	-2.1695	0.00017	0.019938	Rho guanine nucleotide exchange factor (GEF) 10 (ARHGEF10)	ARHGEF10
Cluster-62068.112980	-2.1803	0.000324	0.031707	steroid 21-hydroxylase (LOC101689319)	TN
Cluster-62068.57805	-2.1829	0.000555	0.047071	Odobenus rosmarus divergens RAS protein activator like 1 (RASAL1)	RASAL1

Cluster-62068.102115	-2.1922	0.000585	0.049108	lon peptidase 1 (LONP1)	PRSS15, PIM1
Cluster-62068.157520	-2.1931	1.99E-07	8.97E-05	Ailuropoda melanoleuca prokineticin 2 (PROK2)	PROK2
Cluster-62068.61599	-2.2047	3.55E-05	0.0059767	insulin-like growth factor binding protein 3 (IGFBP3)	IGFBP3
Cluster-62068.96059	-2.2147	7.47E-05	0.010634	proline rich membrane anchor 1 (PRIMA1)	SEPT3_9_12
Cluster-62068.116118	-2.2268	1.08E-05	0.0023245	T-cell, immune regulator 1, ATPase, H+ transporting, lysosomal V0 subunit A3 (TCIRG1)	ATPeV0A, ATP6N
Cluster-62068.164291	-2.2268	0.000234	0.025112	uncharacterized LOC101679780 (LOC101679780)	--
Cluster-62068.69758	-2.2294	1.15E-07	5.80E-05	Leptonychotes weddellii insulin-like growth factor-binding protein 3-like (LOC102738536)	--
Cluster-62068.97727	-2.2382	3.82E-05	0.0063015	RIMS binding protein 2 (RIMBP2)	RIMBP2
Cluster-62068.17620	-2.2382	6.45E-05	0.0094903	chromosome unknown open reading frame, human C1orf168 (LOC101670515)	--
Cluster-62068.308	-2.2503	0.000136	0.016866	Canis lupus familiaris uncharacterized LOC102155364 (LOC102155364), misc_RNA	--
Cluster-62068.100587	-2.2541	1.57E-05	0.0031311	uncharacterized LOC101689364 (LOC101689364)	--
Cluster-62068.76123	-2.2545	1.40E-09	1.31E-06	NDRG family member 2 (NDRG2)	NDRG2
Cluster-62068.118731	-2.2753	0.000183	0.020943	actin binding LIM protein family (ABLIM2)4	ABLIM
Cluster-62068.28639	-2.2827	0.000155	0.018479	polypeptide N-acetylgalactosaminyltransferase 15 (GALNT15)	GALNT
Cluster-62068.125194	-2.2856	1.58E-05	0.0031423	neuronal guanine nucleotide exchange factor (NGEF)	NGEF, EPHEXIN
Cluster-62068.121405	-2.2912	2.10E-08	1.33E-05	progesterone receptor (PGR)	NR3C3, PGR
Cluster-62068.34908	-2.294	1.88E-05	0.0036007	peptidyl arginine deiminase(PADI2)	E3.5.3.15
Cluster-62068.67996	-2.3088	0.000308	0.030531	ZPR1 zinc finger (ZPR1)	K06874
Cluster-62068.100650	-2.3173	2.15E-06	0.00063886	hydroxyprostaglandin dehydrogenase 15-(NAD) (HPGD)	HPGD
Cluster-62068.161213	-2.3256	3.73E-05	0.0062056	WAP, follistatin/kazal, immunoglobulin, kunitz and netrin domain containing 2 (WFIKKN2)	--

Cluster-62068.86747	-2.3397	8.39E-06	0.0019297	mesoderm development candidate 2 (MESDC2)	MESDC2
Cluster-62068.49578	-2.3615	7.77E-06	0.0018108	thyroglobulin (TG)	TG
Cluster-62068.145374	-2.3828	0.000173	0.020145	glutathione S-transferase theta 2B (GSTT2B)	GST, gst
Cluster-62068.37816	-2.3937	0.000367	0.034966	regulatory factor X, 2 (influences HLA class II expression) (RFX2), transcript variant X6	RFX1_2_3
Cluster-62068.20888	-2.3938	0.000421	0.038382	Ailuropoda melanoleuca uncharacterized LOC105235025 (LOC105235025)	--
Cluster-77665.0	-2.3976	0.000527	0.04543	Fc fragment of IgG binding protein (FCGBP)	FCGBP
Cluster-62068.117447	-2.3981	5.44E-05	0.0082991	BCL2-associated athanogene 5 (BAG5)	BAG5
Cluster-62068.146198	-2.4123	0.000533	0.045829	ATPase type 13A4 (ATP13A4)	ATP13A3_4_5
Cluster-62068.184060	-2.4166	0.000273	0.02807	Canis lupus familiaris solute carrier organic anion transporter family, member 1A2 (SLCO1A2)	SLCO1A2
Cluster-62068.55632	-2.4209	1.49E-08	9.88E-06	histone deacetylase 11 (HDAC11)	HDAC11
Cluster-62068.66595	-2.43	0.000187	0.021286	adenylate cyclase activating polypeptide 1 (pituitary) receptor type I (ADCYAP1R1)	ADCYAP1R1
Cluster-62068.106397	-2.4383	3.91E-07	0.00015455	CD44 molecule (CD44)4	CD44
Cluster-62068.67088	-2.4416	6.70E-05	0.0097972	unc-5 netrin receptor B (UNC5B)	UNC5
Cluster-62068.72317	-2.4523	0.00021	0.023187	transportin 3 (TNPO3)	TRPO3, MTR10
Cluster-62068.49238	-2.4542	0.000244	0.025954	calcium-sensing receptor (CASR)	CASR
Cluster-62068.167330	-2.4669	5.45E-07	0.00020941	Odobenus rosmarus divergens H-2 class II histocompatibility antigen, E-S beta chain-like (LOC101379947)	MHC2
Cluster-62068.34909	-2.4752	1.37E-08	9.19E-06	peptidyl arginine deiminase, type II (PADI2)	PADI2
Cluster-62068.192354	-2.4752	0.000305	0.030305	deleted in malignant brain tumors 1 protein (LOC101678849)	DMBT1
Cluster-62068.110999	-2.4768	1.12E-05	0.0023968	Odobenus rosmarus divergens claudin 10 (CLDN10)	CLDN
Cluster-62068.4273	-2.4853	3.53E-07	0.00014276	Ovis canadensis canadensis isolate 43U chromosome 1 sequence	--
Cluster-62068.114551	-2.4993	0.000302	0.030145	peptidase domain containing associated with muscle regeneration 1	PAMR1

				(PAMR1), transcript variant X7	
Cluster-62068.188760	-2.5036	0.00011	0.01436	Ursus maritimus ADP-ribosyltransferase 4 (ART4)	ART4
Cluster-62068.171737	-2.5168	0.000252	0.026575	ADP-ribosyltransferase 4 (ART4)	ART4, DO
Cluster-62068.130575	-2.5209	0.000523	0.045167	Ailuropoda melanoleuca transcription factor CP2 (TFCP2)	TFCP2
Cluster-62068.42468	-2.5315	0.000472	0.04184	arylsulfatase G (ARSG)	ARSG
Cluster-62068.187165	-2.5378	4.22E-06	0.0011067	Ovis canadensis canadensis isolate 43U chromosome 1 sequence	--
Cluster-62068.75398	-2.538	0.000472	0.04184	transmembrane 7 superfamily member 3 (TM7SF3)	TM7SF3
Cluster-62068.159003	-2.5477	0.000207	0.022983	chromosome 3 clone RP11-245G23	--
Cluster-62068.87022	-2.5666	0.000452	0.040591	Ovis canadensis canadensis isolate 43U chromosome 15 sequence	--
Cluster-62068.29469	-2.576	0.000209	0.023143	Ailuropoda melanoleuca collagen alpha-6(VI) chain (LOC100463941)	COL6A
Cluster-62068.105469	-2.5953	0.000374	0.035514	Canis familiaris chromosome 24	SIRPA_B1_G
Cluster-62068.65199	-2.5983	8.71E-05	0.011982	transmembrane protein 214 (TMEM214)	TMEM214
Cluster-62068.80124	-2.6129	8.85E-07	0.0003132	tudor domain containing 12 (TDRD12)	TDRD12
Cluster-62068.148702	-2.6346	0.000263	0.027363	armadillo repeat containing 9 (ARMC9)	ARMC9
Cluster-62068.161897	-2.6378	3.52E-05	0.0059419	Mustela vison clone I210 genomic sequence	--
Cluster-62068.21697	-2.6397	0.000227	0.024526	GATA binding protein 3 (GATA3)	GATA3
Cluster-62068.156680	-2.6405	0.000598	0.049811	Ovis canadensis canadensis isolate 43U chromosome 13 sequence	--
Cluster-62068.113550	-2.6543	0.000294	0.029554	protein tyrosine phosphatase (PTPRR)	PTPRR
Cluster-62068.648	-2.6582	4.24E-05	0.0068896	chromosome 3 clone RP11-543A18	--
Cluster-62068.118710	-2.674	0.000148	0.017869	KIAA0368 ortholog (KIAA0368)	ECM29
Cluster-62068.173547	-2.6746	0.000301	0.030086	UBX domain protein 10 (UBXN10)	UBXN10
Cluster-62068.11264	-2.6865	2.13E-05	0.0039649	glutamate-rich 3 (ERICH3)	ERICH3
Cluster-55981.1	-2.6911	0.000579	0.048665	SH2 domain containing 1B (SH2D1B)	SH2D1B
Cluster-62068.5821	-2.6924	0.000386	0.03625	Ovis canadensis canadensis isolate 43U chromosome 19 sequence	--

Cluster-62068.157005	-2.6957	5.87E-13	1.39E-09	kallikrein 1 (KLK1)	KLK1_2
Cluster-62068.58315	-2.6987	0.000396	0.036879	RPGRIP1-like (RPGRIP1L)	RPGRIP1L
Cluster-62068.40631	-2.7017	0.000544	0.046537	caspase recruitment domain family(CARD10)	CARD10
Cluster-62068.29538	-2.7028	1.57E-05	0.0031366	bone morphogenetic protein 8a (BMP8A)	BMP8
Cluster-62068.50867	-2.7059	0.000549	0.046767	zinc finger protein 496 (ZNF496)	ZNF496
Cluster-45687.0	-2.7099	0.000523	0.045167	WD repeat domain 88 (WDR88)	WDR88
Cluster-62068.157883	-2.7141	7.43E-05	0.010609	catenin (cadherin-associated protein) (CTNNA2)	CTNNA
Cluster-62068.168145	-2.72	0.0005	0.043711	solute carrier family 23 member 2 (LOC101693604)	SLC23A1_2, SVCT1_2
Cluster-62068.179121	-2.7211	0.000496	0.043498	armadillo repeat containing 6 (ARMC6)	ARMC6
Cluster-62068.163899	-2.7212	0.000313	0.03093	Canis lupus familiaris chromosome 7 open reading frame	C7H1orf27
Cluster-62068.7173	-2.725	8.36E-07	0.00029798	sialidase 2 (cytosolic sialidase) (NEU2)	NEU2
Cluster-62068.151925	-2.7301	0.000502	0.043814	Cbl proto-oncogene B, E3 ubiquitin protein ligase (CBLB)	ITGAM
Cluster-11502.0	-2.732	0.000481	0.0424	transmembrane protein 74 (TMEM74)	TMEM74
Cluster-62068.167891	-2.734	0.000103	0.013586	uncharacterized LOC106006249	--
Cluster-62068.148850	-2.7379	0.00014	0.017192	chromosome 5, BAC clone 7g12 (LBNL H126)	CXCL14, SCYB14
Cluster-62068.587	-2.7396	0.000467	0.041624	C-type lectin domain family 4 member G (LOC101689830)	--
Cluster-62068.161180	-2.7522	0.00043	0.039002	calcium responsive transcription factor (CARF)1	CARF
Cluster-62068.131714	-2.7528	0.000442	0.03994	zinc finger, MYM-type 2 (ZMYM2)	ZMYM2
Cluster-51444.0	-2.758	0.000402	0.037398	MACACA MULATTA BAC clone CH250-201N11 from chromosome 12	--
Cluster-62068.26977	-2.7597	0.000423	0.038553	Felis catus BAC clone FCAB-53H12 from chromosome unknown	--
Cluster-62068.178141	-2.7617	6.16E-08	3.34E-05	Sus scrofa clone CH242-189G19 protein kinase AMP-activated alpha 2 catalytic subunit (PRKAA2) gene	PRKAA, AMPK
Cluster-62068.98635	-2.7658	9.40E-05	0.012661	THO complex 1 (THOC1)	THOC1

Cluster-62068.33982	-2.7679	0.000416	0.038127	GTF2I repeat domain containing 1 (GTF2IRD1)	TFII-I, GTF2I
Cluster-46443.0	-2.7754	0.000386	0.036276	Ovis canadensis canadensis isolate 43U chromosome 3 sequence	--
Cluster-62068.16434	-2.7775	0.00036	0.03453	Pan troglodytes zinc finger protein 639 (ZNF639)	ZNF639
Cluster-62068.177931	-2.7831	0.000403	0.03741	myosin IIIB (MYO3B)	MYO3, DFNB30
Cluster-62068.38233	-2.7842	1.40E-07	6.74E-05	thyroglobulin (TG)	TG
Cluster-62068.79215	-2.7859	0.000364	0.03476	esterase D (ESD)	frmB, ESD, fghA
Cluster-62068.17157	-2.7886	0.000211	0.023223	Pig DNA sequence from clone CH242-512B14 on chromosome X	--
Cluster-62068.143866	-2.7915	0.000392	0.036655	jumonji domain containing 1C (JMJD1C)	JMJD1C
Cluster-62068.9159	-2.7977	0.000138	0.016987	glutamate-rich 3 (ERICH3)	ERICH3
Cluster-62068.3071	-2.8039	4.46E-05	0.007168	uncharacterized LOC106007422 (LOC106007422)	--
Cluster-62068.83711	-2.8066	1.16E-17	7.93E-14	palmitoyl-protein thioesterase 1 (PPT1)	PPT
Cluster-52261.0	-2.8129	0.000312	0.030824	uncharacterized LOC106005443 (LOC106005443)	--
Cluster-39582.0	-2.8134	8.99E-05	0.012297	SVOP-like (SVOPL)	SVOPL
Cluster-17836.0	-2.8179	0.000299	0.029928	natural cytotoxicity triggering receptor 1 (LOC101670751)	NCR1
Cluster-62068.94474	-2.8205	0.000223	0.024225	Odobenus rosmarus divergens integrin (ITGB4)	ITGB4
Cluster-62068.8779	-2.821	3.76E-06	0.0010059	Ailuropoda melanoleuca collagen alpha-6(VI) chain (LOC100463941)	COL6A
Cluster-62068.152097	-2.8212	0.000113	0.014618	Human DNA sequence from clone RP11-426L16 on chromosome 1	--
Cluster-62068.11471	-2.8233	0.00028	0.028572	glutaminase 2 (liver, mitochondrial) (GLS2)	GLS2
Cluster-62068.46334	-2.8271	0.000283	0.028729	Odobenus rosmarus divergens UV radiation resistance associated (UVRAG)	UVRAG
Cluster-31737.0	-2.829	0.00028	0.028537	Pig DNA sequence from clone CH242-244H5 on chromosome X	--
Cluster-62068.176015	-2.8308	0.000267	0.027655	microtubule-associated protein tau (MAPT)9	MAPT, TAU
Cluster-62068.25250	-2.8309	0.000283	0.028757	meiosis-specific nuclear structural 1 (MNS1)	MNS1
Cluster-62068.45237	-2.8316	0.000272	0.028043	thrombospondin 2 (THBS2)	THBS2S
Cluster-42250.2	-2.8316	0.000272	0.028043	Parascaris equorum genome assembly P_equorum ,scaffold PEQ_scaffold0015810	--

Cluster-62068.190191	-2.8333	0.000261	0.027133	Ursus maritimus NIMA-related kinase 5 (NEK5)	NEK
Cluster-62068.91041	-2.8357	0.000275	0.028203	Odobenus rosmarus divergens ubiquilin 1 (UBQLN1)	UBQLN, DSK2
Cluster-62068.176312	-2.8404	0.000257	0.026856	uridine phosphorylase 1 (UPP1)	udp, UPP
Cluster-62068.186723	-2.8434	0.000273	0.02807	Ovis canadensis canadensis isolate 43U chromosome 15 sequence	--
Cluster-62068.175595	-2.8504	3.92E-07	0.00015455	chromosome 5 clone RP11-158J3	--
Cluster-62068.27776	-2.8533	0.00024	0.025619	WW domain containing E3 ubiquitin protein ligase 2 (WWP2)	WWP2, AIP2
Cluster-62068.19273	-2.8535	0.000238	0.025409	coiled-coil domain containing 93 (CCDC93)	CCDC93
Cluster-62068.58539	-2.8564	0.000257	0.026856	Leptonychotes weddellii uncharacterized LOC102742436, ncRNA	BBC3, PUMA
Cluster-63313.0	-2.8602	2.84E-06	0.0008006	Neovison vison cytochrome P450 family 1 subfamily A polypeptide 1 (CYP1A1)	CYP1A1
Cluster-62068.169472	-2.8612	0.000148	0.017856	Ailuropoda melanoleuca cytochrome P450 4A6-like (LOC100465965)	--
Cluster-62068.190467	-2.8653	0.000261	0.027133	cat eye syndrome chromosome region (CECR1)	CECR1, ADA2
Cluster-62068.14994	-2.8675	0.000214	0.023475	Human DNA sequence from clone RP1-122O8 on chromosome 6q14.2-16.1	--
Cluster-62068.148085	-2.869	0.000212	0.023285	crystallin, lambda 1 (CRYL1)	CRYL1
Cluster-62068.43562	-2.872	0.000211	0.023223	uncharacterized LOC106007064 (LOC106007064)	--
Cluster-62068.21612	-2.8725	0.00022	0.024061	kinesin family member 6 (KIF6)	KIF6_9
Cluster-62068.5536	-2.8761	0.000137	0.016944	Bos taurus uncharacterized LOC101906240 (LOC101906240)8	--
Cluster-62068.105569	-2.882	0.000202	0.022625	A-Raf proto-oncogene, serine/threonine kinase (ARAF)	ARAF, ARAF1
Cluster-55370.0	-2.884	0.000197	0.022218	synaptonemal complex protein 2 (SYCP2)	SYCP2
Cluster-62068.144666	-2.886	0.00021	0.023187	Rhesus Macaque BAC CH250-4H19 complete sequence	UPK1
Cluster-62068.78004	-2.8876	0.000205	0.022796	Odobenus rosmarus divergens sterile alpha motif domain containing 14 (SAMD14)	SAMD14
Cluster-62068.149627	-2.8915	3.91E-05	0.0064187	solute carrier family 16 (SLC16A9)	SLC16A9
Cluster-62068.115377	-2.8915	0.000231	0.024923	glutamic-pyruvate transaminase (GPT)	GPT, ALT
Cluster-62068.32171	-2.8928	0.000193	0.021809	DNA cross-link repair 1A (DCLRE1A)	DCLRE1A,

					SNM1A
Cluster-62068.170868	-2.9016	1.59E-05	0.0031626	metastasis associated 1 family, member 3 (MTA3)	MTA3
Cluster-62068.132697	-2.9037	0.00018	0.020662	COMM domain containing 9 (COMMD9)	COMMD9
Cluster-62068.23491	-2.905	0.000196	0.022121	Ursus maritimus cell adhesion associated, oncogene regulated (CDON)	CDON
Cluster-62068.143543	-2.9075	7.33E-05	0.010513	nuclear factor of kappa light polypeptide gene enhancer in B-cells inhibitor, zeta (NFKBIZ)	NFKBIZ
Cluster-62068.180969	-2.9123	0.000169	0.0199	poly (ADP-ribose) polymerase family, member 3 (PARP3)	PARP
Cluster-62068.190702	-2.9169	0.000165	0.01955	zinc finger, SWIM-type containing 2 (ZSWIM2)	ZSWIM2
Cluster-62068.154653	-2.9181	0.000174	0.020215	alpha-kinase 1 (ALPK1)	ALPK1
Cluster-62068.117848	-2.9221	0.00017	0.019938	uncharacterized LOC101672653 (LOC101672653)	LOC101672653
Cluster-62068.50460	-2.9237	0.000178	0.020561	Odobenus rosmarus divergens myeloid/lymphoid or mixed-lineage leukemia	MLLT1_3, ENL, AF9
Cluster-62068.43915	-2.9267	0.000166	0.019606	BCL2-like 13 (apoptosis facilitator) (BCL2L13)	BCL2L13
Cluster-62068.159087	-2.9273	0.000168	0.019787	zinc finger, CCHC domain containing 6 (ZCCHC6)	TUT
Cluster-62068.140574	-2.9314	0.000149	0.017961	PDZ domain containing 3 (PDZD3)	PDZD3
Cluster-62068.44477	-2.9344	8.75E-05	0.012014	Odobenus rosmarus divergens diacylglycerol O-acyltransferase 2 (DGAT2)	DGAT2
Cluster-62068.135376	-2.9359	8.20E-05	0.011394	integrin(ITGA9)	ITGA9
Cluster-62068.141506	-2.9403	0.000142	0.017287	DLA class II histocompatibility antigen, DR-1 beta chain-like (LOC101670362)	--
Cluster-62068.155611	-2.9448	0.000141	0.017232	Canis lupus familiaris t-SNARE domain containing 1 (TSNARE1)	TSNARE1
Cluster-62068.64643	-2.945	0.000139	0.017135	cyclin D1 (CCND1)	CCND1
Cluster-69151.0	-2.9474	0.000148	0.017837	MACACA MULATTA BAC clone CH250-25B17 from chromosome 1	--
Cluster-62068.168513	-2.9483	0.000146	0.017645	nucleotide-binding oligomerization domain containing 2 (NOD2)	NOD2
Cluster-62068.182040	-2.9528	4.62E-05	0.0073249	carbonic anhydrase III (CA3)	E4.2.1.1
Cluster-62068.63255	-2.9539	2.66E-05	0.0047212	zinc finger, MYND-type containing 15 (ZMYND15)	ZMYND15

Cluster-62068.112123	-2.9561	0.000148	0.017837	Ursus maritimus KDEL (Lys-Asp-Glu-Leu) endoplasmic reticulum protein retention receptor 1 (KDELR1)	KDELR1
Cluster-62068.131645	-2.962	0.00014	0.017192	neuron navigator 1 (NAV1)	NAV1
Cluster-62068.3706	-2.9662	0.000155	0.018476	cell death-inducing DFFA-like effector c (CIDEC)	CIDEC
Cluster-62068.131109	-2.9674	0.000133	0.016552	inositol polyphosphate-5-phosphatase F (INPP5F)	INPP5F
Cluster-62068.165458	-2.9695	0.000133	0.016577	chromosome 19 clone CTB-60E11	--
Cluster-62068.154308	-2.9974	0.00011	0.014281	UBX domain protein 11 (UBXN11)	UBXN11
Cluster-62068.53754	-2.9986	0.000111	0.014459	period circadian clock 2 (PER2)	PER
Cluster-62068.24977	-2.9991	0.000116	0.014953	arachidonate 5-lipoxygenase (ALOX5)	ALOX5
Cluster-62068.155259	-3.0053	9.65E-05	0.012905	striatin, calmodulin binding protein (STRN)	STRN1_3_4
Cluster-62068.167704	-3.0087	9.92E-05	0.013212	chromosome 17, clone RP11-498C9	--
Cluster-62068.17796	-3.016	9.18E-05	0.012491	immunoglobulin superfamily (IGSF10)	IGSF10
Cluster-62068.84721	-3.0168	4.45E-05	0.0071655	transmembrane protein 214 (TMEM214)	TMEM214
Cluster-62068.87273	-3.02	9.36E-05	0.012627	WD repeat domain 6 (WDR6)	WDR6
Cluster-62068.8154	-3.0213	9.25E-05	0.012539	solute carrier family 35, member F3 (SLC35F3)	SLC35F3_4
Cluster-62068.129475	-3.0227	7.50E-07	0.00027253	chemokine (C-X-C motif) ligand 14 (CXCL14)	CXCL14, SCYB14
Cluster-62068.167826	-3.0331	8.19E-05	0.011394	carbohydrate (chondroitin 4) sulfotransferase 11 (CHST11)	CHST11
Cluster-62068.12630	-3.0435	9.33E-05	0.012607	solute carrier family 35 (UDP-GlcA/UDP-GalNAc transporter)	SLC35D
Cluster-62068.175393	-3.0552	7.34E-05	0.010513	Macaca mulatta BAC CH250-440C19	--
Cluster-24.0	-3.0593	7.66E-05	0.010827	Ovis canadensis canadensis isolate 43U chromosome 10 sequence	--
Cluster-62068.177104	-3.0648	6.75E-05	0.0098377	Ursus maritimus phosphatidylinositol-4-phosphate 5-kinase	PIP5K
Cluster-62068.158732	-3.0648	6.85E-05	0.0099565	Felis catus uncharacterized LOC102900733 (LOC102900733)	--
Cluster-62068.63553	-3.0676	7.57E-05	0.010753	STAM binding protein (STAMBP), RefSeqGene on chromosome 2	STAMBP
Cluster-62068.148552	-3.0716	6.41E-05	0.0094615	Leptonychotes weddellii integral membrane protein 2A (ITM2A)	ITM2A

Cluster-62068.78518	-3.0814	6.16E-05	0.0091658	secretion associated, Ras related GTPase 1B (SAR1B)	SAR1
Cluster-62068.78523	-3.0834	6.88E-05	0.0099711	obscurin-like 1 (OBSL1)	OBSL1
Cluster-62068.187704	-3.0904	6.12E-05	0.0091189	Felis catus FLA extended class II, class II, class III, proximal and central class I region genomic sequence	MHC2
Cluster-1089.0	-3.0988	2.53E-05	0.0045468	chromosome 3 clone CTC-501D11	CTC-501D11
Cluster-62068.98198	-3.1012	2.69E-05	0.0047629	ribosomal protein L3 (RPL3)	RP-L3e, RPL3
Cluster-62068.37541	-3.1061	1.94E-05	0.0037151	Pig DNA sequence from clone CH242-96E20 on chromosome X	--
Cluster-62068.9276	-3.1162	9.50E-06	0.0021217	sialidase 2 (cytosolic sialidase) (NEU2)	NEU2
Cluster-62068.134388	-3.1177	5.09E-05	0.0078748	unc-13 homolog D (C. elegans) (UNC13D)	UNC13D
Cluster-62068.27760	-3.118	5.76E-05	0.0087001	N-acetylneuraminate pyruvate lyase (dihydrodipicolinate synthase) (NPL)	E4.1.3.3, nanA, NPL
Cluster-62068.98329	-3.1233	5.26E-05	0.0080744	Canis lupus familiaris ecotropic viral integration site 5-like (EVI5L), transcript variant 1	EVI5L
Cluster-62068.52400	-3.1268	4.61E-05	0.0073094	IQ motif and Sec7 domain 2 (IQSEC2)	IQSEC
Cluster-62068.109202	-3.1294	5.18E-05	0.007983	solute carrier family 26 (anion exchanger), member 11 (SLC26A11)	SLC26A11
Cluster-62068.75360	-3.1331	5.66E-05	0.0085585	interferon, gamma-inducible protein 30 (IFI30)	IFI30, GILT
Cluster-62068.171169	-3.1341	4.48E-05	0.0071702	membrane metallo-endopeptidase-like 1 (MMEL1)	MMEL1
Cluster-62068.101885	-3.1405	4.86E-05	0.0076162	Panthera tigris altaica family with sequence similarity 192	FAM192A
Cluster-62068.121690	-3.1503	2.67E-07	0.00011437	chemokine (C-X-C motif) ligand 14 (CXCL14)	CXCL14
Cluster-62068.4980	-3.152	6.60E-06	0.0015845	Heterocephalus glaber 5-hydroxytryptamine (serotonin) receptor 1A, G protein-coupled (Htr1a)	--
Cluster-62068.47597	-3.1535	3.77E-05	0.0062357	Canis lupus familiaris sterile alpha motif and leucine zipper containing kinase AZK (ZAK)	ZAK, MLTK
Cluster-49238.1	-3.1537	1.87E-05	0.003594	uncharacterized LOC106004415 (LOC106004415)	--
Cluster-62068.182041	-3.154	9.83E-06	0.0021633	carbonic anhydrase III (CA3)	E4.2.1.1

Cluster-62068.156839	-3.158	5.32E-06	0.0013366	Ursus americanus clone UA-BM3-P1C09 microsatellite sequence	--
Cluster-62068.6184	-3.1596	4.78E-05	0.0075255	Canis lupus familiaris uncharacterized LOC102155802 (LOC102155802)	--
Cluster-62068.180381	-3.1755	3.33E-05	0.0057229	zinc finger protein 385C (ZNF385C)	ZNF385C
Cluster-62068.37565	-3.1806	3.29E-05	0.0056681	dihydrouridine synthase 2 (DUS2)	DUS2
Cluster-19684.0	-3.1822	3.48E-05	0.0058916	Ovis canadensis canadensis isolate 43U chromosome 19 sequence	--
Cluster-62068.61663	-3.1834	3.48E-05	0.0058916	bromodomain containing 1 (BRD1)	BRD1, BRPF2
Cluster-62068.22390	-3.1839	3.28E-05	0.0056581	ZFP91 zinc finger protein (ZFP91)	ZFP91
Cluster-62068.182451	-3.1891	4.02E-05	0.0065887	Ovis canadensis canadensis isolate 43U chromosome 8 sequence	--
Cluster-62068.133462	-3.1951	3.63E-05	0.0060791	Odobenus rosmarus divergens ubiquitin protein ligase E3B (UBE3B)	UBE3B
Cluster-62068.170901	-3.1998	2.75E-05	0.0048375	dynamin binding protein (DNMBP)	DNMBP
Cluster-62068.100561	-3.2038	4.16E-05	0.006782	paroxysmal nonkinesigenic dyskinesia (PNKD)	PNKD
Cluster-62068.159278	-3.2322	2.55E-05	0.0045779	ankyrin repeat domain 55 (ANKRD55)	ANKRD55
Cluster-13462.0	-3.2329	2.23E-05	0.0041034	uncharacterized LOC106006270 (LOC106006270)	--
Cluster-62068.173485	-3.2486	2.18E-05	0.0040493	zinc finger with KRAB and SCAN domains 2 (ZKSCAN2)	ZKSCAN
Cluster-62068.185643	-3.2571	2.07E-05	0.003894	Felis catus clone RP86-588L5	--
Cluster-34321.0	-3.2585	2.02E-05	0.0038178	Odobenus rosmarus divergens zinc finger, FYVE domain containing 28 (ZFYVE28)	ZFYVE28
Cluster-62068.123720	-3.2677	1.76E-05	0.0034239	polyamine modulated factor 1 binding protein 1 (PMFBP1)	DHX38, PRP16
Cluster-62068.117313	-3.2705	1.83E-05	0.0035412	Odobenus rosmarus divergens breast cancer metastasis suppressor 1 (BRMS1)	BRMS1
Cluster-62068.11039	-3.2708	1.95E-05	0.0037219	ATP-binding cassette, sub-family A (ABC1)	ABCA8
Cluster-62068.122361	-3.2731	1.69E-05	0.0033046	uncharacterized LOC106006249 (LOC106006249)	--
Cluster-62068.129060	-3.2762	1.81E-05	0.0035128	ATPase type 13A1 (ATP13A1)	ATP13A1
Cluster-62068.140791	-3.2856	7.29E-07	0.00026762	DAZ interacting zinc finger protein 1 (DZIP1)	CLDN
Cluster-62068.173363	-3.2925	3.54E-07	0.00014276	olfactomedin 4 (OLFM4)	OLFM4
Cluster-62068.55650	-3.2961	1.67E-05	0.0032754	cDNA, FLJ18301	--

Cluster-62068.20096	-3.3065	1.45E-05	0.0029447	Macaca mulatta neurogenic locus notch homolog protein 3-like (NOTCH3)	NOTCH3
Cluster-62068.39399	-3.3075	1.61E-05	0.0031874	Odobenus rosmarus divergens neuroblastoma breakpoint family member 6-like protein (LOC101363536)	--
Cluster-62068.3770	-3.3079	1.50E-05	0.0030116	sialic acid binding Ig-like lectin 1, sialoadhesin (SIGLEC1)	SN, SIGLEC1
Cluster-62068.184059	-3.3141	3.31E-07	0.0001363	solute carrier organic anion transporter family(SLCO1A2)	SLCO1A
Cluster-62068.31067	-3.3152	1.86E-05	0.003579	T-cell surface glycoprotein CD1a-like (LOC101690926)	CD1
Cluster-62068.133551	-3.3188	1.44E-05	0.0029263	25-hydroxycholesterol 7-alpha-hydroxylase (LOC101686726)	CYP7B
Cluster-62068.86722	-3.3238	1.25E-06	0.00041595	sialidase 2 (cytosolic sialidase) (NEU2)	NEU2_3_4
Cluster-62068.149222	-3.3241	1.62E-05	0.0031933	sperm flagellar 2 (SPEF2)	SPEF2
Cluster-62068.8785	-3.3254	1.35E-05	0.002798	Pan troglodytes BAC clone RP43-136P16 from chromosome 7	--
Cluster-62068.163163	-3.3288	1.23E-05	0.0025783	nicotinamide N-methyltransferase (NNMT)	NNMT
Cluster-29086.0	-3.3293	1.21E-05	0.0025377	membrane-spanning 4-domains, subfamily A (MS4A7)	MS4A7
Cluster-62068.75957	-3.3295	1.13E-05	0.0023984	Rho guanine nucleotide exchange factor (GEF) 17 (ARHGEF17)	GEF
Cluster-62068.116984	-3.3357	1.16E-05	0.0024563	Microcebus murinus histone deacetylase 7 (HDAC7)	HDAC7
Cluster-62068.138861	-3.3391	1.14E-05	0.0024284	pleckstrin homology domain containing, family G (with RhoGef domain) member 4B (PLEKHG4B)	PLEKHG4B
Cluster-62068.25115	-3.3408	1.25E-05	0.0025976	SWI5 recombination repair homolog (SWI5)	SWI5
Cluster-62068.133187	-3.3447	1.01E-05	0.0022133	cryptochrome circadian clock 2 (CRY2)	CRY
Cluster-62068.56484	-3.3583	1.01E-05	0.00221	tetratricopeptide repeat domain 39C (TTC39C)	TTC39C
Cluster-62068.16156	-3.3669	1.21E-05	0.0025338	nuclear factor of kappa light polypeptide gene enhancer in B-cells inhibitor (NFKBIZ)	NFKBIZ
Cluster-62068.16078	-3.3767	9.67E-06	0.0021415	MACACA MULATTA BAC clone CH250-58H12 from chromosome unknown	--
Cluster-62068.63823	-3.3776	1.03E-05	0.0022333	polypeptide N-acetylgalactosaminyltransferase 3 (GALNT3)	GALNT
Cluster-62068.106086	-3.3796	1.34E-06	0.00043554	lysosomal-associated membrane protein 2 (LAMP2)	LAMP2

Cluster-62068.69129	-3.3834	1.03E-05	0.0022333	intraflagellar transport 140 (IFT140)	IFT140
Cluster-62068.190003	-3.3843	8.63E-06	0.0019731	Canis familiaris chromosome 31	--
Cluster-62068.168416	-3.3894	9.38E-06	0.0021036	Ursus maritimus F-box protein 48 (FBXO48)	FBXO48
Cluster-62068.167164	-3.3914	9.56E-06	0.002126	KIAA1257 ortholog (KIAA1257)	ACAD9
Cluster-62068.131015	-3.397	9.81E-06	0.0021633	low density lipoprotein receptor-related protein 4 (LRP4)	LRP4
Cluster-62068.72106	-3.3971	8.63E-06	0.0019731	elaC ribonuclease Z 2 (ELAC2)	rnz
Cluster-62068.172726	-3.404	6.95E-06	0.0016588	sorting nexin 18 (SNX18)	SNX18
Cluster-62068.113017	-3.4063	3.95E-07	0.000155	kinesin light chain 2 (KLC2)	KLC2
Cluster-62068.7647	-3.4068	6.90E-06	0.0016534	noncompact myelin associated protein (NCMAP)	NCMAP
Cluster-62068.163671	-3.409	8.11E-07	0.00029132	zinc finger, SWIM-type containing 2 (ZSWIM2)	ZSWIM2
Cluster-62068.118935	-3.411	7.49E-06	0.0017539	chromosome transmission fidelity factor 8 (CHTF8)	UTP4, CIRH1A
Cluster-62068.146257	-3.4151	7.05E-06	0.0016744	coiled-coil domain containing 57 (CCDC57)	CCDC57
Cluster-62068.165297	-3.4154	7.49E-06	0.0017539	Ovis canadensis canadensis isolate 43U chromosome 4 sequence	--
Cluster-62068.167579	-3.4158	7.67E-06	0.0017909	chromosome 3 clone RP11-543A18	--
Cluster-62068.62384	-3.4162	7.33E-06	0.0017245	KIAA0100 ortholog (KIAA0100)	KIAA0100
Cluster-62068.34329	-3.4187	2.82E-06	0.00079711	carbonic anhydrase XII (CA12)	E4.2.1.1
Cluster-62068.150449	-3.4198	8.38E-06	0.0019296	gamma-glutamyltransferase 5 (GGT5)	GGT1_5
Cluster-62068.141324	-3.4255	5.94E-08	3.25E-05	Odobenus rosmarus divergens ankyrin 3, node of Ranvier (ankyrin G) (ANK3)	ANK
Cluster-8064.0	-3.4325	6.07E-06	0.0014687	Ovis canadensis canadensis isolate 43U chromosome 24 sequence	--
Cluster-62068.110942	-3.4411	5.01E-06	0.0012682	protein kinase N2 (PKN2)	PKN
Cluster-62068.17866	-3.4434	6.39E-06	0.0015386	absent in melanoma 1-like (AIM1L)	AIM1L
Cluster-62068.50242	-3.4488	6.02E-06	0.0014647	Odobenus rosmarus divergens CTP synthase 2 (CTPS2)	pyrG, CTPS
Cluster-62068.83805	-3.4608	4.66E-06	0.0012009	ankyrin 1, erythrocytic (ANK1)	ANK
Cluster-62068.121485	-3.4625	5.74E-06	0.0014192	Canis lupus familiaris c-abl oncogene 2, non-receptor tyrosine kinase (ABL2)	ABL2

Cluster-62068.64302	-3.4701	3.23E-06	0.00089963	Balaenoptera acutorostrata scammoni RAS p21 protein activator 4 (RASA4)	RASA4, CAPRI
Cluster-62068.30765	-3.4742	4.69E-06	0.001206	tetratricopeptide repeat domain 23-like (TTC23L)	--
Cluster-62068.108471	-3.4789	5.61E-07	0.0002141	KRI1 homolog (KRI1)	KRI1
Cluster-62068.65739	-3.4799	4.73E-06	0.0012132	intraflagellar transport 27 (IFT27), RefSeqGene on chromosome 22	IFT27
Cluster-55787.0	-3.4864	3.98E-06	0.0010546	solute carrier family 23 member 2 (LOC101693604)	--
Cluster-62068.132728	-3.4989	3.26E-06	0.00090435	leukemia inhibitory factor receptor alpha (LIFR)	LIFR
Cluster-62068.82584	-3.5115	3.44E-06	0.00094258	Leptonychotes weddellii plectin (PLEC)	PLEC
Cluster-62068.185925	-3.539	2.71E-06	0.00076878	lipoma HMGIC fusion partner-like 3 (LHFPL3)	LHFPL3
Cluster-62068.44476	-3.5514	1.04E-06	0.0003573	diacylglycerol O-acyltransferase 2 (DGAT2)	DGAT2
Cluster-62068.66766	-3.5577	2.24E-06	0.0006552	GDNF-inducible zinc finger protein 1 (GZF1)	GZF1
Cluster-62068.142998	-3.5703	2.25E-06	0.00065786	Equus caballus beta-1,4-N-acetyl-galactosaminyl transferase 4 (B4GALNT4)	B4GALNT4
Cluster-62068.147149	-3.5864	1.64E-06	0.00050513	MDM2 proto-oncogene, E3 ubiquitin protein ligase (MDM2)	MDM2
Cluster-45655.0	-3.587	2.14E-06	0.00063886	aldehyde oxidase 4-like (LOC101693479)	AOX
Cluster-62068.28522	-3.5945	1.63E-06	0.0005047	Odobenus rosmarus divergens potassium channel, voltage gated modifier subfamily G, member 3 (KCNG3)	KCNG3
Cluster-62068.81754	-3.5953	5.83E-10	6.04E-07	isoamyl acetate-hydrolyzing esterase 1 homolog (IAH1)	IAH1
Cluster-62068.173980	-3.6033	2.42E-06	0.00070011	MIS18 binding protein 1 (MIS18BP1)	MIS18BP1
Cluster-62068.76362	-3.608	2.26E-06	0.00065798	uncharacterized LOC101694508 (LOC101694508)	--
Cluster-62068.95618	-3.6126	1.64E-06	0.00050513	aryl hydrocarbon receptor nuclear translocator (ARNT)	ARNT
Cluster-62068.157479	-3.6181	2.22E-06	0.00065097	calcium/calmodulin-dependent protein kinase IG (CAMK1G)	CAMK1
Cluster-62068.185158	-3.6231	2.08E-06	0.00062478	Odobenus rosmarus divergens zinc finger protein 704 (ZNF704)	ZNF704
Cluster-62068.122995	-3.6287	1.76E-06	0.00053551	Ursus maritimus kelch domain containing 1 (KLHDC1)	KLHDC1
Cluster-62068.47429	-3.6365	1.23E-06	0.00041119	regucalcin (RGN)	gnl, RGN
Cluster-62068.144284	-3.6449	1.45E-06	0.00046487	Ursus maritimus TM2 domain containing 2 (TM2D2)	TM2D2
Cluster-62068.189136	-3.6476	1.25E-06	0.00041401	deleted in malignant brain tumors 1 protein-like (LOC106004722)	DMBT1

Cluster-62068.33067	-3.6527	1.28E-06	0.00041962	zinc finger, CCHC domain containing 14 (ZCCHC14)	ZCCHC14
Cluster-62068.51610	-3.6529	9.52E-07	0.00033187	Equus przewalskii trinucleotide repeat containing 6C (TNRC6C)	TNRC6, GW182
Cluster-62068.13116	-3.654	1.13E-06	0.00038497	spermatogenesis associated 7 (SPATA7)	SPATA7
Cluster-62068.130404	-3.654	9.88E-07	0.00034198	ankyrin repeat domain 52 (ANKRD52)	ANKRD52
Cluster-62068.24236	-3.661	1.47E-06	0.00046644	neurotrophic tyrosine kinase, receptor (NTRK3)	NTRK3
Cluster-62068.132113	-3.6644	1.26E-06	0.00041641	programmed cell death 5 (PDCD5)	PDCD5, TFAR19
Cluster-62068.83683	-3.6773	1.11E-06	0.00038164	extended synaptotagmin-like protein 1 (ESYT1)	ESYT1
Cluster-62068.32533	-3.6802	1.31E-06	0.0004278	chordin-like 2 (CHRDL2)	CHRDL2
Cluster-62068.31287	-3.6807	9.65E-07	0.00033484	uncharacterized LOC106007196 (LOC106007196)	--
Cluster-62068.174256	-3.6817	1.31E-06	0.00042712	Human DNA sequence from clone RP11-4K3 on chromosome 1	--
Cluster-62068.154349	-3.6841	8.24E-07	0.00029443	pyruvate dehydrogenase kinase (PDK4)	PDK2_3_4
Cluster-62068.8878	-3.689	1.47E-14	5.05E-11	mucin 13, cell surface associated (MUC13)	MUC13
Cluster-62068.142960	-3.6948	8.81E-07	0.00031232	centrosomal protein 128kDa (CEP128)0	CEP128
Cluster-62068.68626	-3.6957	1.24E-06	0.00041401	Odobenus rosmarus divergens histone deacetylase 5 (HDAC5)	HDAC4_5
Cluster-62068.55392	-3.7137	5.77E-07	0.00021972	SAGA-associated factor 29 homolog (LOC101684788)	--
Cluster-62068.55151	-3.7138	9.97E-07	0.00034437	hydroxy-delta-5-steroid dehydrogenase, 3 beta- and steroid delta-isomerase 7 (HSD3B7)	HSD3B7
Cluster-62068.83348	-3.7159	8.18E-07	0.00029304	glycosylated lysosomal membrane protein (GLMP)	GLMP
Cluster-62068.82182	-3.7288	6.07E-07	0.00022935	ADP-ribosylation factor-like 4A (ARL4A)	ARL4
Cluster-62068.149099	-3.7349	7.50E-07	0.00027253	Ailuropoda melanoleuca pogo transposable element with ZNF domain (POGZ)	POGZ
Cluster-62068.104460	-3.7394	6.77E-07	0.00025261	ATPase type 13A2 (ATP13A2)	ATP13A2
Cluster-62068.78889	-3.7423	5.43E-07	0.00020905	katanin p80 (WD repeat containing) subunit B 1 (KATNB1)	KATNB1
Cluster-62068.86727	-3.7552	6.14E-07	0.00023159	histone deacetylase 3 (HDAC3)	HDAC3
Cluster-62068.65240	-3.7801	3.54E-07	0.00014276	structural maintenance of chromosomes 5 (SMC5)	SMC5

Cluster-62068.53985	-3.8384	2.14E-07	9.53E-05	caspase 8, apoptosis-related cysteine peptidase (CASP8)	CASP8
Cluster-62068.134410	-3.8418	1.21E-14	4.34E-11	uncharacterized LOC101694508 (LOC101694508)	LOC101694508
Cluster-62068.39695	-3.8511	2.35E-07	0.00010236	ATP-binding cassette, sub-family A (ABC1) (ABCA5)	ABCA5
Cluster-62068.35375	-3.8512	3.25E-07	0.00013418	Ursus maritimus phosphoinositide kinase, FYVE finger containing (PIKFYVE)	PIKFYVE, FAB1
Cluster-62068.79235	-3.8518	2.35E-07	0.00010236	tripartite motif containing 3 (TRIM3)	TRIM2_3
Cluster-62068.119217	-3.8586	3.81E-07	0.00015097	TBC1 (tre-2/USP6, BUB2, cdc16) domain family(TBC1D1)	TBC1D1
Cluster-62068.86581	-3.8637	2.49E-07	0.00010774	lysyl oxidase-like 2 (LOXL2)	LOXL2_3_4
Cluster-62068.116879	-3.8665	2.68E-07	0.00011446	protein-O-mannosyltransferase 2 (POMT2)	POMT
Cluster-62068.71	-3.8718	2.16E-07	9.61E-05	ELOVL fatty acid elongase 2 (ELOVL2)	ELOVL2
Cluster-62068.152605	-3.8822	1.34E-07	6.48E-05	serine/threonine-protein phosphatase 4 regulatory subunit 1-like (LOC101690113)	PPP4R1
Cluster-62068.44025	-3.8911	1.87E-07	8.71E-05	chromosome unknown open reading frame, human C9orf64 (LOC101670215)	--
Cluster-62068.128949	-3.8984	2.19E-07	9.68E-05	Ailuropoda melanoleuca peptidylprolyl isomerase (cyclophilin)-like 6 (PPIL6)	PPIL6
Cluster-62068.52468	-3.9074	1.12E-08	7.75E-06	transmembrane protein 138 (TMEM138)	TMEM138
Cluster-62068.92742	-3.9264	9.01E-08	4.65E-05	RUN and SH3 domain containing 2 (RUSC2)	RUSC2
Cluster-62068.60160	-3.9322	2.44E-11	3.79E-08	translocase of inner mitochondrial membrane 50 homolog (TIMM50)	TIM50
Cluster-62068.140387	-3.9333	1.53E-07	7.23E-05	Ovis canadensis canadensis isolate 43U chromosome 14 sequence	--
Cluster-62068.173794	-3.9453	1.24E-07	6.12E-05	Rho GTPase activating protein 6 (ARHGAP6)	ARHGAP6
Cluster-62068.74994	-3.9502	1.16E-07	5.81E-05	thrombospondin 1 (THBS1)	THBS1
Cluster-62068.148743	-3.9504	8.54E-08	4.42E-05	zinc finger protein OZF-like (LOC101692271)	KRAB
Cluster-62068.106215	-3.951	8.00E-08	4.21E-05	adenylate cyclase 7 (ADCY7)	ADCY7
Cluster-62068.87324	-3.9633	6.29E-08	3.39E-05	polo-like kinase 2 (PLK2)	PLK2
Cluster-62068.60592	-3.9701	1.38E-07	6.65E-05	double C2-like domain-containing protein gamma (LOC101677986)	NUDT8

Cluster-62068.115391	-3.9754	7.50E-08	3.98E-05	integrin, alpha 5 (fibronectin receptor, alpha polypeptide) (ITGA5)	ITGA5
Cluster-62068.117903	-3.9776	9.15E-08	4.71E-05	abhydrolase domain containing 16A (ABHD16A)	ABHD16A
Cluster-62068.12142	-3.9823	6.79E-08	3.64E-05	WD repeat domain 17 (WDR17)	WDR17
Cluster-62068.108747	-4.0087	5.23E-08	2.89E-05	transmembrane protein 39B (TMEM39B)	TMEM39B
Cluster-35405.0	-4.0159	3.86E-08	2.23E-05	DTW domain containing 2 (DTWD2)	DTWD2
Cluster-62068.121908	-4.0259	6.01E-08	3.28E-05	Aotus nancymaae TEA domain family member 2 (TEAD2)	TEAD
Cluster-62068.6499	-4.0276	4.10E-08	2.35E-05	Canis Familiaris chromosome 6	--
Cluster-62068.110872	-4.0525	5.14E-08	2.86E-05	superkiller viralicidic activity 2-like (SKIV2L)	SKI2, SKIV2L
Cluster-62068.7627	-4.0674	2.94E-08	1.74E-05	anoctamin 9 (ANO9)	ANO9, TMEM16J
Cluster-62068.126986	-4.0874	2.21E-08	1.37E-05	RAS p21 protein activator 3 (RASA3)	RASA3
Cluster-62068.34939	-4.0911	2.07E-08	1.33E-05	whirlin (LOC101691120)	--
Cluster-62068.75920	-4.1003	2.80E-08	1.69E-05	PDZ domain containing ring finger 3 (PDZRN3)	PDZRN3_4, LNX3_4
Cluster-62068.191882	-4.1034	1.68E-08	1.11E-05	uncharacterized LOC101688371 (LOC101688371)	--
Cluster-62068.76722	-4.1256	3.71E-08	2.15E-05	ring finger protein 223 (RNF223)	RNF223
Cluster-62068.139898	-4.127	1.45E-08	9.67E-06	double C2-like domain-containing protein gamma (LOC101677986)	NUDT8
Cluster-62068.123130	-4.1312	2.97E-08	1.75E-05	proline-rich transmembrane protein 2 (PRRT2)	PRRT2
Cluster-62068.118430	-4.1324	1.32E-08	8.89E-06	urocortin (UCN)	MPV17
Cluster-62068.161689	-4.1347	2.55E-08	1.55E-05	Ovis canadensis canadensis isolate 43U chromosome 3 sequence	--
Cluster-62068.74231	-4.1484	1.17E-08	8.06E-06	tetratricopeptide repeat domain 19 (TTC19)	TTC19
Cluster-62068.143818	-4.1564	1.11E-09	1.06E-06	choline kinase alpha (CHKA)	CHK
Cluster-62068.36733	-4.1592	1.31E-08	8.89E-06	Ursus maritimus spermatogenesis associated 6-like (SPATA6L)	SPATA6L
Cluster-62068.21842	-4.169	1.21E-08	8.29E-06	chromosome 17, clone RP11-357H14	--
Cluster-62068.103622	-4.1705	1.53E-08	1.01E-05	TBC1 domain family, member 16 (TBC1D16)	TBC1D16

Cluster-62068.74715	-4.172	4.27E-09	3.51E-06	regulator of chromosome condensation (RCC1) and BTB (POZ) domain containing protein 1 (RCBTB1)	RCBTB
Cluster-62068.46864	-4.182	7.94E-09	5.81E-06	solute carrier family 38, member 8 (SLC38A8)	SLC38A7_8
Cluster-62068.161438	-4.1864	9.21E-09	6.53E-06	lipin 3 (LPIN3)	LPIN
Cluster-62068.142296	-4.1877	6.78E-09	5.11E-06	leucine-rich repeats and calponin homology (CH) domain containing 3 (LRCH3)	LRCH3
Cluster-62068.93946	-4.1971	1.37E-08	9.19E-06	large tumor suppressor kinase 2 (LATS2)	LATS1_2, Wts
Cluster-62068.179776	-4.2085	7.17E-09	5.35E-06	mitochondrial translational release factor 1 (MTRF1)	prfA, MTRF1, MRF1
Cluster-62068.175538	-4.2116	6.24E-09	4.81E-06	Pan troglodytes BAC clone CH251-696K14 from chromosome 11	--
Cluster-62068.80781	-4.2275	7.49E-09	5.56E-06	histone deacetylase 10 (HDAC10)	HDAC10
Cluster-62068.138194	-4.2355	1.90E-08	1.23E-05	trafficking protein particle complex 9 (TRAPPC9), RefSeqGene on chromosome 8	TRAPPC9
Cluster-62068.66149	-4.2479	5.11E-09	4.16E-06	uncharacterized LOC101683508 (LOC101683508)	--
Cluster-62068.146858	-4.2705	9.11E-09	6.49E-06	growth regulation by estrogen in breast cancer-like (GREB1L)	GREB1L
Cluster-62068.83712	-4.2743	5.82E-09	4.53E-06	mitogen-activated protein kinase kinase kinase 9 (MAP3K9)	MAP3K9
Cluster-62068.90491	-4.2764	8.38E-09	6.09E-06	EPH receptor B6 (EPHB6)	EPHB6
Cluster-62068.75719	-4.2799	3.88E-09	3.23E-06	Leptonychotes weddellii peripheral myelin protein 22 (PMP22)	PMP22
Cluster-62068.124330	-4.2944	9.31E-09	6.54E-06	bleomycin hydrolase (BLMH)	pepC
Cluster-62068.131407	-4.3201	3.31E-09	2.82E-06	Ovis canadensis canadensis isolate 43U chromosome 7 sequence	DHRS4
Cluster-62068.173753	-4.3206	2.50E-09	2.17E-06	WSC domain-containing protein 1-like (LOC101683385)	--
Cluster-62068.86895	-4.3287	3.37E-09	2.85E-06	MON1 secretory trafficking family member B (MON1B)	MON1B
Cluster-62068.153608	-4.3397	1.77E-09	1.62E-06	Ovis canadensis canadensis isolate 43U chromosome 5 sequence	--
Cluster-62068.43297	-4.3507	1.61E-09	1.49E-06	Leptonychotes weddellii zinc finger, DBF-type containing 2 (ZDBF2)	ZDBF2
Cluster-62068.102387	-4.3786	1.92E-09	1.72E-06	mitochondrial translational initiation factor 2 (MTIF2)	infB, MTIF2

Cluster-62068.70627	-4.4052	7.23E-10	7.18E-07	Felis catus ADP-ribosylation factor related protein 1 (ARFRP1)	ARFRP1
Cluster-62068.117871	-4.4384	6.90E-10	6.95E-07	HECT, UBA and WWE domain containing 1, E3 ubiquitin protein ligase (HUWE1)	HUWE1, MULE
Cluster-62068.127344	-4.4816	2.81E-10	3.09E-07	nuclear export mediator factor (NEMF)	NEMF
Cluster-62068.71904	-4.4913	6.68E-10	6.82E-07	forkhead box P1 (FOXP1)	FOXP
Cluster-62068.145686	-4.499	2.60E-10	2.95E-07	v-maf avian musculoaponeurotic fibrosarcoma oncogene homolog F (MAFF)	MAFF_G_K
Cluster-62068.69045	-4.5071	2.77E-10	3.07E-07	Odobenus rosmarus divergens zinc finger, BED-type containing 3 (ZBED3)	ZBED3
Cluster-62068.134672	-4.5326	4.53E-10	4.72E-07	sparc/osteonectin, cwcv and kazal-like domains proteoglycan (testican) 2 (SPOCK2)	SPOCK
Cluster-62068.136063	-4.5891	8.53E-10	8.41E-07	aquaporin 10 (AQP10)	AQP10
Cluster-62068.95725	-4.5985	6.82E-11	9.35E-08	flightless I actin binding protein (FLII)	FLII
Cluster-62068.107969	-4.6142	6.10E-11	8.52E-08	ectonucleotide pyrophosphatase/phosphodiesterase 3 (ENPP3)	ENPP1_3
Cluster-62068.103269	-4.6805	1.91E-11	3.09E-08	laminin, alpha 5 (LAMA5)	LAMA3_5
Cluster-62068.100927	-4.6807	6.35E-11	8.80E-08	actin filament associated protein 1 (AFAP1)	AFAP1
Cluster-62068.44460	-4.6868	2.45E-11	3.79E-08	growth regulation by estrogen in breast cancer-like (GREB1L)	GREB1L
Cluster-62068.96424	-4.7362	4.00E-11	5.88E-08	calcium binding protein 39-like (CAB39L)	SETDB2
Cluster-62068.122576	-4.7548	1.42E-10	1.77E-07	ly-6/neurotoxin-like protein 1 (LOC101676258)	--
Cluster-62068.102833	-4.7702	4.51E-11	6.50E-08	sushi domain containing 6 (SUSD6)	SUSD6
Cluster-62068.78683	-4.7948	7.78E-12	1.42E-08	lymphotoxin beta receptor (TNFR superfamily, member 3) (LTBR)	TNFRSF3, LTBR
Cluster-62068.153572	-4.8055	6.37E-12	1.21E-08	Ailuropoda melanoleuca uncharacterized LOC105241397 (LOC105241397)	LOC105241397
Cluster-62068.142358	-4.8283	9.69E-12	1.70E-08	Odobenus rosmarus divergens ribonuclease P/MRP 30kDa subunit (RPP30)	RPP1, RPP30
Cluster-62068.117457	-4.8305	2.42E-11	3.79E-08	lon peptidase 2, peroxisomal (LONP2)	lon
Cluster-62068.38701	-4.881	2.28E-12	4.55E-09	zinc finger, MYM-type 6 (ZMYM6)	ZMYM6
Cluster-62068.145617	-4.9079	3.70E-12	7.30E-09	Ailuropoda melanoleuca ER degradation enhancer, mannosidase alpha-like 1 (EDEM1)	EDEM1

Cluster-62068.148818	-4.9324	1.06E-12	2.30E-09	B-cell CLL/lymphoma 9 (BCL9)	BCL9
Cluster-62068.136993	-4.9486	1.17E-11	2.03E-08	jumonji domain containing 6 (JMJD6)	JMJD6
Cluster-62068.79339	-4.953	1.35E-11	2.31E-08	pleckstrin and Sec7 domain containing 3 (PSD3)3	PSD
Cluster-62068.117974	-4.9746	9.97E-13	2.21E-09	acid phosphatase 2, lysosomal (ACP2)	ACP2
Cluster-62068.80034	-5.1559	3.72E-14	1.21E-10	UbiA prenyltransferase domain containing 1 (UBIAD1)	UBIAD1
Cluster-62068.69571	-5.1568	3.87E-14	1.21E-10	trafficking protein particle complex 2 (TRAPPC2)	TRAPPC2
Cluster-62068.90195	-5.1568	6.74E-13	1.57E-09	mitogen-activated protein kinase kinase kinase 1, E3 ubiquitin protein ligase (MAP3K1)	MAP3K1, MEKK1
Cluster-62068.185039	-5.1588	2.19E-13	5.84E-10	5-hydroxytryptamine (serotonin) receptor 1A, G protein-coupled (HTR1A)	HTR1
Cluster-62068.89354	-5.159	3.67E-14	1.21E-10	mediator complex subunit 10 (MED10)	MED10, NUT2
Cluster-62068.149651	-5.1935	4.18E-14	1.25E-10	RAS p21 protein activator 2 (RASA2)	RASA2, GAP1M
Cluster-62068.99877	-5.2119	7.18E-13	1.64E-09	polypyrimidine tract binding protein 1 (PTBP1)	PTBP1, PTB
Cluster-62068.99323	-5.2223	3.76E-15	1.64E-11	ATPase, H+ transporting, lysosomal 70kDa, V1 subunit A (ATP6V1A)	ATPeV1A, ATP6A
Cluster-62068.77187	-5.2425	1.40E-14	4.91E-11	Canis familiaris TCTA gene, AMT gene, NICN1 gene, DAG1 gene and BSN gene	--
Cluster-62068.82680	-5.2523	3.07E-13	7.76E-10	HECT domain containing E3 ubiquitin protein ligase 1 (HECTD1)	HECTD1
Cluster-62068.90540	-5.355	6.36E-15	2.61E-11	WD repeat and FYVE domain containing 2 (WDFY2)	WDFY2
Cluster-62068.85832	-5.4227	2.19E-15	1.05E-11	interleukin 13 receptor, alpha 1 (IL13RA1)	IL13RA1
Cluster-62068.51913	-5.4595	2.31E-15	1.07E-11	tRNA methyltransferase 2 homolog A (TRMT2A)	TRMT2A
Cluster-62068.75652	-5.4893	2.38E-16	1.27E-12	mediator complex subunit 11 (MED11)	MED11
Cluster-62068.60273	-5.5757	1.47E-16	8.13E-13	ras responsive element binding protein 1 (RREB1)	RREB1
Cluster-62068.113249	-5.6508	2.77E-17	1.66E-13	ArfGAP with GTPase domain, ankyrin repeat and PH domain 1 (AGAP1)	PINK1
Cluster-62068.62596	-5.8227	6.70E-19	5.68E-15	ZXD family zinc finger C (ZXDC)	ZXDC
Cluster-62068.125746	-6.0343	6.47E-20	6.66E-16	immunoglobulin-like variable motif containing (BIVM)	ERCC5, XPG,

					RAD2
Cluster-62068.122018	-6.037	1.99E-19	1.91E-15	uncharacterized LOC101694508 (LOC101694508)	LOC101694508
Cluster-62068.76334	-6.5331	3.17E-34	1.52E-29	NEDD4 binding protein 1 (N4BP1)	N4BP1
Cluster-62068.116422	-7.0783	8.76E-31	2.52E-26	exportin 5 (XPO5)	XPO5
Cluster-62068.77633	-7.7671	3.41E-37	2.46E-32	TEA domain family member 3 (TEAD3)	TEAD

Table S2. Primers used for Q-PCR.

Gene	Rrimers	PCR product (bp)
EGF	F: CCG TGC TTT GGT TTC CAG TG<br>R:CCA GAT CCA CCA CCC CAA AA	283
HBEGF	F: AGA AGC CCC ACG ATG ACA AG<br>R: CCT CCC AGT GGA AAA TCG CT	105
EGFR	F: ACA CAG TCT TTC GGC TCT GG<br>R: GTT TTC TCT TGC GGT CGT CG	267
ER	F: GCC ATT TGT TCG CTT GCT CA<br>R: ATC TTG GGC TCT CCA ACA CG	206
IGF1	F: CTC GTC CAC AAT GCC TGT CT<br>R: GCT GAA CTG GTG GAT GCT CT	117
LIF	F: CGT CCG GCA TGG AGT GTA AA<br>R: ATC TTC CTT CTC GGT GGT GC	293
FKBP4	F: ACA GTA TCG GTG GAG TTG AAA A<br>R: ATT CCA TGC TGT GGG ACT GG	194
ODC	F: CAG GGT ATT GGG CTG GTG AG<br>R: ATG GTG GTA ATT CTG CGG GG	201

ASMT	F: TCCTGTGCTGTATCCCAACCT<br>R: CCCTATGCTGCCAGAAATCC	119
PDE11	F: CAAAGGGATGCTGTGGACTG<br>R: TGTGAAACTGAAGCCGATGC	217
MCM2	F: TTGCCAGTATTGCTCCTTCC<br>R: TTGATGTCACCTCGCACCTT	121
PMP22	F: TATGGCTTTGCCTACATCCTG<br>R: TGGTATGGTTTGAGTTTGGGTT	210
PIK3C2	F: CCAAGCGGGAACTCAAGAA<br>R: GAACCTGTAATCATCAGCACCA	219
SLC4A8	F: CCGTGCCAATCCGTTAAG<br>R: AAGAGAGACAAGAAGGGTAGG	107
CD1	F: GCTTGATATTGTTGGCCATAGTAG<br>R: AGGCTTCTGGGTGCTTCTTC	146
GALNT	F: ACCCTTCCAATACCAACGACT<br>R: ATACTTTCTACCTCCATTCACCCT	107
VEGFA	F: CAG GAA GAG CGG GGG CT<br>R: GGA GGA GGG ACC TGG GG	101
cmyc	F: AGT GCT TTG GAG TAT CCT TTT CA<br>R: CAT GGT CAC CCC ACC TTA CC	243
PR	F: CTA CCT GTG TGG ACG AAC CC<br>R: CCT AAG GTC TCC CTG ACC GA	121

PRLR	F: TTGCTCTAACCCGAGACTGG<br>R: TTGTTTTGCTGTTAGACTGCGT	128
LAMA3	F: GAAGGACAAGAAGGACGAAGAG<br>R: AACCACAAGTGGCAGGTAAAA	180
GAPDH	F: TAGTGTCCAGGGTGGGTGTT<br>R: CTGTGCTGTTCGCTGGTTTA	162

PPI network

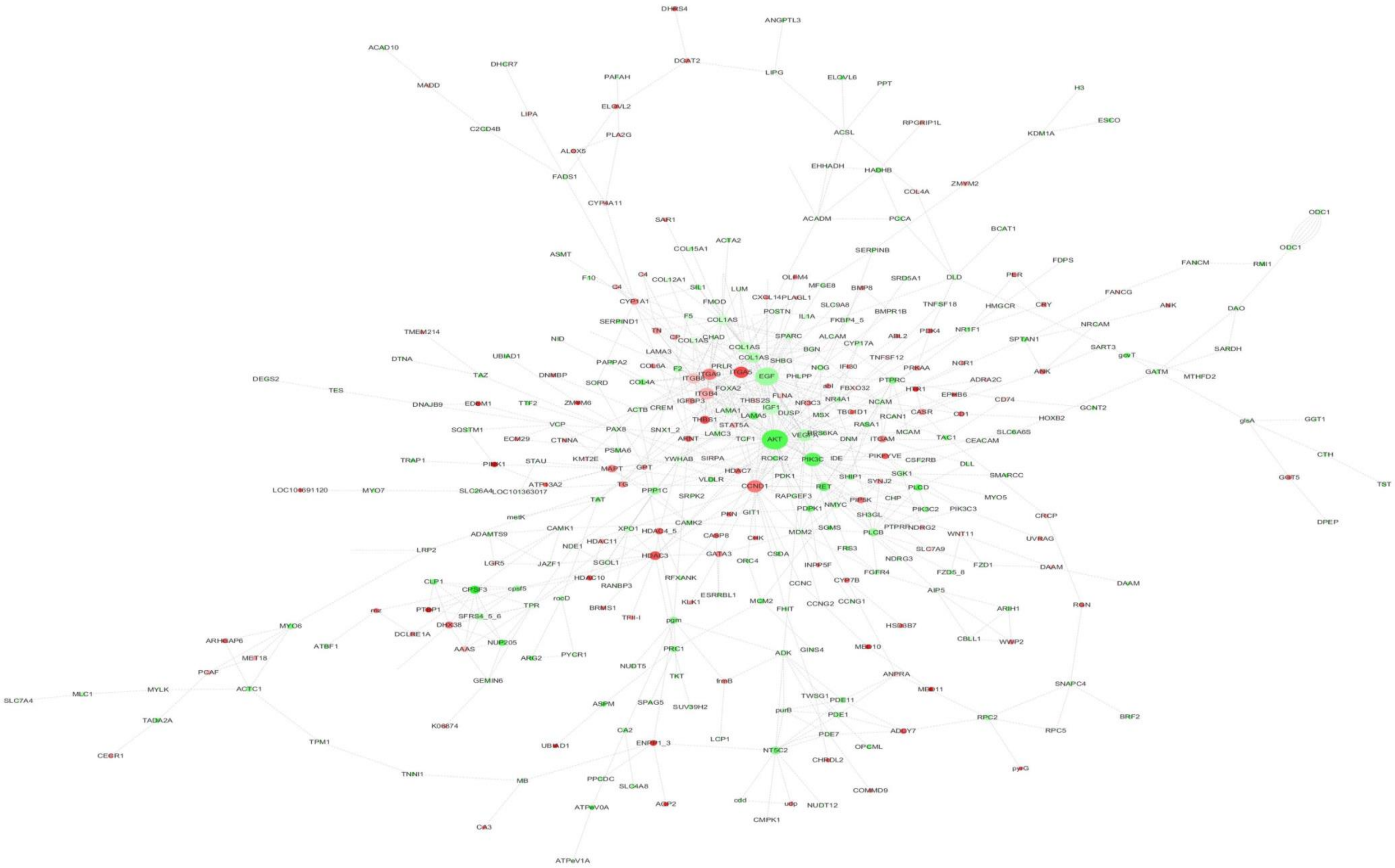
DHRS4
ANGPTL3
ACAD10
DHCR7
DGAT2
LIPG
ELOVL6
PPT
MADD
PAFAH
ELOVL2
H3
LIPA
RPGRIP1L
ESCO
C2CD4B
PLA2G
ACSL
KDM1A
ALOX5
EHHADH
HADHB
FADS1
ZMYM2
COL4A
CYP4A11
SAR1
ACADM
PCCA
ODC1
BCAT1
ACTA2
COL15A1
SERPINB
ODC1
ASMT
FDPS
FANCM
RMI1
C4
OLFM4
DLD
F10
COL12A1
MFGE8
SRD5A1
PBR
C4
SIL1
LUM
BMP8
FANCG
CYP1A1
FMOD
CXCL14
PLAGL1
SLC9A8
TNFSF18
HMGCR
CRY
ANK
DAO
POSTN
BMPR1B
SERPIND1
F5
COL1AS
IL1A
FKBP4_5
NRCAM
TMEM214
TN
CP
CHAD
SPARC
ALCAM
ABL2
PDK4
NR1F1
SPTAN1
NID
COL1AS
CYP17A
COL1AS
BGN
SART3
SARDH
UBIAD1
LAMA3
COL1AS
SHBG
TNFSF12
NGR1
DTNA
PAPPA2
COL6A
PRLR
ITGA6
NOG
IFI30
PRKAA
ANK
GATM
MTHFD2
TAZ
DNMBP
ITGA9
F2
ITGB8
EGF
PHLPP
PTPRC
ADRA2C
DEGS2
SORD
COL4A
FOXA2
abl
FBXO32
TES
ITGB4
HTR1
EPHB6
CD74
DNAJB9
EDEM1
TTF2
ZMYM6
IGFBP3
THBS2S
FLNA
NR4A1
NCAM
GCNT2
ACTB
CREM
LAMA1
NR3C3
CASR
CD1
THBS1
IGF1
DUSP
MSX
TBC1D1
RCAN1
HOXB2
LAMA5
RASA1
glsA
GGT1
VCP
STAT5A
SQSTM1
PAX8
SNX1_2
LAMC3
RPS6KA
MCAM
SLC6A6S
ECM29
CTNNA
ARNT
TCF1
AKT
VEGF
DNM
ITGAM
TAC1
CEACAM
CTH
PSMA6
PIK3C
PIKFYVE
DLL
TRAP1
PIPK1
STAU
KMT2E
YWHAB
SIRPA
ROCK2
IDE
CSF2RB
SMARCC
GGT5
MAPT
GPT
HDAC7
SGK1
TST
LOC100691120
MYO7
SLC26A4
LOC101363017
ATP13A2
TG
PPP1C
VLDLR
PDK1
SHIP1
SYNJ2
PLCD
TAT
SRPK2
CCND1
RET
MYO5
RAPGEF3
CHP
NMYC
PIP5K
PDPK1
PIK3C2
PIK3C3
metK
PKN
GIT1
SH3GL
CRCP
DPEP
CAMK2
CAMK1
XPO1
HDAC4_5
SGMS
PTPRN
NDRG2
ADAMTS9
MDM2
PLCB
WNT11
UVRAG
HDAC11
CASP8
CDK
NDE1
FRS3
SLC7A9
LRP2
GATA3
CSDA
HDAC3
NDRG3
LGR5
JAZF1
SGOL1
ORC4
INPP5F
FZD1
DAAM
HDAC10
RFXANK
FGFR4
FZD5_8
CLP1
CPSF3
cpsf5
RANBP2
CCNC
CYP7B
DAAM
ESRRBL1
roeD
AIP5
TPR
KLK1
MCM2
CCNG1
RGN
BRMS1
CCNG2
ARIH1
PTOP1
FHIT
SFRS4_5_6
TRH-I
DHX38
pgm
HSD3B7
MYO6
WWP2
DCLRE1A
CBLL1
NUP205
ARHGAP6
ATBF1
MBT18
AAAS
PRC1
ADK
GINS4
MBD10
ARG2
PYCR1
PCAF
NUDT5
ANPRA
TKT
GEMIN6
frmB
MBD11
SNAPC4
SLC7A4
MLC1
MYLK
ACTC1
TWSG1
PDE11
ASPM
SPAG5
SUV39H2
purB
TADA2A
PDE1
RPC2
BRF2
K06874
CA2
PDE7
ADCY7
RPC5
CECR1
TPM1
ENPP1_3
LCP1
OPCML
UBIAD1
NT5C2
CHRDL2
pyrG
TNNI1
MB
PPCDC
SLC4A8
COMMD9
AQP2
cdd
udp
NUDT12
ATP6V0A
CMPK1
CA3
ATPeV1A
